# Transcriptomics, proteomics, metabolomics and network pharmacology reveal molecular mechanisms of multi‐targets effects of Shenxianshengmai improving human iPSC‐CMs beating

**DOI:** 10.1002/ctm2.1302

**Published:** 2023-06-06

**Authors:** Yiqing Hu, Lulan Chen, Shuang Zhao, Runyang Feng, Xin Cao, Geng Chen, Tao Zhao, Chi Zhang, Zheyan Fang, Zhenyang Guo, Xueting Yu, Zhentao Zhang, Mukaddas Abdurahman, Hangnan Hong, Yue He, Hua Li

**Affiliations:** ^1^ Department of Cardiology Shanghai Institute of Cardiovascular Diseases Shanghai Xuhui District Central Hospital & Zhongshan‐Xuhui Hospital Zhongshan Hospital Fudan University Shanghai China; ^2^ Department of Medical Examination Shanghai Xuhui District Central Hospital Shanghai China; ^3^ Institute of Clinical Science Zhongshan Hospital Fudan University Shanghai China; ^4^ Center for Bioinformatics and Computational Biology East China Normal University Shanghai China; ^5^ Shandong Buchang Pharmaceutical Co. Ltd. Heze Shandong China; ^6^ Jining Medical University Jining China; ^7^ Department of Cardiology Shanghai Eighth People's Hospital Shanghai China

Dear Editor,

Bradycardia is one of common cardiovascular diseases; however, effective oral drugs in clinic are rare. Shenxianshengmai (SXSM) is one traditional Chinese medicine licensed by National Medical Products Administration for it. In the study, multi‐omics approach will be used to deeply investigate its intracellular mechanism for better clinical application.

To verify the purity of hiPSC‐CMs, we utilized immunofluorescence and flow cytometry stained with cTnT or α‐actinin as well as manual patch clamp to assess their electrophysiological properties (Figure S1A–C). Subsequently, different diluted concentrations of SXSM original solution were selected for the pre‐treatment of human iPSC‐CMs (Figure S1D–L). Finally, 1 h pre‐treatment of SXSM with the concentration of 0.55 mg/mL was preliminarily chosen. As previous studies showed that the effects of SXSM were strongly associated with adrenoceptor‐related pathways,^1^, ^2^ isoproterenol (ISO) was used to compare with SXSM regarding the effect on human iPSC‐CMs (Figure S2A–M). With the treating time protracting, the SXSM group, instead of the ISO group, appeared more durable in the increase of beat rate (BR) and shortage of corrected field potential duration.

First, to elucidate the myocardial pharmacological function of SXSM, the potential chemical components of SXSM were identified by UPLC‐QTOF‐MS/MS and GC–MS in comparison with the standard TCM chemicals (Figure 1A,B), which was followed by network pharmacology analysis. In total, 262 candidate compounds were identified by combining 252 components from UPLC‐Q‐TOF/MS (Table S1) and 28 components (Table S2) from GC–MS after removing duplicates and isomers. Among them, 63 compounds were confirmed by comparison with the controls, and chemical structures of some representative identified monomer components were shown (Figure 1C, Table S3). Subsequently, based on the STRING database, protein interaction networks of components and potential targets of diseases (arrhythmia, myocardial ischaemia) were constructed (Figure S3A,B, Table S4), and then a 150 core targets’ cluster was generated (Table S5). GO‐BP/MF analysis (Figure S3C,D) suggested that SXSM might increase the intracellular phosphorylation level and modulate the intracellular energy metabolic environment. In the top 15 metabolic pathways of node degrees (Table S6), phosphorylation‐related signal transduction pathways were also identified.

**FIGURE 1:**
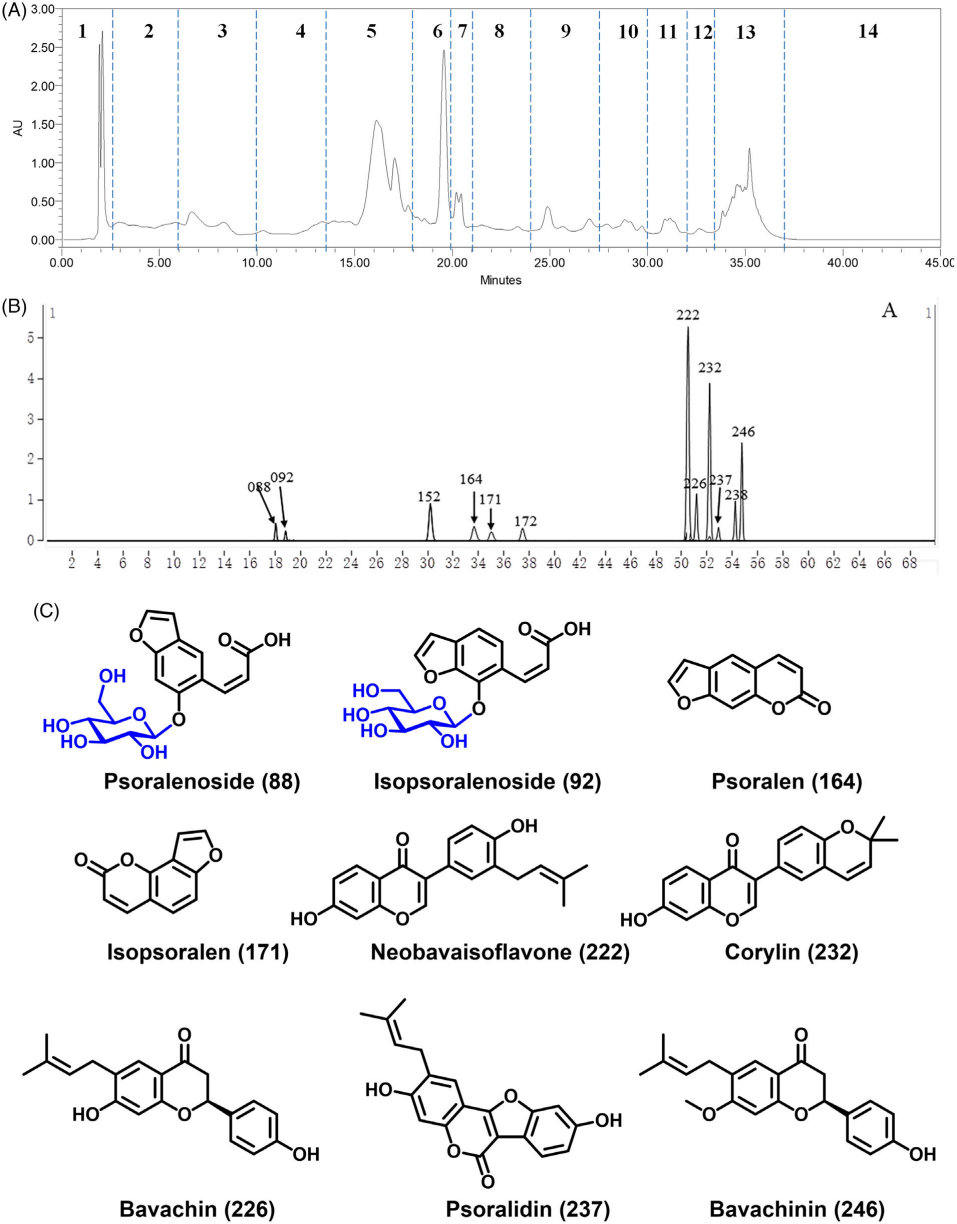
The identification flow of the monomer components from Shenxianshengmai (SXSM) by UPLC‐QTOF‐MS/MS in comparing with standard TCM chemicals. (A) Schematic diagram of first‐dimensional chromatographic fraction segmentation. The fractions collected in each part were evaporated to dryness and then reconstituted with a small amount of solvent to serve as a sample for second‐dimensional chromatography. Different parts of the samples obtained by the first‐dimensional chromatography entered the second‐dimensional chromatography for separation. The second‐dimensional chromatography was combined with mass spectrometry to directly complete mass spectrometry data acquisition. (B) Control mix chart of some representative identified monomer components. (C) Chemical structure of the reference substance of representative identified monomer components.

Subsequently, transcriptomics–proteomics–metabolomics revealed the molecular mechanism of SXSM improving the beating of human iPSC‐CMs (Supplementary Document 1). In transcriptomics, 345 differentially expressed (DE) genes were detected between control (CTRL) and SXSM group (Figure S4A, *p*‐value <0.05). By using GSEA based on GO database (gGSEA), the activity of the three calcium‐related pathways was found to increase (Figure 2A, *p*‐value <0.05). Besides calcium, other ions involved in the electrophysiological activity of human iPSC‐CMs were also observed in gGSEA (Figure S4B). Moreover, the mobilization of energy metabolism indicated in network pharmacology was also corroborated by gGSEA analysis (Figure 2B, *p*‐value <0.05). Meanwhile, structural units of myocardial contraction were also found to increase (Figure S4C). Consistent with the anti‐apoptotic effect shown in the network pharmacology, the pathways of myocardial apoptosis were suppressed (Figure 2C, *p*‐value <0.05). Among the significant down‐regulation pathways of GSEA based on the KEGG database (kGSEA), arachidonic acid metabolism had a significant down‐regulation trend (Figure 2D, *p*‐value <0.05). Additionally, SXSM could not induce pathological and non‐matching angiogenesis based on the Reactome database (Figure S4D,E). Between CTRL and SXSM, a few significantly differential alternative splicing (AS) events were detected (Figure S4F). Subsequently, KEGG pathway enrichment analysis for the genes obtained from each of the five AS forms was performed (Figure 2E), with the result of enriched pathways directly related to spliceosome, heart rate elevation, energy, calcium, phosphorylation and anti‐apoptosis. In proteomics, 190 DE proteins were detected between CTRL and SXSM group (*p*‐value <0.05 and fold change > 1.1 or <(1/1.1)) (Figure S5A–F). Subsequently, several pathways worthy of attention in the KEGG analysis of DE proteins were detected (Figure 2F, *p*‐value <0.05). In metabolomics, 1106 metabolites by combining positive (739) and negative (367) ion patterns were identified, including 13 main classes and other additional compounds (Figure S6A). Between CTRL and SXSM group, 138 metabolites (pos 110, neg 68) with significant differences (fold change > 1.2 or <(1/1.2) VIP > 1) were detected (Figure S6B). The DE metabolites were selected for KEGG and SMPDB analysis of Metabolite Set Enrichment Analysis and Metabolomic Pathway Analysis (Figure 2G).

**FIGURE 2:**
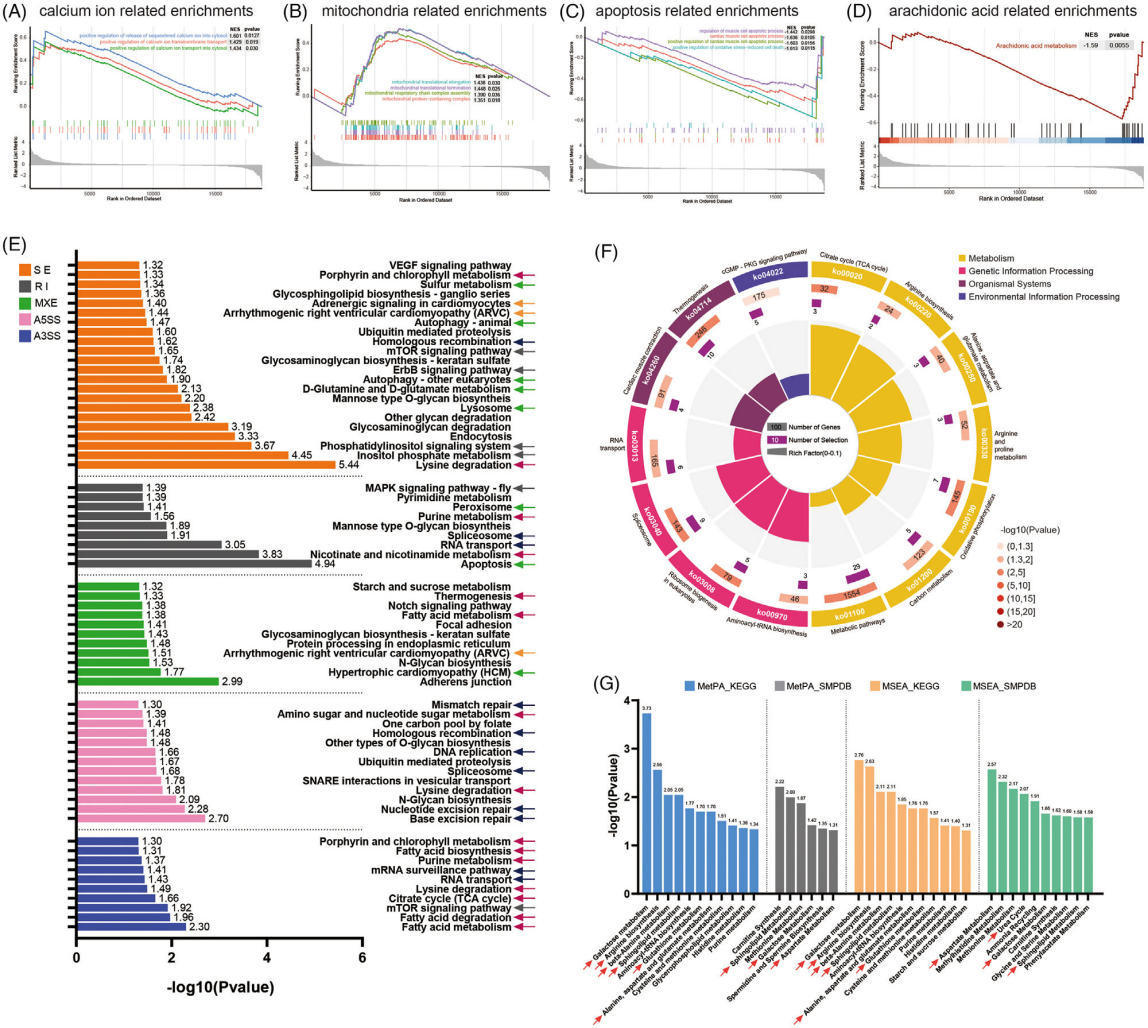
The analysis of transcriptomics, proteomics and metabolomics (CTRL vs. Shenxianshengmai [SXSM]). (A–D) Gene Set Enrichment Analysis (GSEA) of detected genes in gene ontology (GO) database. (E) KEGG enrichment analysis of five alternative splicing (AS) patterns. Selection criteria: *p*‐value <0.05. 5 AS: retained intron, RI; alternative 3′ splice site, A3SS; alternative 5′ splice site, A5SS; mutually exclusive exon, MXE; skipped exon, SE. Arrows: Dark blue arrow represents *AS or for pre‐translational regulation related*; yellow arrow represents *heart rate elevation‐related pathways*; green arrow represents *anti‐apoptotic and antioxidant‐related pathways*; red arrow represents *energy metabolism‐related pathways*. Grey arrow represents *calcium ion and phosphorylation‐related pathways*. (F) KEGG pathway analysis of differentially expressed (DE) proteins. Selection criteria: *p*‐value <0.05; fold change >1.1 or <(1/1.1). There are four circles from outside to inside. The first circle is the classification of KEGG enrichment, where different classifications are indicated by different colours. The small bands in the second circle are the number of this classification in the background proteins and the *p*‐value. The more proteins the longer the bar, the smaller the *p*‐value the redder the colour. The third circle is a bar chart of the detected proteins in correspondent KEGG pathways. The lower part shows the specific values. The fourth circle is the rich factor value for each classification (the number of foreground proteins divided by the number of background proteins in that classification). (G) KEGG and SMPDB analysis of Metabolite Set Enrichment Analysis (MSEA) and Metabolomic Pathway Analysis (MetPA). Selection criteria: *p*‐value <0.05; fold change >1.2 or <(1/1.2). The red arrows mark the pathways of concern.

Moreover, a joint analysis between network pharmacology and three omics was performed to elucidate molecular pathways (Supplementary Document 2). A two‐by‐two analysis in the ‘gene–protein–metabolite’ dimension was conducted: interaction network for AS events and proteins (Figure 3A), protein–metabolite correlation network analysis (Figure 3B) and Orthogonal Projections to Latent Structures (O2PLS)^3^, ^4^, ^5^, ^6^ for DE genes (*p*‐value <0.05) and DE metabolites (Figure 3C, Figure S6C,D).

**FIGURE 3:**
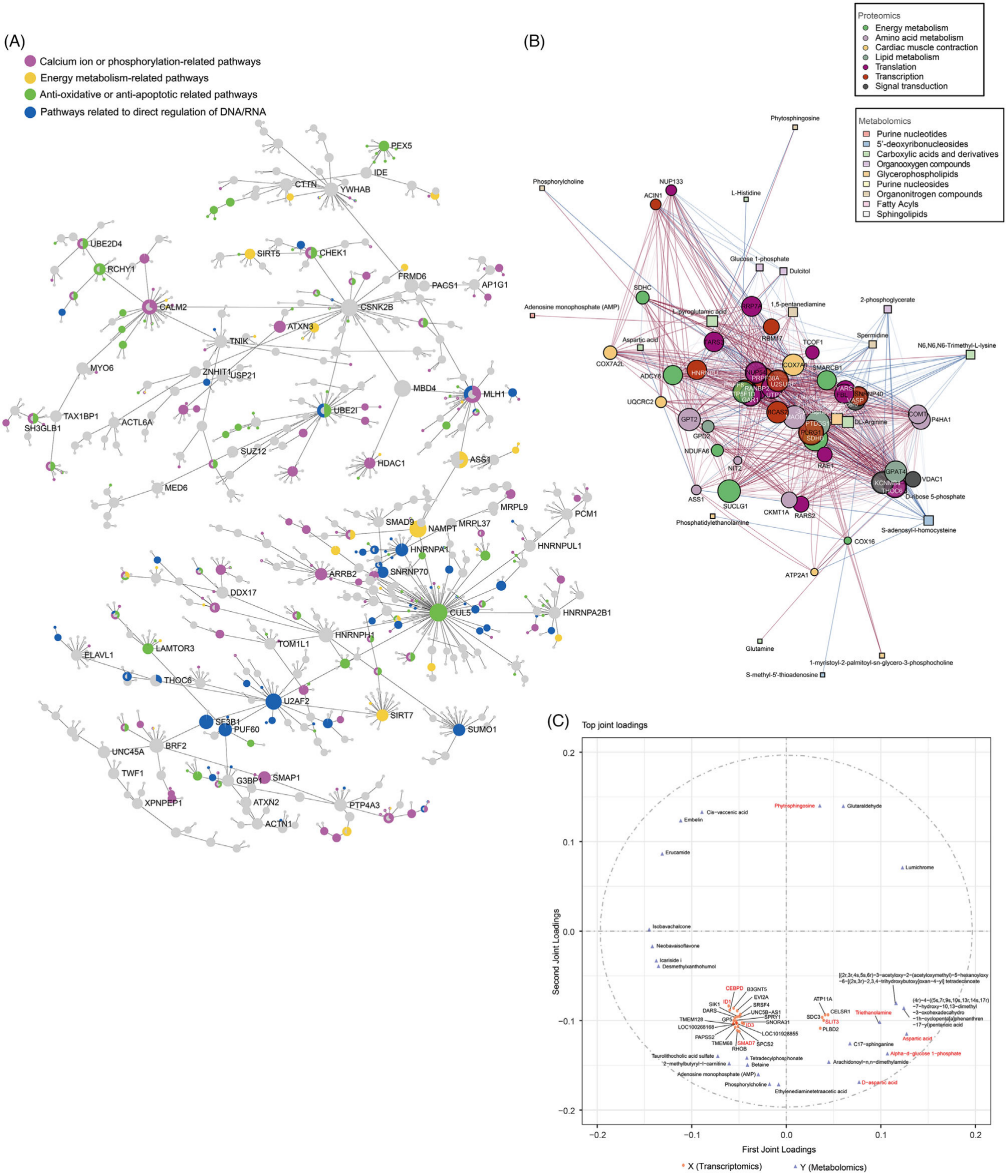
The tri‐omics joint analysis. (A) The interaction network of significant alternative splicing (AS) events and proteins at the ‘protein–protein’ and ‘TF‐genes’ levels were analysed. (B) Protein–metabolite correlation network. Differentially expressed (DE) proteins (duplicates removed) in the selected 14 of the top 20 KEGG pathways were used for protein–metabolite correlation network analysis to identify co‐regulated nodes, with all 29 DE metabolites opted from the enrichment pathways obtained from the above metabolite analysis at *p*‐value <0.05 (duplicates removed). Each node represented a protein (circle) or metabolite (square), and its size was proportional to its eigenvector centrality (network influence). Nodes colour coded according to functional classification of proteins and metabolic class, respectively. Nodes with more significant correlation were closer together. Nodes with a Pearson correlation coefficient of more than or equal to 0.7 constitute an edge (blue are positive correlations, and red are negative correlations), whose transparency was proportional to their *p*‐value, respectively. (C) O2PLS joint loadings plot of metabolites and genes in response to Shenxianshengmai (SXSM) treatment of human iPSC‐CMs. The cross‐validation method was used for multiple modelling to evaluate the set parameters (number of the joint and orthogonal components) for each dataset (DE genes and DE metabolites in metabolome) and the model with the lowest prediction error was selected for subsequent analysis. Genes (circles) and metabolites (triangles) represented individual gene and metabolite loading values. The loading value indicated the explanatory power of the variable (metabolite/gene) in each component (i.e. the contribution to the variation between groups), and a positive or negative loading value indicated a positive or negative correlation with another group, the larger the absolute value of the loading value, the stronger the association. The distance from each point to the origin in the loadings plot mean the magnitude of the correlation with another histology, the closer the factor (genes and metabolites) to the outer circle in the plot, the higher the correlation between the two histologists. Variables located to the left of the *y*‐axis represented genes and metabolites that decreased, whereas those on the right increased, compared to CTRL group in response to SXSM group.

Finally, multichannel validation of SXSM enhancing human iPSC‐CMs beating was performed. KN93 significantly reversed the effect of 0.55 mg/mL SXSM on BR elevation of human iPSC‐CMs at the concentration of 300 nM (106.0% ± 2.5%) and further inhibited Amp (80.5% ± 2.8%) (Figure 4A,B). Moreover, AIP pre‐treatment had no significant effect on either the BR or Amp changes of human iPSC‐CMs induced by SXSM (Figure 4C,D). Meanwhile, pCaMK of human iPSC‐CMs was decreased after 0.55 mg/mL SXSM 1 h treatment (Figure 4E). NCLX inhibitor CGP37157 ≥10 μM post‐treatment could suppress the elevation of BR of human iPSC‐CMs due to 0.55 mg/mL SXSM treatment for 1 h (Figure 4F,G). Furthermore, the inhibition of CaMKII by SXSM contributed to myocardial protection and the prevention of oxidative stress due to Ca^2+^ overload.^7^ Collectively, this suggests that 0.55 mg/mL SXSM treatment mainly triggers Ca^2+^‐CaM‐AC‐PKA, a pathway that activates the calcium pool (Figure 4H). Additionally, SXSM may influence the whole process of human iPSC‐CMs’ action potential at least through Nav1.5, Kir2.1 and Kv11.1 (Figure S7).

**FIGURE 4:**
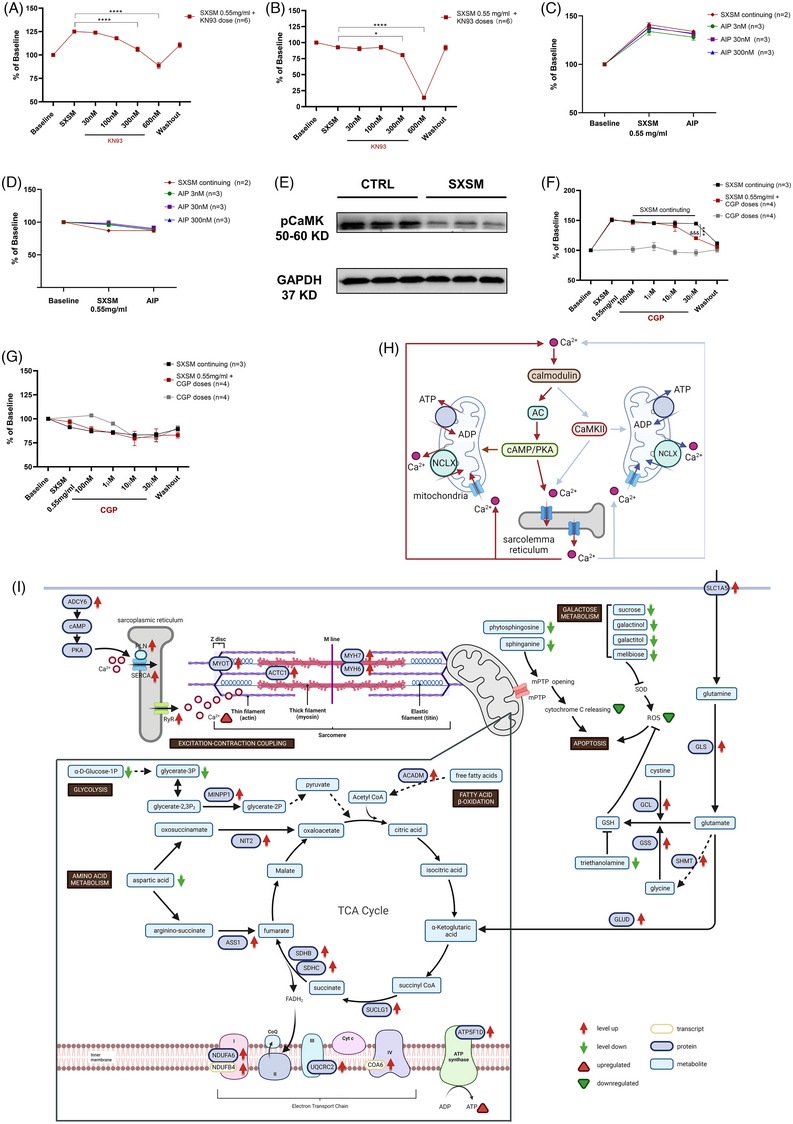
The validation of the calcium‐related signalling pathway and the joint analysis on the molecular mechanism in human iPSC‐CMs with 0.55 mg/mL Shenxianshengmai (SXSM). (A and B) Effects of different concentrations of KN93, inhibitor of CaM/CaMKII, on beat rate (BR)/Amp after pre‐treatment with 0.55 mg/mL SXSM in impedance mode. (A) BR; (B) Amp. *p < 0.05, **p < 0.01, ***p < 0.001, ****p < 0.0001 compared to the values of horizontal coordinate ‘SXSM’ (one‐way ANOVA). (C and D) Effects of different concentrations of autocamtide‐2‐related inhibitory peptide (AIP), inhibitor of CaMKII, on BR/Amp after pre‐treatment with 0.55 mg/mL SXSM in impedance mode. (C) BR; (D) Amp. (E) Western blotting analysis. Western blotting analysis of down‐regulated protein levels of pCaMK in human iPSC‐CMs in response to 0.55 mg/mL SXSM 1 h. Representative results from three independent experiments. (F and G) Effects of different concentrations of CGP‐37157, inhibitor of NCLX, on the BR/Amp after pre‐treatment with 0.55 mg/mL SXSM in impedance mode. (F) BR; (G) Amp. *p < 0.05, **p < 0.01, ***p < 0.001 comparison between groups. ^&^ *p* < 0.05, ^&&^ *p* < 0.01, ^&&&^ *p < 0*.001, compared to the point with the horizontal coordinate ‘SXSM .55 mg/mL’ in SXSM 0.55 mg/mL + CGP doses group (red) (one‐way ANOVA). (A–D, F and G) Each treatment was spaced 1 h apart. (H) Proposed Ca^2+^/CaM signalling pathway involved in the effects of 0.55 mg/mL SXSM 1 h on human iPSC‐CMs. (I) Summary of the joint analysis of the mechanisms. All data are presented as the mean ± SE. *Source*: Panels (H) and (I) were created with BioRender.com.

In sum, increased intracellular calcium cycling and intracellular calcium, and accelerated excitatory contractile coupling in human iPSC‐CMs by SXSM, which could coordinately lead to their faster beating. Meanwhile, SXSM promoted TCA cycling and oxidative phosphorylation through adequate mobilization of the glycolytic pathway, amino acid metabolism and fatty acid β‐oxidation to better supply energy to human iPSC‐CMs. Combined with the enhanced antioxidant pathway and reduced apoptosis in SXSM‐treated human iPSC‐CMs, SXSM implicated a cardioprotective effect (Figure 4I). In addition, the long‐term mechanism of SXSM and its protective effect on hypoxia and reoxygenation are also worthy of further exploration (Figure S8). As science and technology advance, the more precise mechanism of SXSM improving heartbeat will be demonstrated. This may offer alternative therapy options for arrhythmia patients who cannot have a pacemaker implanted.

## CONFLICT OF INTEREST STATEMENT

There is no conflict of interest involved in this article.

## Supporting information

Transcriptomics–proteomics–metabolomics in detail in this article are found in Supplementary Document 1.

A joint analysis among their omics in this article is found in Supplementary Document 2.

Materials and methods adopted in this article are found in Supplementary Document 3.

Table S1‐S6

Supporting Information

Supporting Information

Supporting Information

Supporting Information

Supporting Information

Supporting Information

Supporting Information

Supporting Information

Supporting Information
