# Supplementary material for: Transcriptomics, proteomics, metabolomics and network pharmacology reveal molecular mechanisms of multi‐targets effects of Shenxianshengmai improving human iPSC‐CMs beating
Source: Clin Transl Med. 2023 Jun 6;13(6):e1302. doi: 10.1002/ctm2.1302 (PMC10246690; doi:10.1002/ctm2.1302)
Supplement: Supplementary file 1 — Transcriptomics–proteomics–metabolomics in detail in this article are found in Supplementary Document 1. [file CTM2-13-e1302-s006.docx]

## Supplementary Document 1-

## Transcriptomics-proteomics-metabolomics Analysis

### 1. Transcriptomics

345 differentially expressed (DE) genes were detected between control groups (CTRL) and SXSM groups at the transcriptomic level. Meanwhile, gene set enrichment analysis (GSEA)^1^ was used to encapsulate as much detail as possible of the changes to amplify and pinpoint the clues provided in the network pharmacology. After above two data crosses, the suggestion of calcium signaling pathways, a regulator strongly associated with the phenotype of heartbeat, in network pharmacology was corroborated in transcriptomics. By using GSEA based on the gene ontology (GO) database (gGSEA), activity of the three calcium-related pathways was found to increase: positive regulation of release of sequestered calcium ion into the cytosol, positive regulation of calcium ion transmembrane transport, and positive regulation of calcium ion transport into the cytosol. Besides calcium, other ions involved in the electrophysiological activity of human iPSC-CMs were also observed in gGSEA: the reduced activity of potassium leak channels and decreased stability of the cell membrane potential under the maintenance of sodium homeostasis, which might predict a decrease in the absolute value of the resting potential of human iPSC-CMs and a decrease in the excitation threshold. Collectively, human iPSC-CMs with SXSM have more active endogenous dynamics of calcium and have a higher sensitivity to trigger beatings under exogenous stimuli. What's more, as a supplier of ATP required for excitation contraction coupling, the mobilization of energy metabolism indicated in network pharmacology was also corroborated by GSEA analysis, which showed that mitochondrial translational elongation, mitochondrial translational termination, mitochondrial respiratory chain complex assembly and mitochondrial protein-containing complex were simultaneously activated in 0.55mg/ml SXSM groups. Meanwhile, structural units of myocardial contraction were also found to enrich with a tendency to be elevated. Overall, the increased intracellular calcium ion concentration, the accelerated calcium cycle and the full energy mobilization might synergistically improve excitability and solid contractile material units to contribute to their faster beating frequency.

The increasement of heartbeat frequency was highly associated with myocardial hypertrophy and apoptosis in the decompensated state. In this study, consistent with the anti-apoptotic effect shown in the network pharmacology, the pathways of myocardial apoptosis were suppressed, such as regulation of muscle cell apoptotic process, cardiac muscle cell apoptotic process, positive regulation of cardiac muscle cell apoptotic process and positive regulation of oxidative stress-induced cell death. As for myocardial hypertrophy, arachidonic acid (AA) and other nonesterified fatty acids (FAs) have been found to introduce a profound intracellular acidosis and have negative consequences during myocardial ischemia^2^. Under oxidative stress conditions such as ROS, Free AA is easily oxidized by reactive molecules to eicosanoids (such as isoprostanes and nitroeicosatetraenoic acids) leading to cardiomyocyte hypertrophy^3^. The consistent observation is that among the significant down-regulation pathways of GSEA based on KEGG database (kGSEA), arachidonic acid metabolism had a significant down-regulation trend, while AA (peroxide free) appeared downregulated (fold change = 0.81, VIP = 7.29) in the latter metabolomics. Additionally, SXSM couldn’t induce pathological and non-matching angiogenesis and was less likely to induce correspondent pathological myocardial hypertrophy in human iPSC-CMs^4^, as also shown by GSEA analysis based on GO database and Reactome database.

Alternative splicing (AS) allows an uncut RNA to have different patterns of exon shearing and splicing, thus allowing a gene to produce multiple types of proteins to adapt to specific time and environmental changes. Between CTRL and SXSM, a few significantly differential AS events were detected. Subsequently, KEGG pathway enrichment analysis for the genes obtained from each of the five AS forms was performed, with the result that pathways directly related to AS or for pre-translational regulation (e.g., spliceosome) were enriched, especially in the alternative 5' splice site (A5SS), which matched the higher number of significant genes obtained from AS analysis. Meanwhile, an enrichment of pathways associated with heart rate elevation events (such as adrenergic signaling in cardiomyocytes in SE) was observed in AS analysis. And energy-related (e.g., citrate cycle (TCA cycle), fatty acid metabolism), calcium- and phosphorylation-related (e.g., phosphatidylinositol signaling system, inositol phosphate metabolism) and anti-apoptosis-related (e.g., apoptosis, peroxisome) pathways were similarly enriched (*p*-value <0.05).

### 2. Proteomics

190 proteins with significant expression differences were detected between group CTRL and SXSM (*p*-value <0.05 and fold change > 1.1 or < (1/1.1)). Based on these proteins, several pathways worthy of attention in KEGG analysis of DE proteins were found: i) energy metabolism-related pathways: thermogenesis, oxidative phosphorylation, citrate cycle (TCA cycle), alanine, aspartate and glutamate metabolism, arginine biosynthesis, arginine and proline metabolism; ii) calcium ion or phosphorylation-related pathways: GMP - PKG signaling pathway, cardiac muscle contraction; iii) pathways related to direct regulation of DNA/RNA: Spliceosome, RNA transport. Among them, cardiac muscle contraction and cGMP - PKG signaling pathway was correlated with the results of intracellular calcium ion of human iPSC-CMs in transcriptomics and in line with the phenotype of increasing heartbeat. Previous studies showed that intracellular calcium activity was closely related to the phosphorylation of proteins: increased ADCY6 raised the levels of the proteins p-RyR2, p-PLN and Ca^2+^ peak concentration^5,6^, which could be mediated by cAMP/PKA^7^. In this study, ADCY6 (fold change = 1.14, *p*-value = 0.045) was found to elevate along with RyR2 and PLN, which was consistent with the increased levels of phosphorylation in human iPSC-CMs predicted by network pharmacology and analysis in transcripts. Sarcoplasmic reticulum (SR) could regulate Ca^2+^: releasing or absorbing Ca^2+^ via the ryanodine receptor 2 (RyR2) or sarcoplasmic/endoplasmic reticulum Ca^2+^ ATPase 2a (SERCA2a), respectively^8^. Our results demonstrated that ATP2A homologous genes (ATP2A1 and ATP2A2) encoding SERCA were upregulated. Excitable and depolarized cardiomyocytes or increased intracellular calcium concentrations in cardiomyocytes as mentioned above resulted in the elevation of KCNMB4 (fold change = 1.38, *p*-value = 0.018), which could expel intracellular potassium ions to stabilize the membrane. Additionally, the sarcomere, the structural component that drives cardiac contraction, was stabilized, which was in line with the prediction of transcriptomics. And MYOT (fold change = 1.73, *p-value* = 0.042), regulating myofibril formation and stability at the Z lines in muscle cells^9,10^, and Sarcomeric actomyosins (MYH6, MYH7, and ACTC1) was also found to be increased. These indicated that the structural basis of human iPSC-CMs was stabilized and the excitatory contraction coupling was enhanced under the condition of XSXM administration.

Energy mobilization of SXSM on human iPSC-CMs was revealed by two KEGG pathways in proteomics: oxidative phosphorylation and citrate cycle (TCA cycle), mentioned in the above analysis of AS and network pharmacology. In the experiment, 0.55mg/ml SXSM for 1h promoted human iPSC-CMs’ beating without affecting their beating amplitude, which required more energy to meet a relatively high metabolic demand. The experimental data showed that protein SUCLG1, SDHB, SDHC in TCA cycle were observed to be increased (*p*-value < 0.05) and proteins (enzymes) in the respiratory chain tended to be activated to participate in oxidative phosphorylation. Gene NDUFB4 and protein NDUFA6, accessory subunits of the mitochondrial membrane respiratory chain NADH dehydrogenase (complex I)^11,12^, were found to be upregulated (*p*-value < 0.05). And protein UQCRC2, a core subunit of the ubiquinol-cytochrome c oxidoreductase (cytochrome b-c1 complex, complex III, CIII) in mitochondrial electron transport chain and preserves the processing properties of mitochondrial-processing peptidase^13^, was also increased (*p*-value < 0.05). Furthermore, COA6, involved in the maturation of the mitochondrial respiratory chain complex IV subunit MT-CO2/COX2 to regulate early steps of complex IV assembly^14,15^ was increased (*p*-value < 0.05). And ATP5F1D (fold change = 1.24, *p*-value = 0.005), as the mitochondrial membrane ATP synthase (F1F0 ATP synthase or Complex V) was also rising, which could improve the production of ATP from ADP following the procedure of electron transport complexes of the respiratory chain^16^. Based on these, human iPSC-CMs with SXSM sped up the TCA cycle and dispensed a more efficient electron transport chain to generate more ATP for energy supply. Noticeably, the splicesome pathway (ko03040) was enriched in both transcriptomic AS (A5SS, RI) and proteomic KEGG analysis. And SF3B1, contributing to the shift in energy metabolism from FAO to glycolysis in cardiomyocytes^17^, was detected in transcriptomic AS and was elevated in proteomics.

### 3. Metabolomics

1106 metabolites by combining positive (739) and negative (367) ion patterns were identified, including 13 main classes and other additional compounds. Between CTRL and SXSM groups (fold change > 1.2 or < (1/1.2) and VIP >1, 138 metabolites (pos 110, neg 68) with significant differences were detected. The clustering heat map showed that the DE metabolites between different biological replicates were clustered together, which indicated good consistency between biological replicates and the reliability of the data. Then, significant DE metabolites were selected for KEGG and SMPDB analysis of Metabolite Set Enrichment Analysis (MSEA) and Metabolomic Pathway Analysis (MetPA). Considering that apoptosis or aging is closely related to the accumulation of metabolic wastes or oxidative stress, anti-aging and anti-apoptosis pathways were firstly discussed, with the result of related pathways enriched, such as galactose metabolism (ko00052, SMP0000043), glutathione metabolism (ko00480) and sphingolipid metabolism (ko00600, SMP0000034). Next, previous KEGG analysis of the proteome suggested the possibility of energy mobilization in myocardium, however, didn’t gave a clear indication of the specific metabolites (e.g., sugars, proteins, and lipids). In this section, analysis of DE metabolites could give us the precise clue with the following pathways related to the tricarboxylic acid (TCA) cycle and energy metabolism enriched: arginine biosynthesis (ko00220), urea cycle (SMP0000059), alanine, aspartate and glutamate metabolism (ko00250), aspartate metabolism (SMP0000067), and beta-Alanine metabolism (ko00410). Among them, both pathways of alanine, aspartate, and glutamate metabolism and arginine biosynthesis were enriched in KEGG analysis of the proteome and the metabolome.

# References

1. Subramanian A, Tamayo P, Mootha VK, et al. Gene set enrichment analysis: a knowledge-based approach for interpreting genome-wide expression profiles. *Proc Natl Acad Sci U S A.* 2005;102(43):15545-15550.

2. Wu ML, Chan CC, Su MJ. Possible mechanism(s) of arachidonic acid-induced intracellular acidosis in rat cardiac myocytes. *Circ Res.* 2000;86(3):E55-62.

3. Sonnweber T, Pizzini A, Nairz M, Weiss G, Tancevski I. Arachidonic Acid Metabolites in Cardiovascular and Metabolic Diseases. *Int J Mol Sci.* 2018;19(11).

4. Hu Y, Lu H, Li H, Ge J. Molecular basis and clinical implications of HIFs in cardiovascular diseases. *Trends Mol Med.* 2022;28(11):916-938.

5. Deng Y, Wang J, Xie G, Zeng X, Li H. Circ-HIPK3 Strengthens the Effects of Adrenaline in Heart Failure by MiR-17-3p - ADCY6 Axis. *Int J Biol Sci.* 2019;15(11):2484-2496.

6. Nofi C, Zhang K, Tang YD, et al. Chronic dantrolene treatment attenuates cardiac dysfunction and reduces atrial fibrillation inducibility in a rat myocardial infarction heart failure model. *Heart Rhythm O2.* 2020;1(2):126-135.

7. Chebib FT, Sussman CR, Wang X, Harris PC, Torres VE. Vasopressin and disruption of calcium signalling in polycystic kidney disease. *Nat Rev Nephrol.* 2015;11(8):451-464.

8. Lipskaia L, Chemaly ER, Hadri L, Lompre AM, Hajjar RJ. Sarcoplasmic reticulum Ca(2+) ATPase as a therapeutic target for heart failure. *Expert Opin Biol Ther.* 2010;10(1):29-41.

9. Salmikangas P, van der Ven PF, Lalowski M, et al. Myotilin, the limb-girdle muscular dystrophy 1A (LGMD1A) protein, cross-links actin filaments and controls sarcomere assembly. *Hum Mol Genet.* 2003;12(2):189-203.

10. Tucker NR, Chaffin M, Fleming SJ, et al. Transcriptional and Cellular Diversity of the Human Heart. *Circulation.* 2020;142(5):466-482.

11. Stroud DA, Surgenor EE, Formosa LE, et al. Accessory subunits are integral for assembly and function of human mitochondrial complex I. *Nature.* 2016;538(7623):123-126.

12. Alston CL, Heidler J, Dibley MG, et al. Bi-allelic Mutations in NDUFA6 Establish Its Role in Early-Onset Isolated Mitochondrial Complex I Deficiency. *Am J Hum Genet.* 2018;103(4):592-601.

13. Fernandez-Vizarra E, Zeviani M. Mitochondrial complex III Rieske Fe-S protein processing and assembly. *Cell Cycle.* 2018;17(6):681-687.

14. Pacheu-Grau D, Bareth B, Dudek J, et al. Cooperation between COA6 and SCO2 in COX2 maturation during cytochrome c oxidase assembly links two mitochondrial cardiomyopathies. *Cell Metab.* 2015;21(6):823-833.

15. Stroud DA, Maher MJ, Lindau C, et al. COA6 is a mitochondrial complex IV assembly factor critical for biogenesis of mtDNA-encoded COX2. *Hum Mol Genet.* 2015;24(19):5404-5415.

16. Oláhová M, Yoon WH, Thompson K, et al. Biallelic Mutations in ATP5F1D, which Encodes a Subunit of ATP Synthase, Cause a Metabolic Disorder. *Am J Hum Genet.* 2018;102(3):494-504.

17. Mirtschink P, Krishnan J, Grimm F, et al. HIF-driven SF3B1 induces KHK-C to enforce fructolysis and heart disease. *Nature.* 2015;522(7557):444-449.
