# Supplementary material for: Transcriptomics, proteomics, metabolomics and network pharmacology reveal molecular mechanisms of multi‐targets effects of Shenxianshengmai improving human iPSC‐CMs beating
Source: Clin Transl Med. 2023 Jun 6;13(6):e1302. doi: 10.1002/ctm2.1302 (PMC10246690; doi:10.1002/ctm2.1302)
Supplement: Supplementary file 2 — A joint analysis among their omics in this article is found in Supplementary Document 2. [file CTM2-13-e1302-s009.docx]

**Supplementary Document 2-Joint tri-omics analysis**

**Aspartic acid**

In amino acid metabolism, aspartic acid serves physiologically to boost mitochondrial energy generation under stress conditions and contributes to restocking the TCA cycle, hence adopting an important pro-energy function^1^. In this study, the aspartic acid was ranked first in metabolite loading values and labeled at the protein-metabolite correlation network. And the decrease in aspartic acid (fold change = 0.75, VIP = 1.26) in metabolomics implied that human iPSC-CMs employed pre-stocked amino acids for power generation. NIT2 and ASS1, being nodes in the protein-metabolite correlation network, were upregulated in the proteome (*p*-value < 0.05). According to alanine, aspartate and glutamate metabolism (ko00250) enriched in proteomics, NIT2 and ASS1 could induce aspartic acid into TCA for energy supply, which further explained the decrease in aspartic acid. Although the overall energy metabolism of human iPSC-CMs was upregulated in SXSM groups to activate the glucose metabolic pathway, utilization and consumption of aspartate ensured its FAO share of myocardial energy metabolism and simultaneously inhibited its metabolic shift to pathological synthetic pathways (e.g., PPP), thereby preventing excessive synthesis of biomass, myocardial hypertrophy, and secondary further energy overconsumption^2^. It was undoubted that the intrinsic FAO in human iPSC-CMs continued to be viable, no matter how the infusion of energy into human iPSC-CMs from gluconeogenesis and amino acid metabolism.

Additionally, one of the acyl-CoA dehydrogenases involved in the initial step of mitochondrial fatty acid beta-oxidation is protein ACADM^3^, which was detected to be elevated (*p*-value < 0.05).

**Galactose**

Although melibiose was not labeled on the graph, its loading value was located at the 26th position. Active galactose metabolism can speed up the aging of the heart^4^. Galactose reductase may convert excess d-galactose to galactitol to cause osmotic stress. Instead, galactose oxidase could oxidize d-galactose to hydrogen peroxide to reduce antioxidant enzymes (SOD). Besides, d-galactose could cause non-enzymatic glycation processes that generate advanced glycation end products (AGEs) after weeks or months, then via RAGEs (AGEs' receptors) to activate NADPH oxidase, finally resulting in ROS generation^4,5^. In this study, metabolites detected in galactose metabolism (sucrose, galactinol, galactitol, melibiose) were down-regulated, suggesting that SXSM inhibited the galactose metabolic pathway and hence postponed the malfunction of human iPSC-CMs.

**Glutamine**

Triethanolamine was in the top 25 metabolite loading values. Decreased triethanolamine (fold change = 0.31, VIP = 1.16) may release the block of the sulfhydryl residue of GSH and subsequently raise anti-oxidative power^6^. GSH can be raised by glutamine supplementation, with result of reduced myocardial lipid peroxidation and peroxynitrite levels to increase catalase enzyme activity^7^. Physiologically, glutamine utilization in the heart is not high^2^, while under stress conditions, cardiomyocytes are more likely to utilize the increased glutamine for anti-oxidative defense and electrical activity^8^. In this study, glutamine was observed to be increased (fold change = 2.00, VIP = 1.40) in the metabolome and had its position in the protein-metabolite correlation network. Moreover, protein SLC1A5, GLS, GLUD1, GCLM, SHMT2, and GSS in glutamine metabolism were detected to be increased ^8,9^. And Smad7 was also observed to be increased in the transcriptome (fold change = 1.24, p 0.039), with its first rank in gene loading values of O2PLS analysis, which might further explain glutamine metabolism may be cardioprotective^10^, like by reducing TGF-β1-Smad3 expression.

**Anti-apoptosis**

SXSM can prevent human iPSC-CMs apoptosis in other ways. In H/R damage, the buildup of phytosphingosine and sphinganine may promote mitochondrial permeability transition pore (mPTP) opening and cell death, which is regarded as the most likely characteristic^11^. In this analysis, decreased phytosphingosine (fold change = 0.66, VIP = 1.09) and sphinganine (fold change = 0.74, VIP = 9.84) in sphingolipid metabolism could preclude mPTP opening, restrict pro-apoptotic Cytochrome C leakage from mitochondria and preserve myocardial energy metabolism. Meanwhile, other factors related to anti-apoptosis and anti-hypertrophy (genes CEBPD^12,13^, ID1 and ID3^14^, SRSF4^15^, and SLIT3^16^) were also detected, which could limit oxidative stress, reduce pathological remodeling and constrain senescence and apoptosis.

# References

1. Aquilani, R., La Rovere, M.T., Corbellini, D., et al. (2017). Plasma Amino Acid Abnormalities in Chronic Heart Failure. Mechanisms, Potential Risks and Targets in Human Myocardium Metabolism. Nutrients ***9***, 10.3390/nu9111251.

2. Ritterhoff, J., Young, S., Villet, O., et al. (2020). Metabolic Remodeling Promotes Cardiac Hypertrophy by Directing Glucose to Aspartate Biosynthesis. Circ Res ***126***, 182-196, 10.1161/circresaha.119.315483.

3. He, M., Pei, Z., Mohsen, A.W., et al. (2011). Identification and characterization of new long chain acyl-CoA dehydrogenases. Mol Genet Metab ***102***, 418-429, 10.1016/j.ymgme.2010.12.005.

4. Bo-Htay, C., Palee, S., Apaijai, N., et al. (2018). Effects of d-galactose-induced ageing on the heart and its potential interventions. J Cell Mol Med ***22***, 1392-1410, 10.1111/jcmm.13472.

5. Lay, I.S., Kuo, W.W., Shibu, M.A., et al. (2021). Exercise training restores IGFIR survival signaling in d-galactose induced-aging rats to suppress cardiac apoptosis. J Adv Res ***28***, 35-41, 10.1016/j.jare.2020.06.015.

6. Peng, Y.W., Buller, C.L., and Charpie, J.R. (2011). Impact of N-acetylcysteine on neonatal cardiomyocyte ischemia-reperfusion injury. Pediatr Res ***70***, 61-66, 10.1203/PDR.0b013e31821b1a92.

7. Todorova, V.K., Kaufmann, Y., Hennings, L., and Klimberg, V.S. (2010). Oral glutamine protects against acute doxorubicin-induced cardiotoxicity of tumor-bearing rats. J Nutr ***140***, 44-48, 10.3945/jn.109.113415.

8. Shen, Y., Zhang, Y., Li, W., et al. (2021). Glutamine metabolism: from proliferating cells to cardiomyocytes. Metabolism ***121***, 154778, 10.1016/j.metabol.2021.154778.

9. Franklin, C.C., Backos, D.S., Mohar, I., et al. (2009). Structure, function, and post-translational regulation of the catalytic and modifier subunits of glutamate cysteine ligase. Mol Aspects Med ***30***, 86-98, 10.1016/j.mam.2008.08.009.

10. Zhang, H., Cui, Y.C., Li, K., et al. (2016). Glutamine protects cardiomyocytes from hypoxia/reoxygenation injury under high glucose conditions through inhibition of the transforming growth factor-β1-Smad3 pathway. Arch Biochem Biophys ***596***, 43-50, 10.1016/j.abb.2016.03.003.

11. Sun, L., Jia, H., Ma, L., et al. (2018). Metabolic profiling of hypoxia/reoxygenation injury in H9c2 cells reveals the accumulation of phytosphingosine and the vital role of Dan-Shen in Xin-Ke-Shu. Phytomedicine ***49***, 83-94, 10.1016/j.phymed.2018.06.026.

12. Huang, H., Luo, B., Wang, B., et al. (2018). Identification of Potential Gene Interactions in Heart Failure Caused by Idiopathic Dilated Cardiomyopathy. Med Sci Monit ***24***, 7697-7709, 10.12659/msm.912984.

13. Herrer, I., Roselló-Lletí, E., Ortega, A., et al. (2015). Gene expression network analysis reveals new transcriptional regulators as novel factors in human ischemic cardiomyopathy. BMC Med Genomics ***8***, 14, 10.1186/s12920-015-0088-y.

14. Du, M., Jiang, H., Liu, H., et al. (2022). Single-cell RNA sequencing reveals that BMPR2 mutation regulates right ventricular function via ID genes. Eur Respir J ***60***, 10.1183/13993003.00327-2021.

15. Larrasa-Alonso, J., Villalba-Orero, M., Martí-Gómez, C., et al. (2021). The SRSF4-GAS5-Glucocorticoid Receptor Axis Regulates Ventricular Hypertrophy. Circ Res ***129***, 669-683, 10.1161/circresaha.120.318577.

16. Gong, L., Wang, S., Shen, L., et al. (2020). SLIT3 deficiency attenuates pressure overload-induced cardiac fibrosis and remodeling. JCI Insight ***5***, 10.1172/jci.insight.136852.
