# Supplementary material for: Transcriptomics, proteomics, metabolomics and network pharmacology reveal molecular mechanisms of multi‐targets effects of Shenxianshengmai improving human iPSC‐CMs beating
Source: Clin Transl Med. 2023 Jun 6;13(6):e1302. doi: 10.1002/ctm2.1302 (PMC10246690; doi:10.1002/ctm2.1302)
Supplement: Supplementary file 3 — Materials and methods adopted in this article are found in Supplementary Document 3. [file CTM2-13-e1302-s002.docx]

**Supplementary Document 3 – Materials and Methods**

## Identification of human iPSC-CMs

The frozen human iPSC-CMs were resuscitated to 6-well plates for quality control. 24-48 hours after cardiomyocyte resuscitation, the resumption of pulsation of human iPSC-CMs was observed microscopically. Human iPSC-CMs were continued to be cultured for one week, and the supernatant was taken for bacterial, fungal and mycoplasma detection (Excell bio, MB000-1591). At the same time, human iPSC-CMs were digested with trypsin-EDTA (0.25%) (Gibco, 25200056), and a portion of the cells was collected for cell purity identification using flow cytometry fraction with identifying marker cTnT (BD, 564767); the remaining portion was then inoculated into 24-well plates prepared for double immunofluorescence staining of cTnT (abcam, ab8295) or α-Actinin (abcam, ab68194), and Hoechst. For flow cytometry fraction analyses, stained cells were then analyzed by flow cytometry (FACS Canto II; BD, San Jose, CA, USA). For double immunofluorescence staining, cells were visualized under a fluorescence microscope (Olympus).

## Electrophysiological testing

Spontaneous action potential of human iPSC-CMs was recorded by manual whole-cell recording. The microelectrode resistance was between 2-5 MΩ after filling with internal solution (KCl 130, NaCl 10, MgCl2 0.5, Mg-ATP 5, EGTA 0.5, HEPES 10, and Tris GTP 0.4 in mM, pH 7.2). The resumption of human iPSC-CMs were placed in the bath solution (NaCl 137, KCl 4, MgCl2 1, CaCl2 1.8, HEPES 10 and Glucose 10 in mM, pH 7.4). Individual human iPSC-CM was attached with the tip of electrode to form GΩ resistance seal by application of negative pressure and then form whole-cell recording by gently rupturing the membrane. After compensating for series resistance and membrane capacitance, then filtering at 6 KHz, electrophysiological characteristics of human iPSC-CMs spontaneous action potential was recorded in current-clamp. The patch clamp recording system used Axopatch200B amplifier, 1440A digital-to-analog converter and pClamp10.6 acquisition and analysis software (Molecular devices, USA). The software Origin Pro 8.0 (OriginLab) was used for data analysis. All experimental data were expressed as mean ± standard error (mean ± SE).

## Contractility and extracellular field potential measurement

Plates preparation and human iPSC-CMs seeding. CardioExcyte96 (Nanion Technologies) was used to assess the impedance (IMP) of human iPSC-CMs to examine pharmaceutical effects on contractility and coordinated ion channel activity via extracellular field potential (EFP) recordings. Two different types of CardioExcyte96 sensor plates (NSP-96) with a 2 mm or a 0.6 mm central recording electrode were used for impedance or EFP mode recording, respectively. human iPSC-CMs were thawed, plated and maintained on the NSP-96 according to the recommendations from cell supplier (Help Stem Cell Innovations, Nanjing, China, HELP4110). Briefly, 1% Fibronectin phosphate-buffered saline (PBS) solution was applied on NSP-96 (10 μg/ml per well) for 2-4hrs at 37℃ and removed before seeding. Human iPSC-CMs (Help Stem Cell Innovations, Nanjing, China, HELP4110) were thawed and planted evenly at a density of 40,000–60,000 viable cells per well on the NSP-96 to ensure the development of a monolayer with synchronous beating. Half medium was exchanged each day during the 5-7 days incubation period. Before on-board evaluation of stable baseline beating. Culture media was completely altered to ensure an exact volume per well 2 h before the assay on the day of the measurement of baseline and compounds application. During on-board evaluation (baseline, compounds treatment, hypoxia/reoxygenation condition), microenvironment for maintaining status of human iPSC-CMs including temperature, humidity, and gas mix is supervised and modulated by CE96 incubation system.

## Chemical reagents

Shenxian-shengmai original solution (SXSM, License number: Z20080183; Lot number: 21010102) are provided by Buchang Pharmaceutical (Shangdong, China); Isoproterenol hydrochloride (non-selective β-adrenergic receptor agonist), Autocamptide-2 Related Inhibitor Peptide (AIP, selective inhibitor of CaMKII), Propranolol hydrochloride (nonselective β-adrenergic receptor antagonist), and chromanol 293B (selective inhibitor of Kv7.1) are purchased from Sigma-Aldrich; Atenolol (cardioselective β1-adrenergic receptor antagonist), KN93(inhibitor of CaM/CaMKII), CGP37157 (selective inhibitor of Na^+^/Ca^2+^ exchanger), ML133 hydrochloride (selective Kir2 family channels inhibitor), Dofetilide (hERG channel blocker), and Flecainide acetate (Nav1.5 channel blocker) are purchased from Medchemexpress. Prior compounds addition, 1000- fold of the serial target concentrations of compounds were first diluted from stock solutions in DMSO, and then further diluted in culture medium to ensure the final DMSO concentration in each well is 0.1% after addition. Measurements of impedance and EFP were taken every 10 min for 20 s for a period of 1h after application of each concentration of compounds.

## Data acquisition and statistical analysis

The software CardioExcyte 96 Control (Nanion Technologies GmbH) was utilized to monitor the spontaneous beating of all human iPSC-CMs used in this research. Data analysis was performed using DataControl 96 (Nanion Technologies GmbH). The impedance and EFP modes both used a mean beat generated by aligning individual beats and computing mean beat. Parameters such as beat rate, amplitude (IMP mode) and FPD (EFP mode) were automatically detected. The Fridericia formula was used to calculate rate-corrected FPD (FPDc)^1^. Data represents as mean$\pm$SE. Statistical comparisons were done using student t test, one sample test, or one-way ANOVA with Dunnett’s post hoc analysis.

## The culture of human iPSC-CMs and sample preparation for multi-omics testing

Human iPSC-CMs are commercially available (Help Stem Cell Innovations, Nanjing, China, HELP4110). The purity of human iPSC-CMs is > 98%. The human iPSC-CMs (~5×10^6^ cells) were thawed and seeded on a 10cm Petri dish (~5 × 10^6^ cells/dish). The human iPSC-CMs were allowed to adhere for 24–48 h prior to replacement of the medium with fresh cardiac maintenance medium (Help Stem Cell Innovations) followed by half medium change each day. The human iPSC-CMs were incubated for 5-7 days in a 5% CO_2_/37°C environment until forming monolayer with stable synchronous beating. In each experiment, we set up two groups: the control group (medium 1h) and the SXSM-treated group (0.55mg/ml SXSM 1h). Each group had five 10cm Petri dishes cultured with human iPSC-CMs, respectively. Once reaching to treatment time, the medium in each dish was immediately discarded and washed three times with 1×PBS pre-cooled at 4°C. Subsequently, each group of cells was collected into two 50 mL centrifuge tubes separately with Cell Scrapers and mixed them well. The cell suspensions in 50mL centrifuge tubes of each group were then distributed to three new 15mL centrifuge tubes in a volume ratio of 2:2:1, centrifuged at 4℃ 1000rpm for 15min, then supernatants were removed, and cells were snap frozen in liquid nitrogen, and then used for transcriptome (~1×10^7^cells), metabolome (~1×10^7^cells) and proteome (~0.5× 10^7^cells) for testing. The whole experiment was repeated three times.

## Network pharmacology analysis

### Selection of candidate compounds

262 candidate compounds were identified from the SXSM samples using UPLC-Q-TOF-MS after removing duplicate values from the initially identified 278 components.

### Compound-related target searching

The study used three databases (Swiss Target Prediction, Stitch, and PharmMapper) to predict the action targets of 262 candidate compounds in SXSM. A total of 1680 action targets were collected, and 1295 unique compound action targets were identified after removing duplicates (**Supplementary Table 4**).

### Diseases-related target searching

To find targets related to myocardial ischemia and arrhythmia in humans, four databases (TTD, DisGeNET, OMIM, and GeneCards) were searched, resulting in 3077 targets. After removing duplicates, 1998 disease-related targets were identified.

### Core target screening

Protein interaction network maps were made for candidate compounds and disease targets using the STRING database. Those with a confidence score ≥ 0.95 were selected and merged using Cytoscape 3.9.1 software. Targets with a Degree value greater than the median were selected as core targets, resulting in 150 core targets (**Supplementary Table 5**).

### GO analysis and KEGG pathway analysis

Core targets were analyzed using DAVID (Version 6.8) for GO and KEGG pathway analysis. The analysis was performed with "official gene symbols" as identifiers, "gene list" as list type, restricted to Homo sapiens with FDR threshold of <0.05. The enrichment results were visualized.

## RNA-seq

### RNA extraction

RNA was extracted from iPSC-CMs using TRIzol® reagent (Invitrogen) and DNase I (TaKara) was used to remove genomic DNA. RNA quality and quantity were evaluated using a 2100 Bioanalyzer (Agilent) and an ND-2000 (NanoDrop Technologies). Samples meeting quality criteria (OD260/280=1.8~2.0, OD260/230≥2.0, RIN≥6.5, concentration≥100 ng/μl, total≥2μg) were used for library construction.

### Library building and sequencing

The paired-end library was constructed using the ABclonal mRNA-seq-Lib preparation kit (ABclonal, China). The mRNA was purified from 1 μg total RNA using oligo(dT) magnetic beads, followed by cleavage with divalent cations in ABclonal First Strand Synthesis Reaction Buffer. Subsequently, the first-strand cDNA was synthesized using the mRNA fragment as a template using random hexamer primers and reverse transcriptase (RNAseH), and the second-strand cDNA was then synthesized using DNA polymerase I, RNAseH, buffer, and dNTPs. The resulting double-stranded cDNA fragments were polyadenylated, ligated, and purified using the AMPure XP system (Beckman Coulter, Beverly, MA, USA). PCR amplification with adapter-ligated cDNA and adapter primers was then performed to prepare for sequencing on an Illumina Novaseq 6000 instrument.

### Transcriptomic data analysis

The Clean Reads are sequenced against the specified genome using HISAT2 software to obtain information on their position on the reference genome. Then the FPKM values (expected number of Fragments Per Kilobase of transcript sequence perMillions base pairs sequenced) of each gene expression in each sample were calculated using featureCounts software. Afterwards, differential expression analysis of genes was performed using Deseq2 software.

## 4D-Label-free quantitative proteomics

### Protein extraction and peptide enzymatic digestion

For sample lysis and protein extraction, SDT(4%(w/v) SDS, 100mM Tris/HCl pH7.6, 0.1M DTT) buffer was used. For protein quantification, BCA Protein Assay Kit (Bio-Rad, USA) was used. The filter-aided sample preparation (FASP) approach was followed for trypsin digestion of proteins^2^. The digest peptides of each sample were desalted on C18 Cartridges (Empore™ SPE Cartridges C18 (standard density), bed I.D. 7 mm, volume 3 ml, Sigma). The peptides were lyophilized and re-solubilized by adding 40 μL of 0.1% (v/v) formic acid solution and then quantified by OD280.

### LC-MS/MS analysis

A TimsTOF Pro mass spectrometer (Bruker) was coupled to a Nanoelute (Bruker Daltonics) for 60 min of LC-MS/MS analysis. The peptides were loaded onto a reversed-phase trap column (Thermo Scientific Acclaim PepMap100, 100 μm*2 cm, nanoViper C18) attached to a C18 reversed-phase analytical column (Thermo Scientific Easy Column, 10 cm long, 75 μm inner diameter, 3μm resin, C18-A2) in buffer A (0.1% Formic acid) and separated at 300 nl/min using a linear gradient of buffer B (84% acetonitrile and 0.1% Formic acid). Positive ion mode was used to operate the mass spectrometer. The mass spectrometer collected ion mobility MS spectra in the mass range of m/z 100-1700 and 1/k0 of 0.6 to 1.6, followed by 10 cycles of PASEF MS/MS with a target intensity of 1.5k and a threshold of 2500. With a release time of 0.4 minutes, active exclusion was initiated.

### Identification and quantitation of proteins

For identification and quantitation analysis, the MS raw data for each sample were combined and searched using the MaxQuant 1.6.14 software. Related parameters and instructions are as follows: Enzyme, Trypsin; Max Missed Cleavages, 2; Main search, 6ppm; First search, 20ppm; MS/MS Tolerance, 20ppm; Fixed modifications, Carbamidomethyl (C); Variable modifications, Oxidation (M); Database, Swissprot_Homo_sapiens_20395_20210106.fasta; Database pattern, Reverse; Include contaminants, True; Protein FDR, ≤0.01; Peptide FDR, ≤0.01; Peptides used for protein quantification, Use razor and unique peptides; Time window (match between runs), 2min; protein quantification, LFQ^3^; min. ratio count, 1.

## High-resolution untargeted metabolomics

### Sample Collection and Preparation of human iPSC-CM

The culture medium from the cultured human iPSC-CM cells (around 4*10^6^ cells per sample) was removed using pipette. The cells were then rinsed with PBS at 37°C. Remove the protein and extract all the metabolites from the sample using 800 μL of cold methanol/acetonitrile/water solution (2:2:1, v/v). The mixture was transferred to a fresh centrifuge tube, mixed by vertexing, sonicated at low temperature for 30 minutes, allowed to stand at -20°C for 10 minutes, and centrifuged at 14,000 g for 20 minutes at 4°C to obtain the supernatant. The supernatant was dried in a vacuum centrifuge. For LC-MS analysis, the samples were re-dissolved in 100 μL acetonitrile/water (1:1, v/v) solvent, mixed by vertexing, and centrifuged at 14,000 g for 15 minutes at 4°C to obtain the supernatant for analysis.

To ensure the consistency and repeatability of the instrumental analysis, quality control (QC) samples are prepared by combining 10 μL of each sample and analyzing it alongside other samples. The QC samples were routinely inserted and examined every 2 samples.

### LC-MS/MS Analysis

Analysis was conducted using an UHPLC (1290 Infinity LC, Agilent Technologies) coupled to a quadrupole time-of-flight (AB Sciex TripleTOF 6600).

UHPLC HILIC separation of samples was carried out using a 2.1 mm × 100 mm ACQUIY UPLC BEH 1.7 μm column (waters, Ireland): column Temperature 25°C; flow rate 0.5 mL/min; injection volume 2 μL. The mobile phase contained A (25 mM ammonium acetate and 25 mM ammonium hydroxide in water) and B (acetonitrile). The gradient elution procedure is as follows: 0 - 0.5 minutes, 95% B; 0.5 - 7 minutes, B varies linearly from 95% to 65 %; 7 - 8 min, B varied linearly from 65% to 40%; 8 - 9 min, B remained at 40%; 9 - 9.1 min, B varied linearly from 40% to 95%; 9.1 - 12 min, B maintained at 95%. Samples are placed in a 4°C autosampler during the entire analysis. To avoid the effects of fluctuations in the instrument's detection signal, a random order is used for continuous analysis of the samples.

The samples were separated by UHPLC and detected by mass spectrometer in electrospray ionization (ESI) positive and negative mode, respectively, for the acquisition of primary and secondary spectra of the samples.

The following were the ESI source conditions. Ion Source Gas1 (Gas1) as 60, Ion Source Gas2 (Gas2) as 60, curtain gas (CUR) as 30, source temperature: 600°C, IonSpray Voltage Floating (ISVF) ± 5500V. In MS only acquisition, the instrument was configured to acquire over the m/z range 60-1000 Da, and the accumulation time for TOF MS scan was set at 0.20s/spectra. In auto MS/MS acquisition, the instrument was set to acquire over the m/z range 25-1000 Da, and the accumulation time for product ion scan was set at 0.05s/spectra. The product ion scan is obtained with information-dependent acquisition (IDA) with the high sensitivity mode set. The parameters were set as follows: the collision energy (CE) was fixed at 35 V with ± 15 eV; decluttering potential (DP), 60 V (+) and -60 V (-); exclude isotopes within 4 Da, candidate ions to monitor per cycle: 10.

### Data processing

The metabolites in human iPSC-CMs were structurally characterized by high-resolution untargeted metabolomics using a UHPLC-Q-TOF MS/MS platform to further understand the pattern of metabolite changes induced in human iPSC-CM at 0.55mg/ml SXSM. Manual secondary verification is used to double-check the results, confirming that all detected metabolites have Level 2 or higher of Metabolomics Standards Initiative (MSI)^4^.

The raw MS data (wiff.scan files) were converted to MzXML files using ProteoWizard MSConvert before importing into freely available XCMS software. Parameters used for peak picking: centWave m/z = 10 ppm, peakwidth = c (10, 60), prefilter = c (10, 100). Parameters used for peak grouping: bw = 5, mzwid = 0.025, minfrac = 0.5. CAMERA (Collection of Algorithms for MEtabolite pRofile Annotation) was sued for isotope and adduct annotation. In the retrieved ion features, only the variables with more than 50% of the nonzero measurement values in at least one group were preserved. Metabolite identification was accomplished by comparing of accuracy m/z value (<10 ppm) and MS/MS spectra with an in-house database (Shanghai Applied Protein Technology) developed using accessible authentic standards^5,6^.

Metabolite annotations were graded using the Metabolomics Standards Initiative (MSI) confidence levels (levels 0-4)^4^. Level 0 indicates a well-defined 3D structure with stereochemical information. Level 1 requires reliable identification of a two-dimensional structure using at least two orthogonal properties (e.g., MS/MS spectra, retention time, or collision cross section values) compared to authentic chemical standards. Levels 2 and 3 are hypothetical annotations based on limited data, while level 4 indicates unknown compounds.

### OPLS-DA

The sum-normalized data were then subjected to multivariate data analysis using the R package (ropls), including an orthogonal partial least-squares discriminant analysis (OPLS-DA). OPLS-DA is a statistical analysis that uses supervised pattern recognition to identify DE metabolites by eliminating irrelevant effects. The scores were plotted for the CTRL and SXSM groups using OPLS-DA in both positive and negative modes. Both in positive and negative mode, CTRL and SXSM group significantly differentiated with each other in the predicted score for the first principal component (t [1]), and both samples were in the 95% confidence interval (Hotelling’s T2). The model's robustness was evaluated using 7-fold cross-validation and response permutation testing, with R^2^X representing the model's explanatory rate for X variables, R^2^Y for Y variables, and average predictability Q^2^ exceeding 0.5 in both comparison groups (Q^2^ in positive mode: 0.781, Q^2^ in negative mode: 0.516), indicating the model's suitability^7,8^. To avoid the OPLS-DA model from becoming overfit, 200 response permutation tests (RPTs) were undertaken. The replacement test plot of the comparison group OPLS-DA model shows that the R^2^ and Q^2^ of the stochastic model gradually decrease as the replacement retention gradually decreases, indicating the original model was not overfit and is robust^7-9^.

Each variable in the OPLS-DA model had its variable importance in the projection (VIP) value calculated to indicate its contribution to the classification. Student’s t test was applied to determine the significance of differences between two groups of independent samples. VIP > 1 and foldchange >1.2 or <1/1.2 were used to screen significant changed metabolites.

## Bioinformatic analysis in tri-omics analysis

### Enrichment Analysis

GSEA was performed on normalized RNA-Seq data of iPSC-CMs treated with SXSM utilizing the R package ("clusterProfiler," "org.Hs.eg.db," "enrichplot," and "ggplot2") with 1000 permutations^10^. We probed the KEGG, GO, and Reactome databases to identify the potential biological activities of SXSM on human iPSC-CMs using GSEA^11^. To be deemed statistically significant, enrichment observations must satisfy two conditions: a nominal p value 0.05 and |NES| > 1. KEGG pathway enrichment analyses were applied to explore the function of DE proteins. A *p*-value of < 0.05 was regarded as statistically significant. KEGG Pathways Enrichments were constructed using the R packages (“clusterProfiler,” “org.Hs.eg.db” “enrichplot”, and “ggplot2”). Additionally, metabolomic pathway analysis of DE metabolites in MetPA and MSEA were performed by MetaboAnalyst 5.0 (www.metaboanalyst.ca)^12^.

### Gene-metabolic O2PLS analysis

To merge transcriptomic and metabolomic data, an O2PLS analysis separated variation into three components: joint variation, orthogonal variation, and noise. The model used latent variables to explain variation^13^. The datasets were preprocessed, mean-centered, scaled, and normalized^14^. Only significant features from individual analyses were used in integration.

The OmicsPLS package in R was used to calculate O2PLS models^13^ with a permutation test for influential variable selection. The O2PLS model was produced for each rearranged dataset after 1000 rounds of data shuffle. The upper and lower α/2 quantiles of the loading values were utilized as thresholds for the latent variables with a significance level (α) of 0.05 for both datasets.

### Network analysis

With guidance from OmicsNet (https://www.omicsnet.ca/OmicsNet/)^15^, the multi-omics global interactome was constructed based on the InnateDB database of PPI and TRRUST database of TF-gene, and the topological characteristics of potential pathways/molecules were marked.

Cytoscape is a free, open-source platform designed specifically for visualizing and reconstructing networks^16^. The proteins in 14 pathways were selected from the top 20 KEGG-enriched pathways based on the function description of KEGG B-class classification. DE proteins from 14 KEGG pathways and all 29 DE metabolites from MSEA and MetPA analyses were imported into Cytoscape, and an interaction confidence level of 0.7 was used to retrieve the DE protein-DE metabolite interaction network using R software (including edge and node data). The network was loaded into Cytoscape 3.9.1 and portrayed with the "Prefuse Force Directed OpenCL Layout" weighted by -log10(p value).

## Western blot

To determine protein levels, standard Western-blot assays were used as previously discussed^17^. Phospho-CaMKII (CST, Thr286, 12716S) rabbit antibodies were prepared in our laboratory and the antibody dilutions for Western blotting is 1:1000.

# References

1. Fridericia, L.S. (2003). The duration of systole in an electrocardiogram in normal humans and in patients with heart disease. 1920. Ann Noninvasive Electrocardiol ***8***, 343-351, 10.1046/j.1542-474x.2003.08413.x.

2. Wiśniewski, J.R., Zougman, A., Nagaraj, N., and Mann, M. (2009). Universal sample preparation method for proteome analysis. Nat Methods ***6***, 359-362, 10.1038/nmeth.1322.

3. Cox, J., Hein, M.Y., Luber, C.A., et al. (2014). Accurate proteome-wide label-free quantification by delayed normalization and maximal peptide ratio extraction, termed MaxLFQ. Mol Cell Proteomics ***13***, 2513-2526, 10.1074/mcp.M113.031591.

4. Blaženović, I., Kind, T., Ji, J., and Fiehn, O. (2018). Software Tools and Approaches for Compound Identification of LC-MS/MS Data in Metabolomics. Metabolites ***8***, 10.3390/metabo8020031.

5. Luo, D., Deng, T., Yuan, W., et al. (2017). Plasma metabolomic study in Chinese patients with wet age-related macular degeneration. BMC Ophthalmol ***17***, 165, 10.1186/s12886-017-0555-7.

6. Gu, Z., Li, L., Tang, S., et al. (2018). Metabolomics Reveals that Crossbred Dairy Buffaloes Are More Thermotolerant than Holstein Cows under Chronic Heat Stress. J Agric Food Chem ***66***, 12889-12897, 10.1021/acs.jafc.8b02862.

7. Triba, M.N., Le Moyec, L., Amathieu, R., et al. (2015). PLS/OPLS models in metabolomics: the impact of permutation of dataset rows on the K-fold cross-validation quality parameters. Mol Biosyst ***11***, 13-19, 10.1039/c4mb00414k.

8. Wheelock Å, M., and Wheelock, C.E. (2013). Trials and tribulations of 'omics data analysis: assessing quality of SIMCA-based multivariate models using examples from pulmonary medicine. Mol Biosyst ***9***, 2589-2596, 10.1039/c3mb70194h.

9. Westerhuis, J.A., Hoefsloot, H.C., Smit, S., et al. (2008). Assessment of PLSDA cross validation. Metabolomics ***4***, 81-89.

10. Gao, J., Aksoy, B.A., Dogrusoz, U., et al. (2013). Integrative analysis of complex cancer genomics and clinical profiles using the cBioPortal. Sci Signal ***6***, pl1, 10.1126/scisignal.2004088.

11. Subramanian, A., Tamayo, P., Mootha, V.K., et al. (2005). Gene set enrichment analysis: a knowledge-based approach for interpreting genome-wide expression profiles. Proc Natl Acad Sci U S A ***102***, 15545-15550, 10.1073/pnas.0506580102.

12. Pang, Z., Zhou, G., Ewald, J., et al. (2022). Using MetaboAnalyst 5.0 for LC-HRMS spectra processing, multi-omics integration and covariate adjustment of global metabolomics data. Nat Protoc ***17***, 1735-1761, 10.1038/s41596-022-00710-w.

13. Bouhaddani, S.E., Houwing-Duistermaat, J., Salo, P., et al. (2016). Evaluation of O2PLS in Omics data integration. BMC Bioinformatics ***17 Suppl 2***, 11, 10.1186/s12859-015-0854-z.

14. Bylesjö, M., Eriksson, D., Kusano, M., et al. (2007). Data integration in plant biology: the O2PLS method for combined modeling of transcript and metabolite data. Plant J ***52***, 1181-1191, 10.1111/j.1365-313X.2007.03293.x.

15. Zhou, G., Pang, Z., Lu, Y., et al. (2022). OmicsNet 2.0: a web-based platform for multi-omics integration and network visual analytics. Nucleic Acids Res ***50***, W527-533, 10.1093/nar/gkac376.

16. Shannon, P., Markiel, A., Ozier, O., et al. (2003). Cytoscape: a software environment for integrated models of biomolecular interaction networks. Genome Res ***13***, 2498-2504, 10.1101/gr.1239303.

17. Li, P., Guo, Z., Feng, R., et al. (2022). Multi-omics analysis reveals the regulation of SIRT6 on protein processing of endoplasmic reticulum to alleviate oxidative stress in endothelial cells. Clin Transl Med ***12***, e1039, 10.1002/ctm2.1039.
