# Supplementary material for: Transcriptomics, proteomics, metabolomics and network pharmacology reveal molecular mechanisms of multi‐targets effects of Shenxianshengmai improving human iPSC‐CMs beating
Source: Clin Transl Med. 2023 Jun 6;13(6):e1302. doi: 10.1002/ctm2.1302 (PMC10246690; doi:10.1002/ctm2.1302)
Supplement: Supplementary file 4 — Table S1‐S6 [file CTM2-13-e1302-s008.docx]

# Supplementary Tables 1-6

# Supplementary Table 1. Identification of major compounds in Shenxian-Shengmai (SXSM) by LC-MS

| No. | RT  （min） | Adduct ions | Measured m/z | Respected m/z | ppm | Formula | M.W. | Identification | MS/MS data | Source |
| --- | --- | --- | --- | --- | --- | --- | --- | --- | --- | --- |
| 001 | 2.41 | M^+^ | 104.1069 | 104.107 | -0.9 | C_5_H_14_NO+ | 104.11 | Choline | 104.1055;30.0792 | GQZ |
| 002* | 2.63 | [M+H]^+^ | 118.086 | 118.0863 | -2.2 | C_5_H_11_NO_2_ | 117.08 | Betaine | 118.0864;58.0647 | GQZ |
| 003 | 2.63 | [M-H]^-^ | 195.0508 | 195.051 | -1.2 | C_6_H_12_O_7_ | 196.06 | Gluconic acid | 195.0500;129.0194;75.0083 | / |
| 004 | 2.66 | [M-H]^-^ | 294.0826 | 294.0831 | -1.5 | C_10_H_17_NO_9_ | 295.09 | L-Aspartic acid,4-β-D-glucopyranosyl ester | 132.0292;108.0448;88.0402 | GQZ |
| 005 | 2.99 | [M-H]^-^ | 191.0559 | 191.0561 | -1.1 | C_7_H_12_O_6_ | 192.06 | Quinic acid | 191.0556;171.0294;127.0383;85.0298 | GQZ |
| 006 | 5.33 | [M-H]^-^ | 273.0736 | 273.0768 | -11.9 | C_15_H_14_O_5_ | 274.08 | Methysticin | 147.0285;110.0239;82.0293 | / |
| 007 | 6.46 | [M-H]^-^ | 337.0774 | 337.0776 | -0.7 | C_12_H_18_O_11_ | 338.08 | L-Ascorbic acid 2-glucoside | 337.0768;277.0566;174.0147;101.0241 | GQZ |
| 008 | 6.58 | [M+H]^+^ | 130.0499 | 130.0499 | 4.1 | C_5_H_7_NO_3_ | 129.04 | L-Pyroglutamic acid | 130.0491;84.0443;56.0493 | GQZ |
| 009 | 8.07 | [M+H]^+^ | 132.102 | 132.1019 | 0.7 | C_6_H_13_NO_2_ | 131.09 | Leucine | 86.0957 | GQZ |
| 010 | 8.79 | [M+H]^+^ | 132.102 | 132.1019 | 0.7 | C_6_H_13_NO_2_ | 131.09 | 6-Deoxyfagomine | 86.0955;72.9369;69.0701 | GQZ |
| 011 | 9.32 | [M+H]^+^ | 330.0589 | 330.0608 | -5.8 | C_16_H_11_NO_7_ | 329.05 | 5,7-Dihydroxy-6-methoxy-2-(4-nitrophenyl) chromen-4-one | 330.0579;136.0616;119.0357 | / |
| 012 | 9.68 | [M-H]^-^ | 180.0675 | 180.0666 | 4.9 | C_9_H_11_NO_3_ | 181.07 | L-Tyrosine | 180.0644;163.0371;119.0504 | GQZ |
| 013* | 9.83 | [M-H]^-^ | 243.0616 | 243.0623 | -2.7 | C_9_H_12_N_2_O_6_ | 244.07 | Uridine | 243.0649;200.0595;152.0344;122.0235;110.0250 | All |
| 014 | 10.41 | [M-H]^-^ | 282.0846 | 282.0831 | 5.5 | C_9_H_17_NO_9_ | 283.09 | L-Serine, N-D-gluconoyl- (9CI) | 282.0848;150.0414;133.0146;108.0205 | / |
| 015 | 10.41 | [M+H]^+^ | 294.1563 | 294.1547 | 5.3 | C_12_H_23_NO_7_ | 293.15 | N-(1-Deoxy-1-fructosyl) leucine | 276.1470;258.1375;230.1413;212.1310 | / |
| 016* | 11.56 | [M+H]^+^ | 166.1222 | 166.1226 | -2.7 | C_10_H_15_NO | 165.12 | Hordenine | 121.0643;103.0536;91.0539;77.0373 | MH |
| 017 | 12.68 | [M+H]^+^ | 166.0858 | 166.0863 | -2.7 | C_9_H_11_NO_2_ | 165.08 | Phenylalanine | 120.0805;103.0540;91.0540 | GQZ |
| 018 | 12.91 | [M-H]^-^ | 309.11 | 309.1092 | 2.6 | C_14_H_18_N_2_O_6_ | 310.12 | L-γ-Glutamyl-L-tyrosine | 180.0672;163.0396;128.0349;106.0417 | / |
| 019 | 13.2 | [M-H]^-^ | 241.0828 | 241.083 | -0.8 | C_10_H_14_N_2_O_5_ | 242.09 | Thymidine | / | / |
| 020 | 13.3 | [M-H]^-^ | 471.1606 | 471.162 | -3 | C_20_H_28_N_2_O_11_ | 472.17 | Propanoic acid, 3-[[3-[[2-[3-(β-D-glucopyranosyloxy)-4-hydroxyphenyl] ethyl]amino]-3-oxopropyl]amino]-3-oxo- (9CI) | 471.1566;381.1349;309.1125;291.0941;180.0666;167.0447;128.0350 | / |
| 021 | 13.88 | [M+H]^-^ | 311.1236 | 311.1238 | -0.5 | C_14_H_18_N_2_O_6_ | 310.12 | Tyrosylglutamic acid | 294.0945;165.0555;147.0445;136.0764 | / |
| 022 | 13.9 | [M-H]^-^ | 515.1404 | 515.1406 | -0.4 | C_22_H_28_O_14_ | 516.15 | 3-O-(3'-O-caffeoyl glucosyl) quinic acid | 515.1412;341.0889;191.0573;179.0349;135.0446 | GQZ |
| 023 | 14.08 | [M-H]^-^ | 315.072 | 315.0722 | -0.5 | C_13_H_16_O_9_ | 316.08 | Protocatechuic acid 3-O-glucoside | 315.0731;152.0114;108.0211 | GQZ |
| 024 | 14.21 | [M-H]^-^ | 431.1192 | 431.1195 | -0.7 | C_18_H_24_O_12_ | 432.13 | 6-O-D-Apio-beta-D-furanosyl-beta-D-glucopyranose 1-(4-hydroxybenzoate) | 431.1200;299.0774;137.0240 | / |
| 025* | 14.22 | [2M-H]^-^ | 395.0984 | 395.0984 | 0.1 | C_9_H_10_O_5_ | 198.05 | Danshensu | 197.0450;179.0351;135.0441;123.0444 | DS |
| 026 | 14.23 | [M-H]^-^ | 417.0802 | 417.0827 | -6 | C_20_H_18_O_10_ | 418.09 | Salvianolic acid D | 417.0788;219.0273;197.0447;173.0204;150.0317;135.0426 | DS |
| 027 | 14.3 | [M-H]^-^ | 329.0889 | 329.0878 | 3.3 | C_14_H_18_O_9_ | 330.1 | Pseudolaroside B | 167.0351;152.0105;123.0452;108.0214 | MH |
| 028 | 14.61 | [M-H]^-^ | 461.1296 | 461.1301 | -1 | C_19_H_26_O_13_ | 462.14 | Saccharumoside C | 461.1296;167.0343;152.0112;108.0215 | BGZ |
| 029 | 14.83 | [M-H]^-^ | 315.0734 | 315.0722 | 3.9 | C_13_H_16_O_9_ | 316.08 | Protocatechuic acid-3-O-glucoside Isomer | 315.0706;153.0189;109.0295 | GQZ |
| 030 | 15 | [M-H]^-^ | 515.1427 | 515.1406 | 4.0 | C_22_H_28_O_14_ | 516.15 | cis 5-O-(3'-O-caffeoyl glucosyl) quinic acid | 515.1415;353.0862;341.0931;191.0562;179.0375 | GQZ |
| 031 | 15.06 | [M+H]^+^ | 206.0448 | 206.0446 | 0.1 | C_10_H_7_NO_4_ | 205.04 | 6-Hydroxykynurenic acid | 188.0354;160.0381 | MH |
| 032 | 15.16 | [M-H]^-^ | 153.019 | 153.0193 | -2.2 | C_7_H_6_O_4_ | 154.03 | Protocatechuic acid | 153.0185;109.0298;91.0183 | DS |
| 033 | 15.31 | [M+H]^+^ | 166.123 | 166.1226 | 2.2 | C_10_H_15_NO | 165.12 | Ephedrine | 148.117;132.0800;115.0537;91.0534 | MH |
| 034 | 15.36 | [M-H]^-^ | 515.1388 | 515.1406 | -3.6 | C_22_H_28_O_14_ | 516.15 | 4-O-(3'-O-caffeoyl glucosyl) quinic acid | 515.1422;353.0885;341.0855;191.0553;179.0345 | GQZ |
| 035 | 15.7 | [M+FA-H]^-^ | 549.182 | 549.1825 | -0.9 | C_22_H_32_O_13_ | 504.18 | Coniferinoside | 549.1865;503.1809;341.1237;179.0548;161.0443 | / |
| 036* | 15.97 | [M-H]^-^ | 353.0878 | 353.0878 | 0 | C_16_H_18_O_9_ | 354.1 | Neochlorogenic acid | 353.0861;191.0561;179.0346;135.0454 | MH |
| 037 | 16.03 | [M+H]^+^ | 265.1545 | 265.1547 | -0.6 | C_14_H_20_N_2_O_3_ | 264.15 | trans-N-Feruloylputrescine | 248.1329;177.0559;145.0293 | GQZ |
| 038 | 16.1 | [M-H]^-^ | 515.1427 | 515.1406 | -1 | C_22_H_28_O_14_ | 516.15 | tran 5-O-(3'-O-caffeoyl glucosyl) quinic acid | 515.1412;353.0928;341.0852;179.0351;173.0455 | GQZ |
| 039 | 16.21 | [M+H]^+^ | 493.2821 | 493.2809 | 2.4 | C_28_H_36_N_4_O_4_ | 492.27 | Ephedradine A | / | MH |
| 040* | 16.22 | [M-H]^-^ | 353.086 | 353.0878 | -5.1 | C_16_H_18_O_9_ | 354.1 | Chlorogenic acid | 353.0875;191.0551;173.0452;135.0448 | MH |
| 041 | 16.33 | [M+FA-H]^-^ | 579.1943 | 579.1931 | 2.1 | C_23_H_34_O_14_ | 534.19 | Glucopyranosyloxy)-3,5-dimethoxyphenyl]-2-propen-1-yl beta-D-glucopyranoside | 579.2074;533.1935;371.1345;179.0549;161.0458 | / |
| 042 | 16.37 | [M-H]^-^ | 203.0825 | 203.0826 | -0.5 | C_11_H_12_N_2_O_2_ | 204.09 | L-Tryptophan | 203.0835;116.0508 | GQZ |
| 043 | 16.39 | [M+H]^+^ | 180.1389 | 180.1383 | 3.4 | C_11_H_17_NO | 179.13 | N-Methylephedrine | 180.1370;162.1269;115.0537;91.0532 | MH |
| 044 | 16.49 | [M+H]^+^ | 224.1292 | 224.1281 | 4.8 | C_12_H_17_NO_3_ | 223.12 | Maokonine | 224.1638;206.1174;147.1041 | MH |
| 045 | 16.67 | [M+FA-H]^-^ | 535.2059 | 535.2032 | 5 | C_22_H_34_O_12_ | 490.21 | bis-2-methyl-5-(1-methylethyl)-1,4-phenylene-β-D-Glucopyranoside | 489.2035;327.1420;165.0931 | DS |
| 046 | 16.91 | [M-H]^-^ | 487.1465 | 487.1457 | 1.6 | C_21_H_28_O_13_ | 488.15 | p-Hydroxycinnamic sophorose | 487.1425;163.0396;145.0276;119.0493 | DS |
| 047* | 16.92 | [M-H]^-^ | 487.1447 | 487.1457 | -2.1 | C_21_H_28_O_13_ | 488.15 | Cistanoside F | 487.1426;187.0408;163.0387;119.0531 | DS |
| 048* | 17.2 | [M-H]^-^ | 577.1563 | 577.1563 | 0.0 | C_27_H_30_O_14_ | 578.16 | Vitexin-2-O-rhamnoside | 577.1576;457.1135;415.1040;295.0603;267.0659 | YYH |
| 049 | 17.25 | [M-H]^-^ | 515.1427 | 515.1406 | 4.0 | C_22_H_28_O_14_ | 516.15 | cis 1-O-(3'-O-caffeoyl glucosyl) quinic acid | 515.1377;353.0877;341.0812;323.0776;191.0583;179.0354 | GQZ |
| 050 | 17.74 | [M+H]^+^ | 190.0492 | 190.0499 | -3.5 | C_10_H_7_NO_3_ | 189.04 | Kynurenic acid | 172.0458;144.0438;116.0480 | MH |
| 051 | 17.81 | [M+FA-H]^-^ | 623.1625 | 623.1618 | 1.2 | C_27_H_30_O_14_ | 578.16 | Daidzein-4,'7-diglucoside | 623.1585;415.1051;252.0574 | BGZ |
| 052 | 17.88 | [M+H]^+^ | 265.1556 | 265.1547 | 3.5 | C_14_H_20_N_2_O_3_ | 264.15 | Feruloylputrescine | 177.0565;145.0305 | GQZ |
| 053 | 17.96 | [M+H]^+^ | 636.3119 | 636.3127 | -1.2 | C_31_H_45_N_3_O_11_ | 635.31 | Lycibarbarspermindine I | 636.3219;474.2631;384.169;236.1356;222.1165 | GQZ |
| 054* | 18.08 | [M-H]^-^ | 137.0251 | 137.0244 | 5 | C_7_H_6_O_3_ | 138.03 | Protocatechualdehyde | 137.0246;119.0132;108.0213;92.0265 | DS |
| 055 | 18.25 | [M-H]^-^ | 515.1406 | 515.1406 | -0.1 | C_22_H_28_O_14_ | 516.15 | tran 1-O-(3'-O-caffeoyl glucosyl) quinic acid | 515.1372;353.0954;341.0866;323.0731;191.0565;179.0355 | GQZ |
| 056 | 18.29 | [M+H]^+^ | 288.1348 | 288.1343 | 1.8 | C_15_H_17_N_3_O_3_ | 287.13 | Feruloylhistamine | 177.0549;145.0279;141.1016;112.0874 | MH |
| 057 | 18.47 | [M-H]^-^ | 337.0916 | 337.0929 | -3.8 | C_16_H_18_O_8_ | 338.1 | 5-p-coumaroylquinic acid | 337.0905;191.0560;173.0481;163.0400 | GQZ |
| 058 | 18.8 | [M-H]^-^ | 433.1153 | 433.114 | 3 | C_21_H_22_O_10_ | 434.12 | Naringenin-6-C-glucoside | 433.1138;343.0818;313.0721 | / |
| 059 | 18.89 | [M+H]^+^ | 634.3017 | 634.297 | 7.4 | C_31_H_43_N_3_O_11_ | 633.29 | Lycibarbarspermindine A | 634.3090;472.2498;220.0999 | GQZ |
| 060 | 19.04 | [M-H]^-^ | 325.0936 | 325.0929 | 2.2 | C_15_H_18_O_8_ | 326.1 | Melilotoside | 163.0388;119.0495 | GQZ |
| 061 | 19.09 | M^+^ | 773.2161 | 773.2135 | 3.4 | C_33_H_41_O_21_+ | 773.21 | Delphinidin-3-O-rutinoside-5-O-glucoside | 773.2206;627.1675L465.1069;303.0534 | GQZ |
| 062 | 19.1 | [M-H]^-^ | 353.0862 | 353.0878 | -4.5 | C_16_H_18_O_9_ | 354.1 | Cryptochlorogenic acid | 353.0943;191.0562;173.0439 | MH |
| 063 | 19.17 | [M-H]^-^ | 771.2003 | 771.1989 | 1.8 | C_33_H_40_O_21_ | 772.21 | Quercetin 3-O-glucosyl-rutinoside | 771.1948;725.2466;609.1462;462.0763 | / |
| 064 | 19.52 | [M+H]^+^ | 935.2637 | 935.2663 | -2.8 | C_39_H_50_O_26_ | 934.26 | Quercetin-3-O-sophorotrioside-7-O-rhamnoside | 935.2860;789.2242;627.1644;465.1131;3030.0528 | / |
| 065 | 19.89 | [M+H]^+^ | 474.2601 | 474.2599 | 0.5 | C_25_H_35_N_3_O_6_ | 473.25 | N1, N8-bis-dihydrocaffeoyl-spermidine | 474.2601;236.1303;222.1145;165.0563;123.0442 | GQZ |
| 066 | 19.92 | [M+FA-H]^-^ | 373.1499 | 373.1504 | -1.4 | C_16_H_24_O_7_ | 328.15 | Rhododendrin | 165.0893 | MH |
| 067 | 19.98 | [M-H]^-^ | 457.1365 | 457.1352 | 3 | C_20_H_26_O_12_ | 458.14 | p-Coumaric acid 4-[apiosyl-(1->2)-glucoside] | 457.1334;163.0377;119.0498 | / |
| 068 | 19.99 | [M+H]^+^ | 472.2457 | 472.2442 | 3.1 | C_25_H_33_N_3_O_6_ | 471.24 | N1-Hydrocaffeoyl-N8-caffeoylspermidine | 472.2502;310.2153;222.1146;220.0996;163.0404 | GQZ |
| 069 | 20.77 | [M-H]^-^ | 177.0185 | 177.0193 | -4.7 | C_9_H_6_O_4_ | 178.03 | Esculetin | 177.0203;149.0204;133.0290;105.0374 | GQZ |
| 070 | 20.77 | M^+^ | 611.163 | 611.1607 | 3.8 | C_27_H_31_O_16_+ | 611.16 | Cyanidin 3,5-di-O-glucoside | 611.1860;449.1032;287.0552 | GQZ |
| 071 | 20.98 | [M+FA-H]^-^ | 371.1331 | 371.1348 | -4.5 | C_16_H_22_O_7_ | 326.14 | Eugenyl glucoside | 163.0756 | / |
| 072 | 21.01 | [M+H]^+^ | 472.2452 | 472.2442 | 2.1 | C_25_H_33_N_3_O_6_ | 471.24 | N1-Caffeoyl-N8-hydrocaffeoylspermidine | 472.2502;310.2166;293.1870;220.0979;163.0388 | GQZ |
| 073* | 21.24 | [M-H]^-^ | 179.0352 | 179.035 | 1.2 | C_9_H_8_O_4_ | 180.04 | caffeic acid | 179.0353;135.0447 | GQZ/DS |
| 074 | 21.35 | [M+FA-H]^-^ | 431.1917 | 431.1923 | -1.3 | C_19_H_30_O_8_ | 386.19 | Icariside B1 | 385.1862;223.1323;153.0921 | YYH |
| 075 | 21.43 | [M+FA-H]^-^ | 433.208 | 433.2079 | 0.2 | C_19_H_32_O_8_ | 388.21 | Icariside B5 | 387.1987;225.1494;179.0569 | YYH |
| 076* | 21.5 | M^+^ | 449.1081 | 449.1078 | 0.6 | C_21_H_21_O_11_^+^ | 449.11 | Cyanidin-3-O-glucoside | 287.0539 | GQZ |
| 077 | 21.68 | [M+H]^+^ | 220.0607 | 220.0604 | 1.2 | C_11_H_9_NO_4_ | 219.05 | 6-methoxykynurenic acid | 202.0508;174.0545;146.0605;119.0485 | MH |
| 078* | 21.75 | [M-H]^-^ | 415.1015 | 415.1035 | -4.7 | C_21_H_20_O_9_ | 416.11 | Puerarin | 415.1045;295.0607;267.0655 | MH |
| 079* | 21.8 | [M-H]^-^ | 593.1503 | 593.1512 | -1.5 | C_27_H_30_O_15_ | 548.15 | Vicenin-2 | 593.1493;503.1197;473.1077;383.0731;353.0643 | MH |
| 080* | 21.93 | M^+^ | 342.1714 | 342.17 | 4.1 | C_20_H_24_NO_4_ | 342.17 | Magnoflorine | 342.1704;299.1254;297.1138;265.0878;247.0770;222.0680 | YYH |
| 081 | 22.3 | [M-H]^-^ | 579.1373 | 579.1355 | 3 | C_26_H_28_O_15_ | 580.14 | Carlinoside | 579.1344;519.1011;489.1036;429.0827;399.0680;369.0560;339.0371 | / |
| 082 | 22.34 | [M-H]^-^ | 121.0303 | 121.0295 | 6.6 | C_7_H_6_O_2_ | 122.04 | Salicylaldehyde | 121.0289;92.0279 | / |
| 083 | 22.42 | [M-H]^-^ | 563.1403 | 563.1406 | -0.6 | C_26_H_28_O_14_ | 564.15 | Vicenin-1 | 563.1389;503.1138;473.1102;443.0922;383.0754;353.0636 | MH |
| 084 | 22.59 | [M-H]^-^ | 337.0934 | 337.0929 | 1.5 | C_16_H_18_O_8_ | 338.1 | 3-p-coumaroylquinic acid | 337.0942;191.0557;173.0458 | GQZ |
| 085 | 22.91 | [M-H]^-^ | 593.1522 | 593.1512 | 1.7 | C_27_H_30_O_15_ | 594.16 | Kaempferol-3-O-rutinoside | 593.1521;447.1017;431.1010;285.0351 | / |
| 086 | 23.33 | [M-H]^-^ | 563.14 | 563.1406 | -1.1 | C_26_H_28_O_14_ | 564.15 | Isoschaftoside | 563.1394;503.1323;473.1217;443.1000;383.0758;353.0636 | MH |
| 087 | 23.34 | [M+FA-H]^-^ | 373.1515 | 373.1517 | 2.9 | C_16_H_24_O_7_ | 328.15 | Rhododendrin isomer | 327.1432;165.0922 | MH |
| 088* | 23.49 | [2M-H]^-^ | 731.1839 | 731.1829 | 1.4 | C_17_H_18_O_9_ | 366.1 | Psoralenoside | 731.1864;365.0867;203.0338;159.0447 | BGZ |
| 089 | 23.53 | [M-H]^-^ | 325.0919 | 325.0929 | -3 | C_15_H_18_O_8_ | 326.1 | 6-O-p-Coumaroyl-D-glucose | 163.0403;119.0501 | GQZ |
| 090 | 23.99 | [M-H]^-^ | 563.1418 | 563.1406 | 2.1 | C_26_H_28_O_14_ | 564.15 | Schaftoside | / | MH |
| 091 | 24.15 | [M+FA-H]^-^ | 787.2653 | 787.2666 | -1.7 | C_34_H_46_O_18_ | 742.27 | Liriodendrin | 787.2670;741.2568;579.2061;417.1551 | / |
| 092* | 24.23 | [2M-H]^-^ | 731.1816 | 731.1829 | -1.8 | C_17_H_18_O_9_ | 366.1 | Isopsoralenoside | 731.1849;365.0858;203.0350;159.0451 | BGZ |
| 093 | 24.34 | [M-H]^-^ | 447.0905 | 447.0933 | -6.2 | C_21_H_20_O_11_ | 448.1 | Isoorientin | 447.0915;357.0615;327.0494 | / |
| 094 | 24.47 | [M-H]^-^ | 431.1009 | 431.0984 | 5.9 | C_21_H_20_O_10_ | 432.11 | Isovitexin | 431.1084;283.0492;249.0640;181.0324 | / |
| 095 | 24.78 | [M-H]^-^ | 739.2067 | 739.2091 | -3.3 | C_33_H_40_O_19_ | 740.22 | Kaempferol 3-O-rutinoside-7-O-rhamnoside | 739.2136;593.1466;430.0925;285.0389;284.0295 | YYH |
| 096 | 25.09 | [M-H]^-^ | 523.2179 | 523.2185 | -1.1 | C_26_H_36_O_11_ | 524.23 | Icariside E3 | 523.2193;361.1626;165.0513 | YYH |
| 097 | 25.12 | [M-H]^-^ | 479.0817 | 479.0831 | -3 | C_21_H_20_O_13_ | 480.09 | Myricetin 3-galactoside | 479.0827;316.0198;287.0169;271.0185 | / |
| 098* | 25.24 | [M-H]^-^ | 563.1413 | 563.1406 | 1.2 | C_26_H_28_O_14_ | 564.15 | Vicenin-3 | 563.1394;473.1099;443.0995;413.0919;383.0659;353.0673 | MH |
| 099* | 25.87 | [M-H]^-^ | 163.0406 | 163.0401 | 3.3 | C_9_H_8_O_3_ | 164.05 | p-coumaric acid | 163.0404;119.0505 | GQZ |
| 100 | 25.99 | [M-H]^-^ | 593.1534 | 593.1512 | 3.7 | C_27_H_30_O_15_ | 594.16 | Quercetin 3,7-di-O-rhamnoside | 593.1474;447.0903;301.0346 | / |
| 101 | 26.02 | [M-H]^-^ | 561.1644 | 561.1672 | -5.1 | C_27_H_30_O_13_ | 562.17 | Chrysin 7-rutinoside | 561.1615;253.0509 | DS |
| 102 | 26.67 | [M-H]^-^ | 577.159 | 577.1563 | 4.7 | C_27_H_30_O_14_ | 578.16 | Vitexin-2-O-rhamnoside Isomer | 577.1583;457.1146;413.0864;293.0446 | / |
| 103 | 26.85 | M^+^ | 611.1608 | 611.1607 | 0.2 | C_27_H_31_O_16_+ | 611.16 | Delphinidin 3-O-rutinoside | 611.1840;3030.05112 | GQZ |
| 104 | 26.92 | [M-H]^-^ | 739.2115 | 739.2091 | 3.2 | C_33_H_40_O_19_ | 740.22 | Kaempferol-3-O-rhamnopyranosyl-glucopyranosyl-7-O-rhamnopyranoside Isomer | 739.2060;593.1489;430.0891;285.0418;284.0313 | YYH |
| 105* | 26.99 | [M-H]^-^ | 609.1462 | 609.1461 | 0.1 | C_27_H_30_O_16_ | 610.15 | Rutin | 609.1424;447.0862;300.0272;299.0147 | MH |
| 106 | 27.17 | [M-H]^-^ | 191.036 | 191.035 | 5.3 | C_10_H_8_O_4_ | 192.04 | Scopoletin | 191.0332;176.0114;148.0186;120.0194 | GQZ |
| 107 | 27.54 | [M-H]^-^ | 537.1009 | 537.1039 | -5.5 | C_27_H_22_O_12_ | 538.11 | Salvianolic acid I | 537.0987;493.1135;313.0715;295.0595 | DS |
| 108 | 28.09 | M^+^ | 465.1021 | 465.1028 | -1.4 | C_21_H_21_O_12_+ | 465.1 | Delphinidin-3-O-glucoside | 303.0499;229.0502 | GQZ |
| 109 | 28.11 | [M-H]^-^ | 463.0867 | 463.0882 | -3.2 | C_21_H_20_O_12_ | 464.1 | Quercetin-3-O-galactoside | 463.0860;300.0245;271.0234 | MH |
| 110* | 28.25 | [M-H]^-^ | 577.1576 | 577.1563 | 2.3 | C_27_H_30_O_14_ | 578.16 | Kaempferitrin | 577.1624;431.0982;285.0405 | MH |
| 111 | 28.35 | [M-H]^-^ | 537.104 | 537.1039 | 0.3 | C_27_H_22_O_12_ | 538.11 | Salvianolic acid H | 537.1015;493.1120;313.0707;295.0601 | DS |
| 112 | 28.74 | [M-H]^-^ | 521.127 | 521.1301 | -5.9 | C_24_H_26_O_13_ | 522.14 | Salviaflaside | 521.1266;359.0745;323.0768;161.0240 | DS |
| 113 | 28.86 | [M-H]^-^ | 431.0994 | 431.0984 | 2.4 | C_21_H_20_O_10_ | 432.11 | Apigenin 7-glucoside | 431.0986;269.0449 | / |
| 114 | 29.26 | M^+^ | 479.1183 | 479.1028 | -6.6 | C_22_H_23_O_12_+ | 479.12 | Petunidin-3-glucoside | 317.0663;302.0401 | GQZ |
| 115 | 29.49 | [M-H]^-^ | 477.1046 | 477.1039 | 1.6 | C_22_H_22_O_12_ | 478.11 | Isorhamnetin-3-O-glucoside | 477.1044;314.0392;299.0164;271.0224;255.0287 | / |
| 116 | 29.5 | [M-H]^-^ | 593.1492 | 593.1512 | -3.4 | C_27_H_30_O_15_ | 594.16 | kaempferol 3-O-beta-D-glucopyranosyl-7-O-alpha-L-rhamnopyranoside | 593.1470;285.0371 | / |
| 117 | 29.92 | [M-H]^-^ | 579.2079 | 579.2083 | -0.7 | C_28_H_36_O_13_ | 580.22 | (+)-syringaresinol beta-D-glucoside | 417.1566;402.1317;387.1006;181.0498 | / |
| 118 | 30.18 | M^+^ | 465.104 | 465.1028 | 2.7 | C_21_H_21_O_12_+ | 465.1 | Delphinidin 3-galactoside | 303.0507 | GQZ |
| 119 | 30.26 | [M-H]^-^ | 463.0859 | 463.0882 | -5 | C_21_H_20_O_12_ | 464.1 | Isoquercitrin | 463.0844;301.0336;300.0214;255.0312 | MH |
| 120 | 30.57 | [M-H]^-^ | 417.082 | 417.0827 | -1.7 | C_20_H_18_O_10_ | 418.09 | Salvianolic acid D isomer | 417.0758;373.0945;197.0455;175.0394;157.0279;135.0452 | DS |
| 121 | 30.76 | [M-H]^-^ | 715.1295 | 715.1305 | -1.3 | C_36_H_28_O_16_ | 716.14 | 7''',8'''-Didehydro-salcianolic acid B | 715.1253;519.0985;321.0412 | DS |
| 122 | 30.94 | [M-H]^-^ | 447.0913 | 447.0933 | -4.4 | C_21_H_20_O_11_ | 448.1 | Kaempferol 7-O-glucoside | 447.0939;285.0386;255.0279;227.0334 | / |
| 123 | 31.23 | [M-H]^-^ | 677.2125 | 677.2087 | 5.6 | C_32_H_38_O_16_ | 678.22 | CAS:1428239-91-1 | 677.2093;530.1348;369.0958 | YYH |
| 124 | 31.74 | [M-H]^-^ | 879.1974 | 879.1989 | -1.7 | C_42_H_40_O_21_ | 880.21 | Salvinoside | 879.1959;681.1438;519.0884;321.0390 | DS |
| 125 | 31.94 | [M-H]^-^ | 677.2096 | 677.2087 | 1.3 | C_32_H_38_O_16_ | 678.22 | Hexandraside E | 677.2082;515.1548;353.0988 | YYH |
| 126 | 32.25 | M^+^ | 493.1336 | 493.1341 | -0.9 | C_23_H_25_O_12_^+^ | 493.13 | Malvidin-3-O-glucoside | 331.0814;315.0470 | GQZ |
| 127* | 32.35 | [M-H]^-^ | 717.1455 | 717.1461 | -0.8 | C_36_H_30_O_16_ | 718.15 | Salvianolic acid E | 717.1496;519.0908;339.0469;321.0379;295.0597 | DS |
| 128 | 32.35 | M^+^ | 463.1234 | 463.1235 | -0.2 | C_22_H_23_O_11_^+^ | 463.12 | Malvidin-3-O-arabinoside | 301.0712;286.0469 | GQZ |
| 129 | 32.58 | [M-H]^-^ | 823.2689 | 823.2666 | 2.8 | C_38_H_48_O_20_ | 824.27 | Ikarisoside C | 823.2661;661.2123;515.159+1;353.1005 | YYH |
| 130 | 32.74 | [M+FA-H]^-^ | 1007.549 | 1007.5432 | 5.7 | C_48_H_82_O_19_ | 962.55 | 20-Glucoginsenoside Rf | 1007.5519;961.5386;799.4971;637.4398 | HS |
| 131* | 33.11 | [M+H]^+^ | 163.0392 | 163.039 | 1.4 | C_9_H_6_O_3_ | 162.03 | Umbelliferone | 163.0363;145.0257;135.0428;117.0311;89.0373;77.0370 | GQZ |
| 132* | 33.17 | [M-H]^-^ | 223.0614 | 223.0612 | 0.9 | C_11_H_12_O_5_ | 224.07 | Sinapic acid | 161.0617;91.0550 | GQZ |
| 133 | 33.22 | [M-H]^-^ | 793.2619 | 793.2561 | 7.4 | C_37_H_46_O_19_ | 794.26 | Epimedoside E | 793.2556;631.2025;514.1501;352.0943 | YYH |
| 134* | 33.36 | [M-H]^-^ | 359.0777 | 359.0772 | 1.3 | C_18_H_16_O_8_ | 360.08 | Rosmarinic acid | 197.0452;179.0329;161.0250;135.0451 | DS |
| 135 | 33.65 | [M+FA-H]^-^ | 977.5327 | 977.5327 | 0 | C_47_H_80_O_18_ | 932.53 | Notoginsenoside R1 | 977.5373;931.5280;769.4589;637.4311 | HS |
| 136 | 33.88 | [M-H]^-^ | 807.2696 | 807.2717 | -2.6 | C_38_H_48_O_19_ | 808.28 | Diphylloside B | 807.2675;645.2126;514.1436;352.0954 | YYH |
| 137 | 33.91 | [M+H]^+^ | 341.0649 | 341.0656 | -2 | C_18_H_12_O_7_ | 340.06 | salvianolic acid G | 341.0765;295.0589;279.0657;249.0565 | DS |
| 138* | 34.08 | [M-H]^-^ | 537.1017 | 537.1039 | -4 | C_27_H_22_O_12_ | 538.11 | Lithospermic acid | 493.1270;313.0698;295.0596;185.0233 | DS |
| 139 | 34.33 | [M+FA-H]^-^ | 707.2219 | 707.2193 | 3.7 | C_32_H_38_O_15_ | 662.22 | Epimedoside A | 661.2137;514.1457;353.1013 | YYH |
| 140 | 34.41 | [M-H]^-^ | 807.2678 | 807.2717 | -4.8 | C_38_H_48_O_19_ | 808.28 | Diphylloside B Isomer | / | YYH |
| 141 | 34.64 | [M-H]^-^ | 431.0992 | 431.0984 | 1.9 | C_21_H_20_O_10_ | 432.11 | kaempferol-3-rhamnoside | 431.0925;285.0372;255.0269 | MH |
| 142 | 35.05 | [M+H]^+^ | 314.1373 | 314.1387 | -4.4 | C_18_H_19_NO_4_ | 313.13 | Lyciumide A | 314.1427;177.0547;145.0281;117.0332 | GQZ |
| 143 | 35.21 | [M-H]^-^ | 691.2258 | 691.2244 | 2.1 | C_33_H_40_O_16_ | 692.23 | sagittasine C | 691.2309;529.1678;383.1116 | YYH |
| 144* | 35.38 | [M+FA-H]^-^ | 845.49 | 845.4904 | -0.5 | C_42_H_72_O_14_ | 800.49 | Ginsenoside Rg1 | 845.4927;799.4825;637.4175;161.0444 | HS |
| 145* | 35.57 | [M+FA-H]^-^ | 991.556 | 991.5483 | 6.1 | C_48_H_82_O_18_ | 946.55 | Ginsenoside Re | 991.5489;945.5419;783.4851;637.4270;619.4290 | HS |
| 146 | 35.94 | [M+H]^+^ | 287.1645 | 287.1642 | 1.1 | C_18_H_22_O_3_ | 286.16 | epicryptoacetalide | 287.1620;231.1005;185.0949 | DS |
| 147* | 36.27 | [M-H]^-^ | 717.1486 | 717.1461 | 3.5 | C_36_H_30_O_16_ | 718.15 | Salvianolic acid B | 717.1432;519.0936;493.1208;321.0377 | DS |
| 148 | 37.25 | [M+H]^+^ | 355.1168 | 355.1176 | -2.3 | C_20_H_18_O_6_ | 354.11 | Desmethylicaritin | 355.1176;297.0817;279.0658;177.0695 | YYH |
| 149 | 37.51 | [M-H]^-^ | 179.1063 | 179.1067 | -2 | C_11_H_14_O_2_ | 178.1 | Methyleugenol | 133.1029;116.0638;105.0706;91.0560 | XX |
| 150 | 37.61 | [M+H]^+^ | 314.138 | 314.1387 | -2.2 | C_18_H_19_NO_4_ | 313.13 | Lyciumide A isomer | 314.1409;177.0539145;145.0291;121.0658 | GQZ |
| 151 | 37.72 | [M-H]^-^ | 835.2664 | 835.2666 | -0.3 | C_39_H_48_O_20_ | 836.27 | demethylanhydroicaritin-7-O-glucopyranosyl-3-O-acetylatedrhamnopyranosyl-xylopyranoside | 835.2660;673.2152;529.1756;353.1008 | YYH |
| 152* | 37.74 | [M-H]^-^ | 253.0512 | 253.0506 | 2.2 | C_15_H_10_O_4_ | 254.06 | Daidzein | 253.0488;223.0396;208.0552;197.0612 | BGZ |
| 153* | 38.16 | [M-H]^-^ | 717.1442 | 717.1461 | -2.7 | C_36_H_30_O_16_ | 718.15 | Salvianolic acid Y | 717.1450;519.0934;339.0485;321.0386;295.0591 | DS |
| 154 | 38.33 | [M+H]^+^ | 355.1188 | 355.1176 | 3.3 | C_20_H_18_O_6_ | 354.11 | Asarinin | / | XX |
| 155 | 38.76 | [M+FA-H]^-^ | 883.2842 | 883.2877 | -0.2 | C_39_H_50_O_20_ | 838.29 | Epimedin A1 | 837.2850;675.2255;367.1170 | YYH |
| 156 | 39.03 | [M-H]^-^ | 283.0625 | 283.0612 | 4.6 | C_16_H_12_O_5_ | 284.07 | Acacetin | 283.0593;268.0375;239.0340;211.0381 | GQZ |
| 157* | 39.24 | [M-H]^-^ | 493.1126 | 493.114 | -2.9 | C_26_H_22_O_10_ | 494.12 | Salvianolic acid A | 493.1155;313.0721;295.0592;185.0233 | DS |
| 158* | 39.72 | [M+FA-H]^-^ | 883.2851 | 883.2877 | -3 | C_39_H_50_O_20_ | 838.29 | Epimedin A | 837.2787;675.2235;367.1159;366.1071 | YYH |
| 159* | 40.44 | [M+FA-H]^-^ | 853.2794 | 853.2772 | 2.6 | C_38_H_48_O_19_ | 808.28 | Epimedin B | 853.2782;807.2718;645.2161;366.1078 | YYH |
| 160* | 41.18 | [M+FA-H]^-^ | 867.2953 | 867.2928 | 2.8 | C_39_H_50_O_19_ | 822.29 | Epimedin C | 659.2361;366.1087 | YYH |
| 161 | 41.28 | [M-H]^-^ | 691.2243 | 691.2244 | -0.1 | C_33_H_40_O_16_ | 692.23 | CAS:1428239-94-4 | 691.2270;529.1705;383.1104 | YYH |
| 162* | 42.17 | [M+FA-H]^-^ | 721.2344 | 721.2349 | -0.7 | C_33_H_40_O_15_ | 676.24 | Icariin | 721.2425;529.1733;513.1779;409.1302;367.1181 | YYH |
| 163 | 42.59 | [M+H]^+^ | 491.0996 | 491.0984 | 2.5 | C_26_H_20_O_10_ | 492.11 | Isosalvianolic acid C | 491.0937;311.0587;293.0456 | DS |
| 164* | 44.81 | [M+H]^+^ | 187.039 | 187.039 | -2 | C_11_H_6_O_3_ | 186.03 | Psoralen | / | BGZ |
| 165 | 44.87 | [M-H]^-^ | 531.1857 | 531.1872 | -2.8 | C_27_H_32_O_11_ | 532.19 | Icaritin-3-O-rhamnopyranoside | 531.1832;384.1188;369.0937;341.1004;311.0458 | YYH |
| 166 | 45.81 | [M-H]^-^ | 819.2712 | 819.2717 | -0.6 | C_39_H_48_O_19_ | 820.28 | Anhydroicaritin-3-O-rhamnodide (1-2)-furan acid-7-O-glucoside | 919.2776;529.1800;367.1169;289.0945 | YYH |
| 167* | 45.95 | [M+FA-H]^-^ | 845.494 | 845.4904 | 4.2 | C_42_H_72_O_14_ | 800.49 | Ginsenoside Rf | 845.4932;799.4846;637.4346 | HS |
| 168 | 45.98 | [M-H]^-^ | 835.2633 | 835.2666 | -4 | C_39_H_48_O_20_ | 836.27 | Demethylanhydroicaritin-7-O-glucopyranosyl-3-O-acetylated rhamnopyranosyl-xylopyranoside isomer | 835.2705;673.2186;513.1769;383.1097 | YYH |
| 169 | 46.32 | [M-H]^-^ | 491.0986 | 491.0984 | 0.5 | C_26_H_20_O_10_ | 492.11 | Salvianolic acid C | 491.0965;311.0538;293.0446;179.0327 | DS |
| 170 | 46.33 | [M+FA-H]^-^ | 1017.4957 | 1017.4912 | 3.2 | C_48_H_76_O_20_ | 972.49 | Acankoreoside F | 1017.5070;971.4828;953.4892 | HS |
| 171* | 46.69 | [M-H]^-^ | 269.0447 | 269.0455 | -3.1 | C_15_H_10_O_5_ | 270.05 | Genistein | 269.0458;241.0478;213.0530 | / |
| 172* | 46.41 | [M+H]^+^ | 187.0388 | 187.039 | -0.9 | C_11_H_6_O_3_ | 186.03 | Isopsoralen | / | BGZ |
| 173 | 47.19 | [M+H]^+^ | 269.0428 | 269.0444 | -6.1 | C_15_H_8_O_5_ | 268.04 | Coumestrol | 269.0442;241.0486;197.0599 | BGZ |
| 174* | 47.72 | [M+FA-H]^-^ | 815.4817 | 815.4798 | 2.3 | C_41_H_70_O_13_ | 770.48 | Ginsenoside F5 | 815.4848;769.4751;637.4352;475.3736 | HS |
| 175 | 48.21 | [M+H]^+^ | 341.1388 | 341.1384 | 1.3 | C_20_H_20_O_5_ | 340.13 | Bavachromanol | 341.1403;323.1276;221.0804;203.0703;149.0227 | BGZ |
| 176 | 48.41 | [M+Cl]^-^ | 1245.6104 | 1245.604 | 5.1 | C_58_H_98_O_26_ | 1210.63 | Ginsenoside Ra1 | 1209.5982;1077.5692 | HS |
| 177 | 48.51 | [M+H]^+^ | 339.1217 | 339.1227 | -2.9 | C_20_H_18_O_5_ | 338.12 | Methyl tanshinonate Isomer | / | DS |
| 178 | 48.77 | [M-H]^-^ | 661.2154 | 661.2138 | 2.4 | C_32_H_38_O_15_ | 662.22 | Ikarisoside B | 661.2137;352.0929 | YYH |
| 179* | 48.97 | [M+FA-H]^-^ | 1153.6047 | 1153.6011 | 3.1 | C_54_H_92_O_23_ | 1108.6 | Ginsenoside Rb1 | 1153.6077;1107.5912;945.5388 | HS |
| 180 | 48.99 | [M+H]^+^ | 341.1376 | 341.1384 | -2.2 | C_20_H_20_O_5_ | 340.13 | Corylifol B | / | BGZ |
| 181 | 49.33 | [M+FA-H]^-^ | 829.4983 | 829.4955 | 3.4 | C_42_H_72_O_13_ | 784.5 | S-Ginsenoside Rg2 | 829.4959;783.4893;637.4321;475.3787 | HS |
| 182 | 49.44 | [M+H]^+^ | 339.1215 | 339.1227 | -3.5 | C_20_H_18_O_5_ | 338.12 | Methyl tanshinonate | 339.1201;321.1108;279.0630;267.0326 | DS |
| 183 | 49.46 | [M-H]^-^ | 661.2115 | 661.2138 | -3.5 | C_32_H_38_O_15_ | 662.22 | CAS: 1177873-66-3 | 661.2103;353.0996 | YYH |
| 184* | 49.69 | [M+FA-H]^-^ | 683.4343 | 683.4317 | -4.8 | C_36_H_62_O_9_ | 638.44 | Ginsenoside Rh1 | 683.4376;637.4322;475.3747 | HS |
| 185* | 49.83 | [M+FA-H]^-^ | 1123.5943 | 1123.5906 | 3.3 | C_53_H_90_O_22_ | 1078.59 | Ginsenoside Rc | 1123.5929;1077.5835;945.5423;915.5185 | HS |
| 186 | 49.84 | [M-H]^-^ | 1209.6351 | 1209.6274 | 6.5 | C_58_H_98_O_26_ | 1210.63 | Ginsenoside Ra2 | 1209.6206;1077.6045 | HS |
| 187 | 49.94 | [M-H]^-^ | 329.2346 | 329.2333 | 3.8 | C_18_H_34_O_5_ | 330.24 | 9,12,13-Trihydroxy-10-octadecenoic acid | 329.2341;229.1454;211.1340;171.1027 | GQZ |
| 188 | 50.03 | [M-H]^-^ | 529.171 | 529.1715 | -1 | C_27_H_30_O_11_ | 530.18 | Caohuoside C | 529.1722;382.1034;312.0593;297.0401;269.0449 | YYH |
| 189 | 50.13 | [M-H]^-^ | 631.2021 | 631.2032 | -3.2 | C_31_H_36_O_14_ | 632.21 | Demethylanhydroicaritin-3-O-rhamnopyranosyl-xylopyranoside | 631.2015;353.0957 | YYH |
| 190 | 50.22 | [M+FA-H]^-^ | 683.438 | 683.4376 | 0.6 | C_36_H_62_O_9_ | 638.44 | Ginsenoside F1 | 683.4378;637.4289;475.3736 | HS |
| 191* | 50.28 | [M-H]^-^ | 955.4952 | 955.4908 | 4.6 | C_48_H_76_O_19_ | 956.5 | Ginsenoside Ro | 955.4946;793.4285 | HS |
| 192* | 50.47 | [M+FA-H]^-^ | 1123.5891 | 1123.5906 | -1.3 | C_53_H_90_O_22_ | 1078.59 | Ginsenoside Rb2 | 1123.5991;1077.5836;945.5676 | HS |
| 193 | 50.61 | [M-H]^-^ | 529.1723 | 529.1715 | 1.4 | C_27_H_30_O_11_ | 530.18 | Caohuoside C Isomer | 529.1715;383.1113;312.0620;297.0364 | YYH |
| 194 | 50.75 | [M-H]^-^ | 401.0867 | 401.0878 | -4 | C_20_H_18_O_9_ | 402.1 | Versiconol acetate | 401.0831;357.0621;313.0695;269.0805 | / |
| 195 | 50.76 | [M+H]^+^ | 337.1083 | 337.1071 | 3.7 | C_20_H_16_O_5_ | 336.1 | Corylifol D | 337.1063;319.0959;279.0644;223.0768;167.0857 | BGZ |
| 196 | 50.8 | [M+FA-H]^-^ | 1195.6185 | 1195.6117 | 5.7 | C_56_H_94_O_24_ | 1150.61 | Quinquenoside R1 | 1195.6126;1149.6109;1107.5957;1089.5679 | HS |
| 197 | 51.01 | [M-H]^-^ | 925.4794 | 925.4802 | -0.9 | C_47_H_74_O_18_ | 926.49 | Chikusetsusaponin IV | 925.4802;763.4136;613.3830 | HS |
| 198 | 51.25 | [M-H]^-^ | 335.0924 | 335.0925 | -0.3 | C_20_H_16_O_5_ | 336.1 | Yinyanghuo C | 335.0910;319.0597;317.0796;277.0488 | YYH |
| 199 | 51.34 | [M+FA-H]^-^ | 1165.6067 | 1165.6011 | 4.8 | C_55_H_92_O_23_ | 1120.6 | Ginsenoside Rs1 | 1165.6213;1119.6046;1077.5791;1059.5778 | HS |
| 200* | 51.37 | [M+FA-H]^-^ | 991.5491 | 991.5483 | 0.8 | C_48_H_82_O_18_ | 946.55 | Ginsenoside Rd | 991.5551;945.5442;783.5012 | HS |
| 201 | 51.38 | [M-H]^-^ | 499.1599 | 499.161 | -2.1 | C_26_H_28_O_10_ | 500.17 | Ikarisoside A | 499.1611;353.1005 | YYH |
| 202 | 51.66 | [M+FA-H]^-^ | 1165.601 | 1165.6011 | -0.1 | C_55_H_92_O_23_ | 1120.6 | Ginsenoside Rs2 | 1165.6060;1119.5898;1077.5736;1059.5639 | HS |
| 203* | 51.78 | [M-H]^-^ | 793.4406 | 793.438 | 3.3 | C_42_H_66_O_14_ | 794.45 | Chikusetsusaponin-Iva | 793.4340;631.3876;455.3454 | HS |
| 204 | 52.17 | [M+H]^+^ | 293.0825 | 293.0808 | 5.7 | C_18_H_12_O_4_ | 292.07 | Przewaquinone B | 293.0798;249.0910;193.1023;178.0765 | DS |
| 205* | 52.35 | [M-H]^-^ | 675.2314 | 675.2294 | 2.9 | C_33_H_40_O_15_ | 676.24 | Baohuoside VII | 675.2285;367.1186;352.0894 | YYH |
| 206 | 52.45 | [M+H]^+^ | 274.2745 | 274.2741 | 1.6 | C_16_H_35_NO_2_ | 273.27 | Lauryldiethanolamine | 274.2752;256.2645;70.0645 | / |
| 207 | 52.67 | [M+FA-H]^-^ | 1033.5643 | 1033.5589 | 5.2 | C_50_H_84_O_19_ | 988.56 | Quinquenoside III | 1033.5635;987.5549;945.5373;927.5238;783.5001 | HS |
| 208 | 53.04 | [M-H]^-^ | 645.2224 | 645.2189 | 5.5 | C_32_H_38_O_14_ | 646.23 | Sagittatoside B | 645.2169;366.1089;351.0844;323.0909 | YYH |
| 209 | 53.16 | [M+FA-H]^-^ | 575.1784 | 575.177 | 2.4 | C_27_H_30_O_11_ | 530.18 | Icariside I | 529.1710;367.1181;352.0904;297.0434 | YYH |
| 210 | 53.17 | [M-H]^-^ | 659.2338 | 659.2345 | -1.1 | C_33_H_40_O_14_ | 660.24 | 2″-O-rhamnosylikariside Ⅱ | 659.2339;366.1092;351.0851;323.0499 | YYH |
| 211 | 53.26 | [M+FA-H]^-^ | 1033.5659 | 1033.5589 | 6.8 | C_50_H_84_O_19_ | 988.56 | Pseudoginsenoside Rc1 | 1033.5677;987.5547;945.5352;927.5173;783.4777 | HS |
| 212 | 53.58 | [M+FA-H]^-^ | 797.4686 | 797.4693 | -0.9 | C_41_H_68_O_12_ | 752.47 | Hebevinoside VI | 797.4637;751.4648;619.4355 | HS |
| 213 | 53.82 | [M+FA-H]^-^ | 811.4839 | 811.4849 | -1.3 | C_42_H_70_O_12_ | 766.49 | Ginsenoside Rg6 | 811.5023;765.4807;619.4170 | HS |
| 214 | 53.86 | [M+H]^+^ | 281.0801 | 281.0808 | -2.3 | C_17_H_12_O_4_ | 280.07 | Nortanshinone | 281.0776;263.0711;207.0774;161.0220;147.0433 | DS |
| 215 | 53.86 | [M+FA-H]^-^ | 797.4688 | 797.4693 | -0.6 | C_41_H_68_O_12_ | 752.47 | Melilotoside B | 797.4777;751.4621;619.4217 | HS |
| 216* | 54.07 | [M+FA-H]^-^ | 811.4846 | 811.4849 | -0.4 | C_42_H_70_O_12_ | 766.49 | Ginsenoside F4 | 811.4900;765.4774;619.4226 | HS |
| 217* | 54.47 | [M-H]^-^ | 513.1772 | 513.1766 | 1.1 | C_27_H_30_O_10_ | 514.18 | Baohuoside Ⅰ | 513.1773;366.1106;351.0847;323.0853 | YYH |
| 218 | 54.5 | [M+H]^+^ | 341.1386 | 341.1384 | 0.7 | C_20_H_20_O_5_ | 340.13 | Bakuchalcone | 341.1358;323.1260;221.0781;203.0715;149.0227 | BGZ |
| 219 | 54.63 | [M+FA-H]^-^ | 665.428 | 665.427 | 1.5 | C_36_H_360_O_8_ | 922.78 | Ginsenoside Rh4 | 665.4323;619.4238 | HS |
| 220 | 54.75 | [M-H]^-^ | 793.438 | 793.438 | 0 | C_42_H_66_O_14_ | 794.45 | Zingibroside R1 | 793.4365;613.3714;569.3833;455.3521 | HS |
| 221 | 54.82 | [M+H]^+^ | 311.1286 | 311.1278 | 2.6 | C_19_H_18_O_4_ | 310.12 | TanshinoneⅡB | 311.1270;283.1379;267.1370;249.0911;225.0937 | DS |
| 222* | 54.99 | [M-H]^-^ | 321.1123 | 321.1132 | -2.9 | C_20_H_18_O_4_ | 322.12 | Neobavaisoflavone | 321.1111;277.0488;265.0505 | BGZ |
| 223* | 54.99 | [M+FA-H]^-^ | 665.426 | 665.427 | -1.5 | C_36_H_60_O_8_ | 620.43 | Ginsenoside Rk3 | 665.4285;619.4175 | HS |
| 224 | 55.39 | [M+FA-H]^-^ | 829.4954 | 829.4955 | -0.1 | C_42_H_72_O_13_ | 784.5 | Ginsenoside F2 | 829.5027;783.4933;621.4426;459.3908 | HS |
| 225 | 55.48 | [M-H]^-^ | 311.2242 | 311.2228 | 4.6 | C_18_H_32_O_4_ | 312.23 | 12,13-dihydroxy-9Z,15Z-octadecadienoic acid | 311.2219;293.2135;275.2020;223.1701 | GQZ |
| 226* | 54.1 | [M-H]^-^ | 323.1281 | 323.1289 | -2.4 | C_20_H_20_O_4_ | 324.14 | Bavachin | 323.1274;203.0707;119.0503 | BGZ |
| 227 | 55.63 | [M+FA-H]^-^ | 829.5026 | 829.4955 | 8.6 | C_42_H_72_O_13_ | 784.5 | Ginsenoside Rg3 | 829.4979;783.4916;621.4416;459.3822 | HS |
| 228 | 55.83 | [M+H]^+^ | 309.1088 | 309.1121 | -1.4 | C_19_H_16_O_4_ | 308.1 | Tanshinaldehyde | 309.1105;265.1208;250.1007;223.0743;192.0938 | DS |
| 229 | 56.07 | [M+H]^+^ | 261.1837 | 261.1849 | -4.6 | C_17_H_24_O_2_ | 260.18 | panaxydol | 105.0326;81.0685;77.0374; | HS |
| 230 | 56.27 | [M-H]^-^ | 295.0975 | 295.0976 | -0.3 | C_18_H_16_O_4_ | 296.1 | Danshenxinkun A | 295.0979;277.0855;265.0878;254.9857;237.0896 | DS |
| 231* | 56.52 | [M-H]^-^ | 269.0454 | 269.0455 | -0.5 | C_15_H_10_O_5_ | 270.05 | Apigenin | 269.0449;241.0526;224.0524;201.0554;181.0645 | GQZ/MH |
| 232* | 56.78 | [M+H]^+^ | 321.1109 | 321.1121 | -3.8 | C_20_H_16_O_4_ | 320.1 | Corylin | 321.1103;279.0650;137.0223 | BGZ |
| 233 | 57.01 | [M+H]^+^ | 297.147 | 297.1485 | -5.1 | C_19_H_20_O_3_ | 296.14 | Isocryptotanshinone | 297.1473;269.1607;253.1583;237.0915;211.1644 | DS |
| 234 | 57.01 | [M+H]^+^ | 337.1412 | 337.1434 | -6.6 | C_21_H_20_O_4_ | 336.14 | Danshenxinkun D | 337.1412 | DS |
| 235 | 57.25 | [M+FA-H]^-^ | 871.5086 | 871.5061 | 2.9 | C_44_H_74_O_14_ | 826.51 | Ginsenoside Rs3 | 871.5047;825.5020;783.4885;459.3876 | HS |
| 236 | 57.27 | [M+FA-H]^-^ | 723.3788 | 723.3809 | -2.8 | C_33_H_58_O_14_ | 678.38 | Shinbarometin | 677.3768;415.1454;397.1348;279.2343 | / |
| 237* | 57.27 | [M-H]^-^ | 335.0927 | 335.0925 | 0.6 | C_20_H_16_O_5_ | 336.1 | psoralidin | 335.0915;280.0354;266.9793;252.0428;227.0094 | BGZ |
| 238* | 57.31 | [M-H]^-^ | 323.1289 | 323.1289 | 0.1 | C_20_H_20_O_4_ | 324.14 | Isobavachalcone | 323.1285;203.0698;119.0505 | BGZ |
| 239 | 57.81 | [M+FA-H]^-^ | 723.3805 | 723.3809 | -0.5 | C_33_H_58_O_14_ | 678.38 | Gingerglycolipid B | 677.3765;415.1446;397.1353;279.2328 | / |
| 240* | 58.02 | [M+H]^+^ | 279.1005 | 279.1016 | -3.8 | C_18_H_14_O_3_ | 278.09 | Dihydrotanshinone I | 279.10212;261.0905;233.0959;205.1004;149.0219 | DS |
| 241* | 58.41 | [M+FA-H]^-^ | 811.488 | 811.4849 | 3.8 | C_42_H_70_O_12_ | 766.49 | Ginsenoside Rk1 | 811.4900;765.4771;603.4276 | HS |
| 242 | 58.45 | [M-H]^-^ | 323.1301 | 323.1289 | 3.8 | C_20_H_20_O_4_ | 324.14 | Bavachalcone | 323.1292;203.0719;119.0502 | BGZ |
| 243 | 58.68 | [M+H]^+^ | 315.1596 | 315.1591 | 1.6 | C_19_H_22_O_4_ | 314.15 | Neocryptotanshinone | 297.1495;279.1347;254.0924;227.0696 | DS |
| 244* | 58.7 | [M+FA-H]^-^ | 811.4869 | 811.4849 | 2.4 | C_42_H_70_O_12_ | 766.49 | Ginsenoside Rg5 | 811.4849;765.4749;603.4259 | HS |
| 245 | 58.74 | [M+H]^+^ | 281.1178 | 281.1172 | 2.1 | C_18_H_16_O_3_ | 280.11 | Trijuganone B | 281.0508;263.1022;235.1104;192.0894 | DS |
| 246* | 59.24 | [M+H]^+^ | 339.1579 | 339.1591 | -3.5 | C_21_H_22_O_4_ | 338.15 | bavachinin | 339.1586;283.0959;271.0951;219.1018;147.0438 | BGZ |
| 247 | 59.26 | [M+H]^+^ | 313.1066 | 313.1071 | -1.4 | C_18_H_16_O_5_ | 312.1 | tanshindiol A | 313.1080;193.0489;165.0522;147.0431 | DS |
| 248 | 59.46 | [M+H]^+^ | 391.189 | 391.1904 | -3.5 | C_25_H_26_O_4_ | 390.18 | Corylifol A | 391.2999;267.0655;239.0718;149.0232 | BGZ |
| 249* | 60.48 | [M+H]^+^ | 297.1487 | 297.1485 | 0.6 | C_19_H_20_O_3_ | 296.14 | Cryptotanshinone | 297.1479;279.1378;251.1419;137.0902 | DS |
| 250 | 60.56 | [M+H]^+^ | 277.0865 | 277.0859 | 2.1 | C_18_H_12_O_3_ | 276.08 | Tanshinone I | 277.0864;249.0915;231.0812;178.0777;152.0627 | DS |
| 251 | 61.37 | [M+H]^+^ | 279.0998 | 279.1016 | -6.3 | C_18_H_14_O_3_ | 278.09 | Dihydroisotanshinone II | 261.0925;233.0990;190.0743;149.0233 | DS |
| 252* | 62.59 | [M+H]^+^ | 295.1332 | 295.1329 | 1.1 | C_19_H_18_O_3_ | 294.13 | Tanshinone IIA | 295.1332;277.1198;262.0974;252.0766;235.0746;207.0806 | DS |

Notes:1) GQZ: Lycii Fructus; SZ: Hirudo; DS: Salviae Miltiorrhizae Radix Et Rhizoma; MH: Ephedrae Herba; BGZ: Psoraleae Fructus; XX: Asari Radix Et Rhizoma; YYH: Epimedii Folium; HS: Ginseng Radix Et Rhizoma Rubra.

2) *: identified by comparison with reference standards.

# Supplementary Table 2. Identification of chemical compounds in test solution of SXSM by GC-MS

| No. | RT（min） | Formula | M.W. | Identification | CAS number | Score |
| --- | --- | --- | --- | --- | --- | --- |
| GC001 | 4.3 | C_4_H_10_O_2_ | 90.07 | 2,3-Butanediol | 513-85-9 | 97 |
| GC002 | 4.95 | C_6_H_12_O_2_ | 116.08 | Acetic acid, butyl ester | 123-86-4 | 97.85 |
| GC003 | 7.12 | C_3_H_8_O | 60.06 | 2-Propanol | 67-63-0 | 81.2 |
| GC004 | 8.27 | C_7_H_6_O | 106.04 | Benzaldehyde | 100-52-7 | 94.46 |
| GC005 | 10.53 | C_6_H_6_O_3_ | 126.03 | Maltol | 118-71-8 | 96.38 |
| GC006 | 11.02 | C_6_H_8_O_4_ | 144.04 | 2,3-Dihydro-3,5-dihydroxy-6-methyl-4H-pyran-4-one | 28564-83-2 | 94.32 |
| GC007 | 11.4 | C_7_H_6_O_2_ | 122.04 | Benzoic acid | 65-85-0 | 87.81 |
| GC008 | 11.68 | C_9_H_8_O_3_ | 164.05 | 2-Propenoic acid, 3-(2-hydroxyphenyl)-, (E)- | 614-60-8 | 93.66 |
| GC009 | 11.89 | C_6_H_6_O_3_ | 126.03 | 5-Hydroxymethylfurfural | 67-47-0 | 86.39 |
| GC010 | 12.56 | C_7_H_6_O_3_ | 138.03 | Benzoic acid, 2-hydroxy- | 69-72-7 | 88.84 |
| GC011 | 13.16 | C_7_H_6_O_2_ | 122.04 | Benzaldehyde, 4-hydroxy- | 123-08-0 | 92.08 |
| GC012 | 13.83 | C_9_H_8_O_2_ | 148.05 | 2-Propenoic acid, 3-phenyl- | 621-82-9 | 88.99 |
| GC013 | 14.84 | C_6_H_6_N_2_O_2_ | 138.04 | 3-Pyridinecarboxylic acid, 6-amino- | 3167-49-5 | 82.82 |
| GC014 | 15.2 | C_8_H_8_O_4_ | 168.04 | Benzoic acid, 4-hydroxy-3-methoxy- | 121-34-6 | 83.35 |
| GC015 | 16.8 | C_11_H_6_O_3_ | 186.03 | Isopsoralen | 523-50-2 | 95.75 |
| GC016 | 16.96 | C_10_H_14_O_4_ | 198.09 | Benzenemethanol, 3,4,5-trimethoxy- | 3840-31-1 | 81.4 |
| GC017 | 17.21 | C_11_H_6_O_3_ | 186.03 | Psoralene | 66-97-7 | 96.8 |
| GC018 | 17.73 | C_16_H_32_O_2_ | 256.24 | Hexadecanoic acid | 57-10-3 | 92.74 |
| GC019 | 18.24 | C_17_H_24_O | 244.18 | Falcarinol | 21852-80-2 | 95.09 |
| GC020 | 18.87 | C_18_H_32_O_2_ | 280.24 | 9,12-Octadecadienoic acid (Z, Z)- | 60-33-3 | 97.07 |
| GC021 | 19.29 | C_18_H_24_O | 256.18 | Phenol, 4-(3,7-dimethyl-3-ethenylocta-1,6-dienyl)- | 93998-10-8 | 80.83 |
| GC022 | 19.72 | C_37_H_76_O | 536.59 | 1-Heptatriacotanol | 105794-58-9 | 84.92 |
| GC023 | 19.77 | C_14_H_22_O_2_ | 222.16 | 2,2,6-Trimethyl-1-(3-methylbuta-1,3-dienyl)-7-oxabicyclo [4.1.0] heptan-3-ol | 1427305-74-5 | 81.06 |
| GC024 | 20.01 | C_12_H_20_O | 180.15 | Cyclohexene, 1,5,5-trimethyl-6-acetylmethyl | 211563-96-1 | 84.8 |
| GC025 | 20.69 | C_21_H_36_O_6_ | 384.25 | beta. -D-Mannofuranoside, farnesyl- | 998650-87-2 | 83.51 |
| GC026 | 21.05 | C_35_H_68_O_5_ | 568.51 | Hexadecanoic acid, 1-(hydroxymethyl)-1,2-ethanediyl ester | 761-35-3 | 88.1 |
| GC027 | 21.24 | C_19_H_30_O_2_ | 290.22 | 10,13-Octadecadiynoic acid, methyl ester | 18202-24-9 | 81.48 |
| GC028 | 23.37 | C_21_H_38_O_4_ | 354.28 | 9,12-Octadecadienoic acid (Z, Z)-, 2-hydroxy-1-(hydroxymethyl)ethyl ester | 3443-82-1 | 92.42 |

# Supplementary Table 3. Representative identified monomer components from SXSM by UPLC-QTOF-MS/MS in comparing with standard TCM chemicals

| **No.** | **Retention time/min** | **Adduct ion** | ***m/z*** | **Molecular formula** | **Molecular**  **weight** | **MS/MS data** | **Inferred compound** |
| --- | --- | --- | --- | --- | --- | --- | --- |
| 088 | 23.49 | [2M-H]^-^ | 731.1839 | C_17_H_18_O_9_ | 366.1 | 731.1864;365.0867;203.0338;159.0447 | Psoralenoside |
| 092 | 24.23 | [2M-H]^-^ | 731.1816 | C_17_H_18_O_9_ | 366.1 | 731.1849;365.0858;203.0350;159.0451 | Isopsoralenoside |
| 164 | 44.81 | [M+H] ^+^ | 187.039 | C_11_H_6_O_3_ | 186.03 | / | Psoralen |
| 171 | 46.41 | [M+H] ^+^ | 187.0388 | C_11_H_6_O_3_ | 186.03 | / | Isopsoralen |
| 222 | 54.99 | [M-H]^-^ | 321.1123 | C_20_H_18_O_4_ | 322.12 | 321.1111;277.0488;265.0505 | Neobavaisoflavone |
| 232 | 56.78 | [M+H] + | 321.1109 | C_20_H_16_O_4_ | 320.1 | 321.1103;279.0650;137.0223 | Corylin |
| 226 | 55.61 | [M-H]^-^ | 323.1289 | C_20_H_20_O_4_ | 324.14 | 323.1285;203.0698;119.0505 | Bavachin |
| 237 | 57.27 | [M-H]^-^ | 335.0927 | C_20_H_16_O_5_ | 336.1 | 335.0915;280.0354;266.9793;252.0428;227.0094 | Psoralidin |
| 246 | 59.24 | [M+H] ^+^ | 339.1579 | C_21_H_22_O_4_ | 338.15 | 339.1586;283.0959;271.0951;219.1018;147.0438 | Bavachinin |

# Supplementary Table 4. Summary of compound targets

| gene symbol | gene symbol | gene symbol | gene symbol | gene symbol | gene symbol | gene symbol | gene symbol |
| --- | --- | --- | --- | --- | --- | --- | --- |
| AADAT | CCR1 | EIF2AK3 | HEXB | MBNL3 | PLA2G1B | SLC8A1 | EPHX3 |
| ABAT | CCR2 | EIF4A1 | HIF1A | MC1R | PLA2G2A | SLC9A1 | EPHX4 |
| ABCB1 | CCR3 | ELANE | HK1 | MCHR1 | PLA2G5 | SLCO1B1 | ERCC1 |
| ABCB11 | CCR4 | ENGASE | HK2 | MCL1 | PLA2G7 | SLK | ERCC4 |
| ABCC1 | CCR5 | ENPEP | HLA-A | MDM2 | PLAA | SMO | FARS2 |
| ABCC9 | CCR9 | EP300 | HLA-DRB1 | ME1 | PLAT | SMYD2 | FOS |
| ABCG2 | CD274 | EPHA1 | HMGCR | MELK | PLAU | SNCA | FOXO3 |
| ABL1 | CD38 | EPHA2 | HMOX1 | MERTK | PLAUR | SOAT1 | FOXP3 |
| ACACB | CDA | EPHA3 | HPGD | MET | PLD1 | SOAT2 | GBA3 |
| ACE | CDC25A | EPHA4 | HPGDS | METAP2 | PLEC | SORD | GSS |
| ACE2 | CDC25B | EPHA5 | HPRT1 | MGAM | PLG | SORT1 | GSTM1 |
| ACHE | CDC25C | EPHA6 | HPSE | MGLL | PLK1 | SPHK1 | HMGA1 |
| ACLY | CDC42BPA | EPHA7 | HRAS | MIF | PLK4 | SPHK2 | HPD |
| ACVRL1 | CDC7 | EPHA8 | HRH1 | MKNK2 | PNMT | SQLE | IBSP |
| ADA | CDK1 | EPHB1 | HRH2 | MLNR | PNP | SRC | IL12A |
| ADAM10 | CDK11A | EPHB2 | HRH3 | MME | POLA1 | SRD5A1 | IL12B |
| ADAM17 | CDK11B | EPHB3 | HRH4 | MMP1 | POLB | SRD5A2 | IL13 |
| ADAM9 | CDK13 | EPHB4 | HSD11B1 | MMP10 | PON1 | SREBF2 | IL5 |
| ADAMTS4 | CDK14 | EPHB6 | HSD11B2 | MMP12 | PPARA | SSTR1 | KCNC1 |
| ADAMTS5 | CDK15 | EPHX2 | HSD17B1 | MMP13 | PPARD | SSTR2 | KRAS |
| ADCY1 | CDK16 | ERAP2 | HSD17B2 | MMP14 | PPARG | SSTR3 | LARS2 |
| ADK | CDK17 | ERBB2 | HSD17B3 | MMP15 | PPIA | SSTR4 | MITF |
| ADORA1 | CDK18 | ERBB3 | HSP90AA1 | MMP16 | PPM1A | SSTR5 | MVK |
| ADORA2A | CDK19 | ERBB4 | HSP90AB1 | MMP2 | PPM1B | ST3GAL3 | MYL2 |
| ADORA2B | CDK2 | ERCC5 | HSP90B1 | MMP26 | PPP1CC | ST6GAL1 | NFKB2 |
| ADORA3 | CDK3 | ERN1 | HSPA1A | MMP3 | PPP2CA | STAT1 | NR0B1 |
| ADRA1A | CDK4 | ESR1 | HSPA5 | MMP7 | PPP2R5A | STAT2 | NR1I2 |
| ADRA1B | CDK5 | ESR2 | HSPA8 | MMP8 | PPP5C | STAT3 | NR5A1 |
| ADRA1D | CDK5R1 | ESRRA | HTR1A | MMP9 | PRCP | STAT4 | NRAS |
| ADRA2A | CDK6 | ESRRB | HTR1B | MPEG1 | PREP | STAT6 | OTP |
| ADRA2B | CDK7 | EZH1 | HTR1D | MPG | PRF1 | STK17B | OXSM |
| ADRA2C | CDK9 | EZH2 | HTR1E | MPI | PRKACA | STS | P2RX2 |
| ADRB1 | CDKL2 | EZR | HTR2A | MPO | PRKCA | SUV39H1 | P4HB |
| ADRB2 | CDKL5 | F10 | HTR2B | MTNR1A | PRKCB | SUZ12 | PFAS |
| ADRB3 | CES1 | F11 | HTR2C | MTNR1B | PRKCD | SYK | PIGT |
| ADSS | CES2 | F13A1 | HTR3A | MTOR | PRKCE | TAAR1 | PINX1 |
| AGL | CFD | F2 | HTR4 | MYLK | PRKCG | TACR1 | PLD2 |
| AGTR1 | CFTR | F2R | HTR5A | NAAA | PRKCH | TACR2 | PNPLA2 |
| AGTR2 | CHEK1 | F3 | HTR6 | NAALAD2 | PRKCI | TACR3 | POT1 |
| AHCY | CHEK2 | F7 | HTR7 | NAE1 | PRKCQ | TAOK2 | PPWD1 |
| AHCYL1 | CHIA | FAAH | ICAM1 | NAMPT | PRKD1 | TAOK3 | PRNP |
| AHR | CHRM1 | FABP1 | ICK | NAT1 | PRKD2 | TAS1R1 | PSAT1 |
| AKR1A1 | CHRM2 | FABP2 | IDE | NCOR1 | PRKDC | TAS1R3 | RAPGEF1 |
| AKR1B1 | CHRM3 | FABP3 | IDH1 | NCOR2 | PRLR | TAS2R31 | RBM48 |
| AKR1B10 | CHRM4 | FABP4 | IDO1 | NCSTN | PRMT1 | TBK1 | RHAG |
| AKR1B15 | CHRM5 | FABP5 | IDO2 | NEK1 | PRMT3 | TBXA2R | RPL10A |
| AKR1C1 | CHRNA2 | FAP | IGF1R | NEK2 | PRMT5 | TBXAS1 | RPS6KA2 |
| AKR1C2 | CHRNA3 | FBP1 | IGFBP1 | NEK6 | PRMT6 | TDO2 | RPS6KA6 |
| AKR1C3 | CHRNA4 | FCER2 | IGFBP2 | NEU2 | PRMT8 | TDP1 | RPS6KB2 |
| AKR1C4 | CHRNA5 | FDFT1 | IGFBP3 | NEU3 | PRSS1 | TDP2 | RRN3 |
| AKR1E2 | CHRNA6 | FEN1 | IGFBP4 | NEU4 | PRSS3 | TEK | SCARB1 |
| AKT1 | CHRNA7 | FFAR1 | IGFBP5 | NFE2L2 | PRTN3 | TERT | SETX |
| AKT2 | CHRNB2 | FGF1 | IGFBP6 | NFKB1 | PSEN1 | TGFB1 | SGK2 |
| AKT3 | CHRNB3 | FGF2 | IKBKB | NGFR | PSEN2 | TGFBR1 | SLC22A7 |
| ALB | CHRNB4 | FGFR1 | IKBKE | NISCH | PSENEN | TGM2 | SLC22A8 |
| ALDH1A1 | CHRND | FGFR3 | IL1B | NME1 | PSMB5 | TH | SLC2A4 |
| ALDH2 | CHUK | FGR | IL2 | NME2 | PTAFR | THRA | SLC44A1 |
| ALDH3A1 | CISD1 | FKBP1A | ILK | NME3 | PTGDR | THRB | SLC6A14 |
| ALDH5A1 | CLK1 | FLT1 | IMPDH1 | NMUR2 | PTGDR2 | TK1 | SLC6A15 |
| ALK | CLK2 | FLT3 | IMPDH2 | NNMT | PTGER1 | TK2 | SLC6A18 |
| ALOX12 | CLK3 | FLT4 | INMT | NOD2 | PTGER2 | TLK1 | SLC6A19 |
| ALOX15 | CLK4 | FNTA | INSR | NOS1 | PTGER3 | TLR4 | SLCO1B3 |
| ALOX5 | CMA1 | FNTB | IRAK1 | NOS2 | PTGER4 | TLR9 | SLCO2B1 |
| ALOX5AP | CNR1 | FOLH1 | IRAK4 | NOS3 | PTGES | TNF | SLPI |
| ALPG | CNR2 | FPGS | ITGA2B | NOTUM | PTGFR | TNKS | SOD1 |
| ALPL | COMT | FPR1 | ITGA4 | NOX1 | PTGIR | TNKS2 | SPICE1 |
| AMPD1 | COQ8B | FRK | ITGA5 | NOX4 | PTGS1 | TNNC1 | SREBF1 |
| AMPD2 | CPA1 | FTO | ITGAL | NPC1L1 | PTGS2 | TNNI3 | STK11 |
| AMPD3 | CPA3 | FUCA1 | ITGAV | NPFFR1 | PTK2 | TNNI3K | SULT1A1 |
| AMY1A | CPB1 | FUCA2 | ITGB1 | NPFFR2 | PTK2B | TNNT2 | TAF3 |
| AMY2A | CPB2 | FUT4 | ITGB2 | NPY1R | PTK6 | TOP1 | TIMP2 |
| ANPEP | CPT1A | FUT7 | ITGB3 | NPY4R | PTP4A1 | TOP2A | TMEM37 |
| AOC3 | CREBBP | FYN | ITGB5 | NPY5R | PTP4A2 | TP53 | TMPRSS11D |
| APEX1 | CRHR1 | G6PD | ITGB6 | NQO1 | PTP4A3 | TPMT | UGT1A1 |
| APH1A | CSF1R | GAA | ITGB7 | NQO2 | PTPA | TPSAB1 | UGT1A10 |
| APH1B | CSK | GABBR1 | ITK | NR1H2 | PTPN1 | TREH | UGT1A3 |
| APP | CSNK1A1 | GABBR2 | JAK1 | NR1H3 | PTPN11 | TRHR | UGT1A6 |
| AR | CSNK1G1 | GABRA1 | JAK2 | NR1H4 | PTPN2 | TRPA1 | UGT1A7 |
| ARG1 | CSNK2A1 | GABRA2 | JAK3 | NR3C1 | PTPN22 | TRPC3 | UGT1A8 |
| ASIC3 | CSNK2A2 | GABRA3 | JUN | NR3C2 | PTPN6 | TRPC6 | UGT1A9 |
| ASNS | CSNK2A3 | GABRA5 | KARS | NR4A1 | PTPRA | TRPM8 | UGT2B15 |
| ATAD2 | CTBP2 | GABRB2 | KAT2B | NR4A2 | PTPRC | TRPV1 | UGT2B4 |
| ATIC | CTNNB1 | GABRB3 | KCNA3 | NRP1 | PTPRCAP | TRPV3 | UPP2 |
| ATP1A1 | CTSA | GABRG2 | KCNA5 | NTRK1 | PTPRF | TRPV4 | ZNF238 |
| AURKA | CTSB | GABRR1 | KCNH2 | NTRK2 | PTPRS | TSPO | ZNF622 |
| AURKB | CTSC | GALR1 | KCNJ1 | NTRK3 | PYGB | TTK | AMD1 |
| AVPR1A | CTSD | GALR2 | KCNK3 | NTSR1 | PYGL | TTL | AMY1B |
| AVPR2 | CTSE | GALR3 | KCNMA1 | NTSR2 | PYGM | TTR | AMY1C |
| AXL | CTSF | GANAB | KDM1A | NUAK1 | QDPR | TUBB1 | AZGP1 |
| AZU1 | CTSG | GANC | KDM2A | OAT | QPCT | TUBB3 | CFB |
| BACE1 | CTSH | GAPDH | KDM3A | ODC1 | RAF1 | TXK | CSNK1G2 |
| BACE2 | CTSK | GBA | KDM4A | OGA | RASGRP3 | TYK2 | GALK1 |
| BAD | CTSL | GBA2 | KDM4B | OGT | RBBP4 | TYMP | ICAM2 |
| BAZ2A | CTSS | GCGR | KDM4C | OPRD1 | RBBP7 | TYMS | ISG20 |
| BAZ2B | CTSV | GCK | KDM4D | OPRK1 | RELA | TYR | LCN2 |
| BBOX1 | CX3CR1 | GCLC | KDM4E | OPRL1 | REN | TYRO3 | ME2 |
| BCAT1 | CXCR1 | GFPT1 | KDM5B | OPRM1 | RET | UBA6 | MTAP |
| BCAT2 | CXCR2 | GLA | KDM5C | P2RX3 | RGS4 | UGCG | PAH |
| BCHE | CXCR3 | GLB1 | KDM6B | P2RX7 | RIPK2 | UGT2B7 | PSPH |
| BCL2 | CXCR4 | GLI1 | KDR | P2RY12 | RNASEH1 | UPP1 | QPCTL |
| BCL2A1 | CYP11B1 | GLO1 | KHK | P2RY2 | RNASEL | VARS | SOD2 |
| BCL2L1 | CYP11B2 | GLP1R | KIF11 | P2RY4 | RNPEP | VCAM1 | TREM1 |
| BCL2L2 | CYP17A1 | GLRA1 | KISS1R | P2RY6 | ROCK1 | VCP | ANG |
| BDKRB1 | CYP19A1 | GLRA2 | KIT | PABPC1 | ROCK2 | VDR | APOA2 |
| BDKRB2 | CYP1A1 | GNPAT | KLK1 | PADI1 | RORC | VEGFA | DDX6 |
| BHMT | CYP1A2 | GNRHR | KLK2 | PADI2 | RPS6KA1 | VHL | FCAR |
| BLK | CYP1B1 | GPBAR1 | KLKB1 | PADI3 | RPS6KA3 | WEE1 | NUDT9 |
| BMP1 | CYP2A6 | GPR139 | KMO | PADI4 | RPS6KA5 | WNT3A | RTN4R |
| BMP4 | CYP2C19 | GPR35 | KMT2A | PAK1 | RPS6KB1 | XDH | SNRPA |
| BMX | CYP2C9 | GPR84 | LAP3 | PAK4 | RRM1 | XIAP | TGFBR2 |
| BRAF | CYP2D6 | GPR88 | LARS | PAM | RXRA | XPNPEP1 | CLC |
| BRD2 | CYP3A4 | GRB2 | LCK | PARP1 | S1PR1 | XPNPEP2 | HSD17B11 |
| BRD3 | CYSLTR2 | GRIA1 | LDHA | PARP10 | S1PR3 | XPO1 | IMPA1 |
| BRD4 | DAO | GRIA2 | LDHB | PARP2 | S1PR4 | YARS | IMPA2 |
| BRD9 | DAPK1 | GRIA4 | LGALS1 | PBK | S1PR5 | YES1 | LGALS7 |
| BRPF1 | DAPK3 | GRIK1 | LGALS3 | PCNA | SAE1 | YWHAG | METAP1 |
| BRS3 | DBF4 | GRIK2 | LGALS4 | PDE10A | SCD | ZAP70 | TSSK6 |
| BTK | DBH | GRIK3 | LGALS8 | PDE11A | SCN10A | ACMSD | BMP2 |
| C3AR1 | DCLK2 | GRIK5 | LGALS9 | PDE1B | SCN2A | ADIPOQ | GC |
| C5AR1 | DCLK3 | GRIN1 | LIMK1 | PDE2A | SCN5A | ADSL | RORA |
| CA1 | DDAH1 | GRIN2A | LIMK2 | PDE3A | SCN9A | AK1 | DDO |
| CA12 | DDR1 | GRIN2B | LIPC | PDE3B | SELE | AKT1S1 | NR1I3 |
| CA13 | DDR2 | GRK1 | LIPE | PDE4A | SELL | ALDH7A1 | SHH |
| CA14 | DHFR | GRK2 | LIPG | PDE4B | SELP | AOX1 | ADH1A |
| CA2 | DHODH | GRK3 | LNPEP | PDE4C | SERPINA6 | APOA1 | GAK |
| CA3 | DLG4 | GRK5 | LPAR3 | PDE4D | SERPINE1 | APOB | MAPK11 |
| CA4 | DMPK | GRK6 | LRRK2 | PDE5A | SETD7 | ASGR1 | PORCN |
| CA5A | DNM1 | GRM1 | LSS | PDE6A | SETDB1 | ATP2A1 | CTRB1 |
| CA5B | DNM2 | GRM2 | LTA4H | PDE7A | SF3B3 | BCKDHA | HTT |
| CA6 | DNM3 | GRM3 | LTB4R | PDE8B | SGK1 | BCKDHB | PDHB |
| CA7 | DNMT1 | GRM4 | LYN | PDE9A | SHBG | BDNF | MCAT |
| CA9 | DNMT3B | GRM5 | MAK | PDF | SI | BHMT2 | CALM2 |
| CACNA2D1 | DOT1L | GRM6 | MALT1 | PDGFRA | SIGMAR1 | CACNA1C | RNASE1 |
| CALCRL | DPP4 | GRM7 | MAN2B1 | PDGFRB | SIRT1 | CASP9 | CALM3 |
| CALM1 | DPP7 | GRM8 | MANBA | PDK1 | SIRT2 | CAT | F9 |
| CAMK2B | DPP8 | GSK3A | MAOA | PDPK1 | SLC13A5 | CCBL1 | DDIT4 |
| CAMK2D | DPP9 | GSK3B | MAOB | PDYN | SLC15A1 | CCBL2 | TPI1 |
| CAMKK2 | DRD1 | GSR | MAP2 | PEPD | SLC16A1 | CCL20 | YWHAE |
| CAPN1 | DRD2 | GSTA1 | MAP2K1 | PFKFB3 | SLC16A3 | CCL4 | ETFDH |
| CAPN2 | DRD3 | GSTK1 | MAP2K2 | PGA5 | SLC18A2 | CHAT | CYCS |
| CARM1 | DRD4 | GSTM2 | MAP2K7 | PGD | SLC18A3 | CHDH | PRDX5 |
| CASK | DRD5 | GSTP1 | MAP3K11 | PGGT1B | SLC1A1 | CHKA | HRSP12 |
| CASP1 | DTYMK | GUSB | MAP3K14 | PGK1 | SLC1A2 | CHKB | ACSM2B |
| CASP2 | DUSP1 | GYS1 | MAP3K5 | PGR | SLC22A12 | CHKB-CPT1B | ACSM1 |
| CASP3 | DUSP3 | HAO1 | MAP3K8 | PHF8 | SLC22A6 | CLEC10A | RAB9A |
| CASP6 | DUSP4 | HAO2 | MAP3K9 | PI4KB | SLC28A2 | CNOT2 | GLYAT |
| CASP7 | DUT | HCAR2 | MAP4K4 | PIK3C2A | SLC28A3 | COPS2 | NDRG2 |
| CASP8 | DYRK1A | HCK | MAPK1 | PIK3C2B | SLC29A1 | COPS7A | HBA2 |
| CBFB | DYRK1B | HCRTR1 | MAPK10 | PIK3C3 | SLC2A1 | COPS7B | HBB |
| CBR1 | DYRK2 | HCRTR2 | MAPK13 | PIK3CA | SLC37A4 | CREM | SULT1A2 |
| CBX4 | DYRK3 | HDAC1 | MAPK14 | PIK3CB | SLC5A1 | CRYL1 | SDHA |
| CBX7 | EBP | HDAC10 | MAPK15 | PIK3CD | SLC5A2 | CST8 | NPR1 |
| CCKBR | ECE1 | HDAC11 | MAPK3 | PIK3CG | SLC5A4 | CTGF | CES4A |
| CCNA1 | EDNRA | HDAC2 | MAPK7 | PIK3R1 | SLC5A7 | CYP2A13 | CES5A |
| CCNA2 | EDNRB | HDAC3 | MAPK8 | PIM1 | SLC6A11 | CYP2A7 | RNF113B |
| CCNB1 | EED | HDAC4 | MAPK9 | PIM2 | SLC6A12 | DCLRE1B | RNF113A |
| CCNB2 | EGFR | HDAC5 | MAPKAPK2 | PIM3 | SLC6A13 | DDX24 | NIT1 |
| CCNB3 | EGLN1 | HDAC6 | MAPT | PIN1 | SLC6A2 | DPYD | SERPIND1 |
| CCND1 | EGLN2 | HDAC7 | MB | PIP4K2C | SLC6A3 | EDN1 | SERPINC1 |
| CCNE1 | EGLN3 | HDAC8 | MBD2 | PITRM1 | SLC6A4 | EIF4G1 | SLC16A7 |
| CCNE2 | EHMT1 | HDAC9 | MBNL1 | PKN1 | SLC6A9 | EN1 | MED6 |
| CCNT1 | EHMT2 | HEXA | MBNL2 | PLA2G10 | SLC7A5 | EPHX1 |  |

# Supplementary Table 5. Core target list

| gene symbol | gene symbol | gene symbol | gene symbol | gene symbol | gene symbol | gene symbol | gene symbol |
| --- | --- | --- | --- | --- | --- | --- | --- |
| STAT3 | ESR1 | HIF1A | MMP9 | IL1B | HDAC4 | STAT6 | SOD2 |
| AKT1 | MAPK8 | CCND1 | PPARA | RXRA | CXCR4 | GRIN2B | AKT1S1 |
| TP53 | NRAS | MTOR | ERBB3 | FGF2 | PARP1 | DLG4 | PLAU |
| SRC | STAT1 | PDGFRB | NFKB1 | BDNF | CALM3 | CCR5 | TIMP2 |
| MAPK3 | JAK2 | APP | ITGB1 | IGF1R | ILK | CASP2 | ICAM1 |
| MAPK1 | TNF | AR | SERPINC1 | HDAC3 | DUSP1 | PLAUR | HSPA8 |
| HSP90AA1 | IL2 | KDR | CASP3 | PTPN2 | PIK3CB | ITGB2 | ADORA3 |
| EP300 | EDN1 | RAF1 | AKT3 | TERT | INSR | MMP1 | F9 |
| HRAS | RELA | BCL2L1 | NOS2 | BCL2 | CAT | VHL | PTGS1 |
| EGFR | CASP8 | FOXO3 | JAK1 | CCL4 | CYCS | FLT1 | CDK5 |
| JUN | FOS | TGFB1 | ITGB3 | PIK3CG | SERPINE1 | CYP3A4 | CASP7 |
| PTPN11 | MAPK14 | HDAC2 | SIRT1 | FGFR1 | MMP2 | CNR1 | CAMK2D |
| PIK3R1 | HDAC1 | CALM1 | PRKCD | CCL20 | MAPK9 | PRKCA | HDAC7 |
| CTNNB1 | MAP2K1 | RPS6KB1 | BRAF | CASP9 | HSPA5 | GRIA1 | BAD |
| VEGFA | AKT2 | F2 | APOA1 | APOB | XIAP | GRIN1 | AGTR1 |
| PIK3CA | ERBB2 | PPP2CA | GSK3B | RET | SLC9A1 | GRIN2A | STAT4 |
| CREBBP | PLG | NOS3 | KIT | PTGS2 | APOA2 | ELANE | NR4A1 |
| KRAS | PPARG | ALB | ADRBK1 | PDGFRA | CALM2 | TGFBR1 | MMP8 |
| PTK2B | MMP3 | FABP1 | MAP3K5 | IGFBP1 | HK1 |  |  |

# Supplementary Table 6. Top15 metabolic pathways of node degrees in KEGG analysis

| No. | KEGG ID | Name | Pathway Class | Degree |
| --- | --- | --- | --- | --- |
| 1 | hsa05200 | Pathways in cancer | Cancer: overview | 68 |
| 2 | hsa04151 | PI3K-Akt signaling pathway | Signal transduction | 48 |
| 3 | hsa05205 | Proteoglycans in cancer | Cancer: overview | 46 |
| 4 | hsa05161 | Hepatitis B | Infectious disease: viral | 42 |
| 5 | hsa04015 | Rap1 signaling pathway | Signal transduction | 40 |
| 6 | hsa04014 | Ras signaling pathway | Signal transduction | 39 |
| 7 | hsa05215 | Prostate cancer | Cancer: specific types | 35 |
| 8 | hsa04510 | Focal adhesion | Cellular community - eukaryotes | 35 |
| 9 | hsa04010 | MAPK signaling pathway | Signal transduction | 35 |
| 10 | hsa04919 | Thyroid hormone signaling pathway | Endocrine system | 34 |
| 11 | hsa04722 | Neurotrophin signaling pathway | Nervous system | 34 |
| 12 | hsa04066 | HIF-1 signaling pathway | Signal transduction | 33 |
| 13 | hsa05152 | Tuberculosis | Infectious disease: bacterial | 32 |
| 14 | hsa04024 | cAMP signaling pathway | Signal transduction | 32 |
| 15 | hsa05166 | HTLV-I infection | Infectious disease: viral | 32 |
