# Supplementary material for: Transcriptomics, proteomics, metabolomics and network pharmacology reveal molecular mechanisms of multi‐targets effects of Shenxianshengmai improving human iPSC‐CMs beating
Source: Clin Transl Med. 2023 Jun 6;13(6):e1302. doi: 10.1002/ctm2.1302 (PMC10246690; doi:10.1002/ctm2.1302)
Supplement: Supplementary file 5 — Supporting Information [file CTM2-13-e1302-s013.docx]

**Supplementary Figures Legends 1-8**

## Supplementary Figure 1

## Determining the optimal concentration of SXSM for elevation of human iPSC-CMs beating.

(**A**) Immunofluorescence of cardiomyocyte marker cTnT/α-Actinin in human iPSC-CMs. Nuclei were labeled with Hoechst. (**B**) Flow cytometry analysis of cTnT positive in human iPSC-CMs. (**C**) Electrophysiological characteristics of human iPSC-CMs were analyzed by manual patch clamp. Ventricular-like action potential profiles of human iPSC-CMs from the same batch is represented. MDP, -53.14±0.10mV; PEAK, 34.54±0.19mV; Max Decay Slope, 13.38±0.15mV/ms; AMP, 92.55±0.23mV; APD90, 363.83±4.00ms; APD50, 302.33±3.50ms. n=3. (D-F) SXSM dose long term effects. (**D**) Beat Rate (BR, impedance mode); (**E**) Field potential duration corrected by Fridericia’s formula (FPDc, EFP mode); (**F**) Amplitude (Amp, impedance mode). **P < 0.05, **P < 0.01, ***P < 0.001* compared to the normalized baseline (100%, one sample test); *#P < 0.05, ##P < 0.01, ###P < 0.001* compared to the normalized baseline (100%, one sample test). (**G-H**) SXSM dose effects for 1h in impedance mode. (**G**) BR; (**H**) Amp. The sample size (n) is indicated above the horizontal coordinate of each concentration. SXSM original concentration is 1096mg/ml. The concentrations of the horizontal coordinates in the graph from left to right are as follows: 3×10^-3^mg/ml (0.0003% dilution), 0.01mg/ml (0.001% dilution), 0.03mg/ml (0.003% dilution), 0.11mg/ml (0.01% dilution), 0.33mg/ml (0.03% dilution), 0.55mg/ml (0.05% dilution), 0.63mg/ml (0.0571% dilution), 0.73mg/ml (0.0667% dilution), 0.88mg/ml (0.08% dilution), 1.10mg/ml (0.1% dilution), 1.37mg/ml (0.125% dilution), 1.83mg/ml (0.167% dilution), 2.74mg/ml (0.250% dilution), 5.48mg/ml (0.5% dilution), 10.96mg/ml (1.0% dilution), and 109.6mg/ml (10.0%). **P < 0.05, **P < 0.01, ***P < 0.001, ****P < 0.0001* compared to the 0.55mg/ml SXSM group (one-way ANOVA). (**I-J**) Elution effects on the BR/Amp of human iPSC-CMs with SXSM in impedance mode. (**I**) BR; (**J**) Amp. The sample size (n) is indicated above the horizontal coordinate of each concentration. (**K**) Representative trace of BR elevation after 0.55mg/ml SXSM treatment. (**L**) Representative trace of FPD shortening after 0.55mg/ml SXSM administration. All data are presented as the mean ± SE.

## Supplementary Figure 2

## Role of SXSM and isoproterenol (ISO) in the adrenoceptor-related signaling pathway of human iPSC-CMs.

(**A**) Representative traces of FPD shortening at the respective specific concentrations of SXSM and ISO in EFP mode. SXSM (1.1mg/ml, 0.55mg/ml, 0.11mg/ml); ISO (1μM, 100nM, 10nM). **(B-C)** SXSM/ISO dose effects of 1h treatment on FPDc in EFP mode. (**B**) SXSM; (**C**) ISO. (**D-E**) SXSM/ISO dose effects of 1h treatment on BR in EFP mode. (**D**) SXSM; (**E**) ISO. (**B, D**) **P* < 0.05, ***P* < 0.01, ****P* < 0.001 compared to the 0.55mg/ml group (one-way ANOVA). (**C, E**) **P* < 0.05, ***P* < 0.01, ****P* < 0.001, *****P* < 0.0001 compared to the 1μM group (one-way ANOVA). (**F-G**) Effects of different concentrations of propranolol (Propra), nonselective inhibitor of β-adrenergic receptor, on BR/Amp after 1 h of treatment with 0.55mg/ml SXSM in impedance mode. (**F**) BR*. ##P < 0.01, &&P < 0.01, ****P < 0.0001* compared to corresponding 0.55mg/ml SXSM pretreatment in each propranolol treatment group (t-test). (**G**) Amp. **P < 0.05* compared to corresponding 0.55mg/ml SXSM pretreatment in propranolol 10μM group (t-test). (**H-I**) Effects of 5μM propranolol on BR/Amp after SXSM or ISO pretreatment in impedance mode. (**H**) BR; (**I**) Amp. **P < 0.05,* comparison between groups. **(J-K)** Effects of different concentrations of atenolol (Ate), inhibitor of β1-adrenergic receptor, on BR/FPDc after 1 h pretreatment with 0.55mg/ml SXSM in EFP mode. (**J**) BR; (**K**) FPDc. **P* < 0.05, ***P* < 0.01, ****P* < 0.001 comparison between groups (t-test). *&P* < 0.05, *&&P* < 0.01, *&&&P < 0.001, &&&&P<0.0001* compared to the point with the horizontal coordinate “SXSM 0.55mg/ml” in SXSM+Ate doses group (red) (one-way ANOVA). (**L-M**) Effects of different concentrations of atenolol on BR/FPDc after 1 h pretreatment with 1μM ISO in EFP mode. (**L**) BR; (**M**) FPDc. **P* < 0.05, ***P* < 0.01, ****P* < 0.001 comparison between groups (t-test). *&P* < 0.05, *&&P* < 0.01, *&&&P < 0.001, &&&&P<0.0001* compared to the point with the horizontal coordinate “ISO 1μM” in ISO 1μM+Ate doses group (orange) (one-way ANOVA). *#P < 0.05, ##P < 0.01, ###P < 0.001, ####P<0.0001* compared to the point with the horizontal coordinate “ISO 1μM” in ISO 1μM (continuing) group (black) (one-way ANOVA). **(F-M)** Each treatment was spaced 1 hour apart. All data are presented as the mean ± SE.

## Supplementary Figure 3

## Interaction network of compound/disease-related targets and GO functional enrichment analysis on 150 core targets.

(**A-B**) Interaction network of compound/disease-related targets. Node size and color shades are positively correlated with Degree values. (**A**) compound-related targets; (**B**) disease-related targets.

(**C-D**) GO analysis of core targets. (**C**) Biological Process (BP) enrichment; (**D**) Molecular Functional (MF) enrichment.

## Supplementary Figure 4

## The analysis of transcriptomics.

(**A**) clustering heat map for significantly DE genes in CTRL vs. SXSM. The color scale is used to represent the relative gene expression level. Red denotes an uptick, while blue suggests a decrement. The color white, on the other hand, is static. There are two groupings represented by the colored bars atop the map. (**B-E**) Gene Set Enrichment Analysis (GSEA) of detected genes in CTRL vs. SXSM. (**B**) membrane potential related terms in gene ontology (GO) database. (**C**) myosin related terms in GO database. (**D**) angiogenesis and cardiac hypertrophy related terms in GO database. (**E**) VEGF related terms in Reactome database. (**F**) Comparison of AS patterns. Left, schematic representation of AS events. Right, Comparison of detected AS events and significant (p<0.05) AS events.

## Supplementary Figure 5

## The analysis of proteomics.

(**A**) statistical histogram of identification and quantification results. A quantifiable protein was defined as a protein that has quantitative information for more than half of the biological repeats in at least one group. (**B**) Venn diagram of proteins identified across all sample groups. Among the protein collections of SXSM and CTRL groups, 4228 proteins are common to them. The identification of proteins for each sample was well reproducible between the groups. (**C-D**) Venn map of all replicates identified in the sample group. To analyze the reproducibility of protein identification between samples within a group, a Venn diagram was used to analyze the overlap of samples within a group. (**C**) and (**D**) show the overlap of protein sets identified by all biological replicates within the CTRL and SXSM groups, respectively. The identification of proteins for each sample was well reproducible within the groups. (**E**) clustering heat map for significantly DE proteins in CTRL vs. SXSM. The color scale is used to represent the relative protein level. Red denotes an uptick, while blue suggests a decrement. The color white, on the other hand, is static. There are two groupings represented by the colored bars atop the map. Differences in the accumulation patterns of DE proteins in the SXSM and CTRL groups could be analyzed by clustering heat maps, indicating significant differences in substances in the different groups. (**F**) The summary graph of the B-level classification of the KEGG enrichment of DE proteins.

## Supplementary Figure 6

## The analysis of metabolomics.

(**A**) Number of identified metabolites in each chemical class. The color blocks of different colors in the figure represent different chemical classification attribution items, and the percentage represents the percentage of the number of metabolites in the chemical classification attribution entry to the total number of identified metabolites. Metabolites without chemical taxonomic assignment were defined as "undefined". (**B**) clustering heat map for significantly DE metabolites in CTRL vs. SXSM. The color scale is used to represent the relative metabolite level. Red denotes an uptick, while white suggests a decrement. Yellow, on the other hand, is static. There are two groupings represented by the colored bars atop the map. (**C-D**) O2PLS analysis. Loadings plots of O2PLS model integrating DE gene expression (Panel C, X joint loadings) and DE metabolite data (Panel D, Y joint loadings). The relative position of each dot in the two loadings plots indicates if regression coefficients for a given pair of DE gene expression and DE metabolite are correlated or anticorrelated to each other, whereas a longer distance from the origin indicate a stronger correlation. Variables with top25 loading value in each dataset are labeled (red points).

## Supplementary Figure 7

## Effects of 0.55mg/ml SXSM on sodium/potassium channels in human iPSC-CMs.

(**A-B**) Effects of different concentrations of flecainide, inhibitor of Na^+^ channels, on BR/Amp after 1 h pretreatment with 0.55mg/ml SXSM in impedance mode. (**A**) BR; (**B**) Amp. **P* < 0.05, ***P* < 0.01, ****P* < 0.001 comparison between groups (t-test). *&P* < 0.05, *&&P* < 0.01, *&&&P < 0.001, &&&&P<0.0001* compared to the point with the horizontal coordinate “SXSM 0.55mg/ml” in SXSM+Flecainide doses group (red) (one-way ANOVA). Nav channel antagonist flecainide itself significantly depressed the baseline BR of human iPSC-CMs with increasing concentration, and additionally depressed the 0.55mg/ml SXSM-induced elevation of BR in a concentration gradient-dependent manner. Amp was slightly inhibited by 0.55mg/ml SXSM treatment in human iPSC-CMs and seemed to be reversed this by flecainide 300nM/1μM. Meanwhile, elevation of BR by SXSM was also significantly repressed by 1μM flecainide (116.8% ± 1.0%) and by 3μM flecainide (95.8% ± 1.9%). Flecainide 3μM and above alone had a suppressive effect on beating of baseline. (**C-D**) Effects of different concentrations of ML133, inhibitor of Kir2.1, on BR/Amp after 1 h pretreatment with 0.55mg/ml SXSM in impedance mode. (**C**) BR; (**D**) Amp. **P* < 0.05, ***P* < 0.01, ****P* < 0.001 comparison between groups (t-test). Kir2.1 inhibitor ML133 <3 μM did not yet significantly affect the elevation of BR in 0.55mg/ml SXSM 1h-treated human iPSC-CMs, but 3μM ML133 significantly suppressed this elevation of BR (113.5% ± 3.8%) and reversed slight suppression of Amp (98.5% ± 4.2%). (**E-F**) Effects of different concentrations of dofetilide (Dof), selective blocker of the Kv11.1 (hERG) channel, on BR/FPDc after 1 h pretreatment with 0.55mg/ml SXSM in EFP mode. (**E**) BR; (**F**) FPDc. **P* < 0.05, ***P* < 0.01, ****P* < 0.001 comparison between groups (t-test). *&P* < 0.05, *&&P* < 0.01, *&&&P < 0.001* compared to the point with the horizontal coordinate “SXSM 0.55mg/ml” in SXSM+Dof doses group (red) (one-way ANOVA). Dof, selective blocker of the Kv11.1 (hERG) channel, dose dependently inhibit the BR elevation of human iPSC-CMs pretreated by 0.55mg/ml SXSM for 1h. Dof 10nM alone showed prolongation effect on FPDc without effect on baseline BR. Post-treatment of Dof at 10nM, FPDc was inevitably prolongated, but the BR of human iPSC-CMs elevated by SXSM was significantly suppressed (127.5% ± 3.9%). (**G-H**) Effects of different concentrations of chromanol 293B, inhibitor of Kv7.1 on BR/Amp after 1 h pretreatment with 0.55mg/ml SXSM in impedance mode. (**G**) BR; (**H**) Amp. Kv7.1 channel antagonist chromanol 293B treatment with a concentration gradient of 3μ/10μ/30μM had no significant effect on the baseline BR of human iPSC-CMs, and chromanol 293B pretreatment had no significant effect on the alteration of BR and Amp of human iPSC-CMs due to 0.55mg/ml SXSM. (**A-H**) Each treatment was spaced 1 hour apart. All data are presented as the mean±SE.

## Supplementary Figure 8

## Long-term effects and cardioprotective effects of SXSM on human iPSC-CMs.

(**A-B**) SXSM dose long term effect. (**A**) BR (impedance mode); (**B**) FPDc (EFP mode). *&P < 0.05, &&P < 0.01, &&&P < 0.001* compared to the 100% baseline in 0.55mg/ml group (one sample test). *#P < 0.05, ##P < 0.01, ###P < 0.001* compared to the 100% baseline in 1.10mg/ml group (one sample test). A long time course effect (≥18h) on the elevation of BR of human iPSC-CMs and the shortening of FPDc were observed in the SXSM group. (**C-D**) Effects of hypoxia and reoxygenation on BR/Amp after 1 hour of pretreatment with 0.55mg/ml SXSM in impedance mode. (**C**) BR; (**D**) Amp. **P < 0.05, **P < 0.01, ***P < 0.001, ****P < 0.0001* comparison between groups (t-test). *&P < 0.05, &&P < 0.01, &&&P < 0.001, &&&&P<0.0001* compared to the 100% baseline in SXSM pretreatment group (one sample test). *#P < 0.05, ##P < 0.01, ###P < 0.001* compared to the 100% baseline in medium group without SXSM pretreatment (one sample test). 0.55mg/ml SXSM 1h pretreatment presented a protective effect on human iPSC-CMs after hypoxia/reoxygenation. The BR of SXSM group gradually decreased with the extension of hypoxia time within 6hrs, but its BR was still relatively higher than that of the medium group at the same time point (SXSM group at 6h timepoint: 66.3% ± 3.6%; medium group at 6h timepoint: 47.4% ± 12.0%). After reoxygenation for 1h, the BR in both groups quickly returned to the pre-hypoxia level (SXSM group: 130.8% ± 3.4%; medium group:108.4% ± 3.9%). In terms of Amp, it was dramatically declined in both group with the extension of hypoxia time within 6hrs, but it was still higher in SXSM group at 6h timepoint (SXSM 0.55mg/ml: 31.1% ± 6.7%; medium group: 11.0% ± 5.8%). After reoxygenation at 1hr, the Amp almost returned in the SXSM-pretreated group (93.9% ± 2.7%), but only partially returned in the medium group (62.0% ± 10.3%). With the extension of reoxygenation time within 18hrs, the Amp in the SXSM treatment group was always higher than that of the medium group at the same time. All data are presented as the mean ± SE.
