# Supplementary figures and images for: Transcriptomics, proteomics, metabolomics and network pharmacology reveal molecular mechanisms of multi‐targets effects of Shenxianshengmai improving human iPSC‐CMs beating
Source: Clin Transl Med. 2023 Jun 6;13(6):e1302. doi: 10.1002/ctm2.1302 (PMC10246690; doi:10.1002/ctm2.1302)

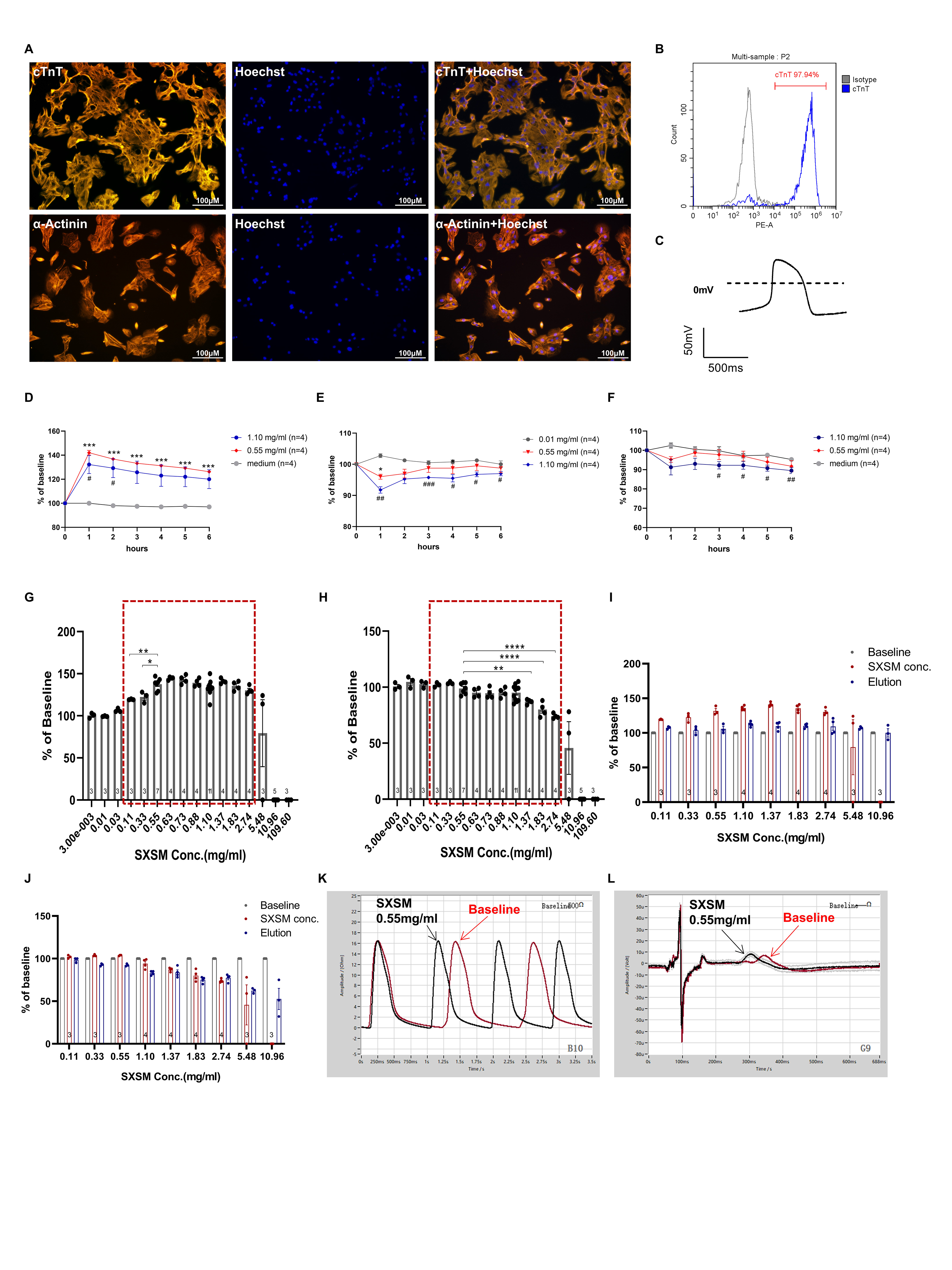

Supplement: Supplementary file 6 — Supporting Information [file CTM2-13-e1302-s001.jpg]

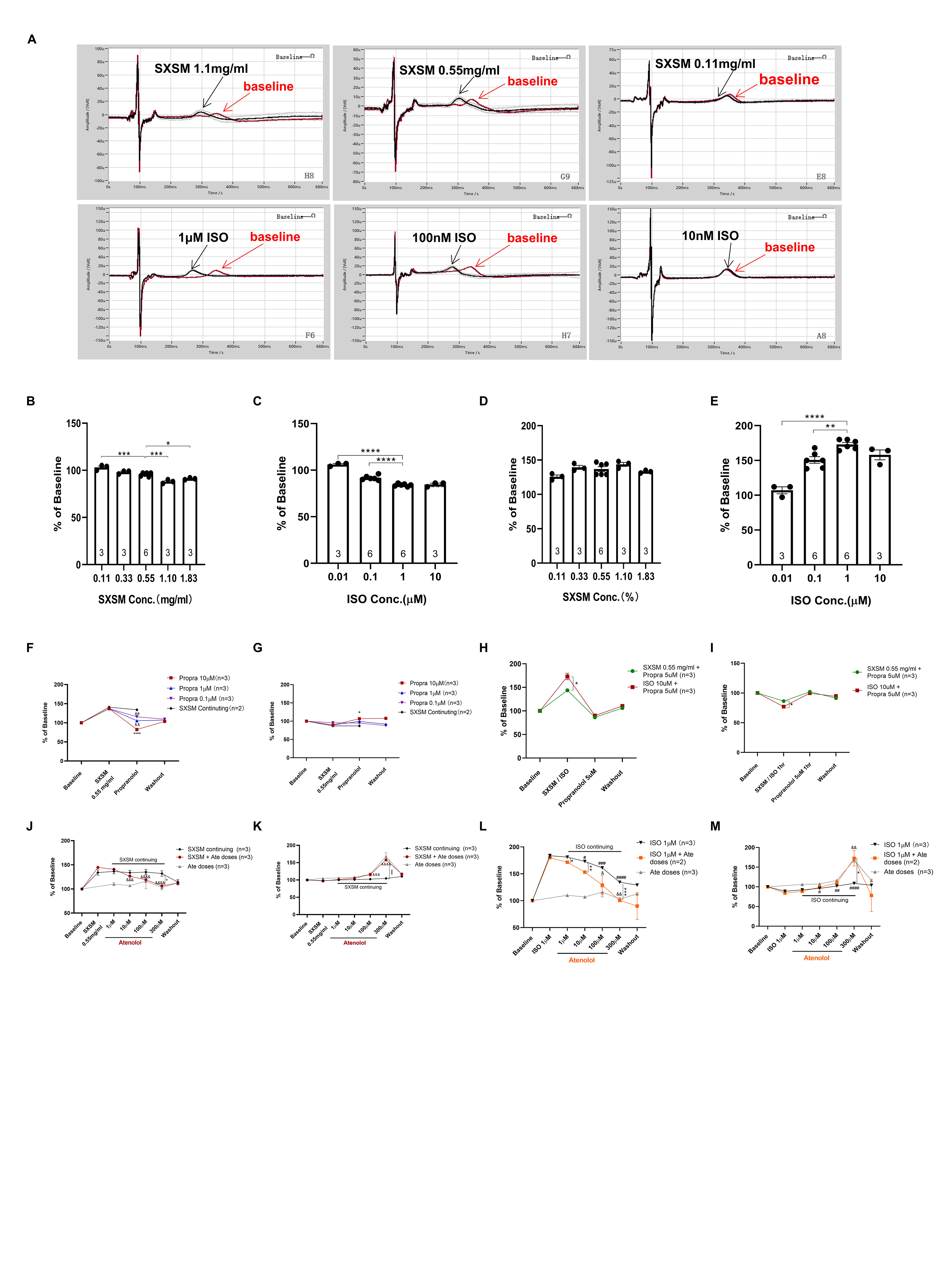

Supplement: Supplementary file 7 — Supporting Information [file CTM2-13-e1302-s010.jpg]

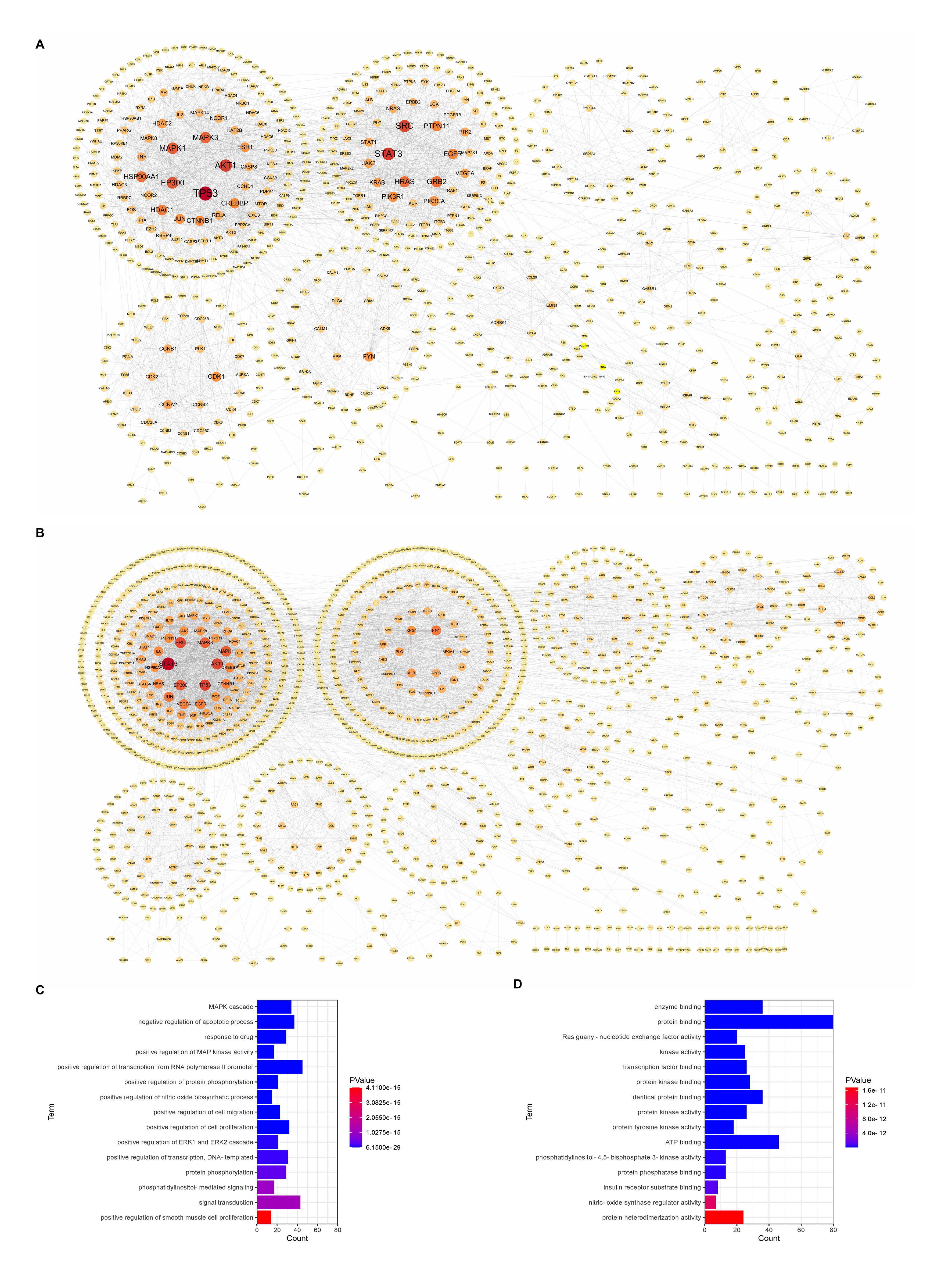

Supplement: Supplementary file 8 — Supporting Information [file CTM2-13-e1302-s004.jpg]

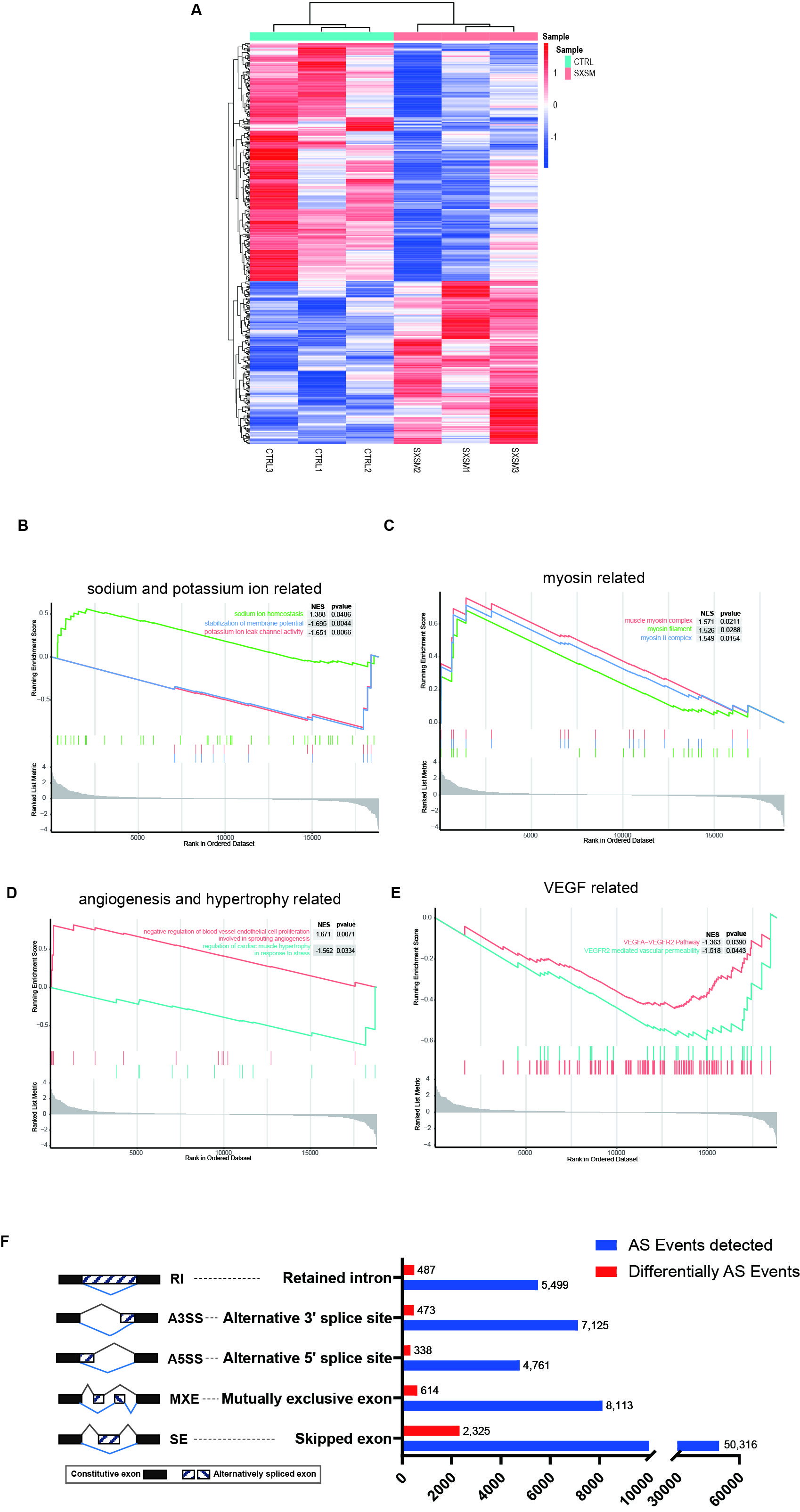

Supplement: Supplementary file 9 — Supporting Information [file CTM2-13-e1302-s007.jpg]

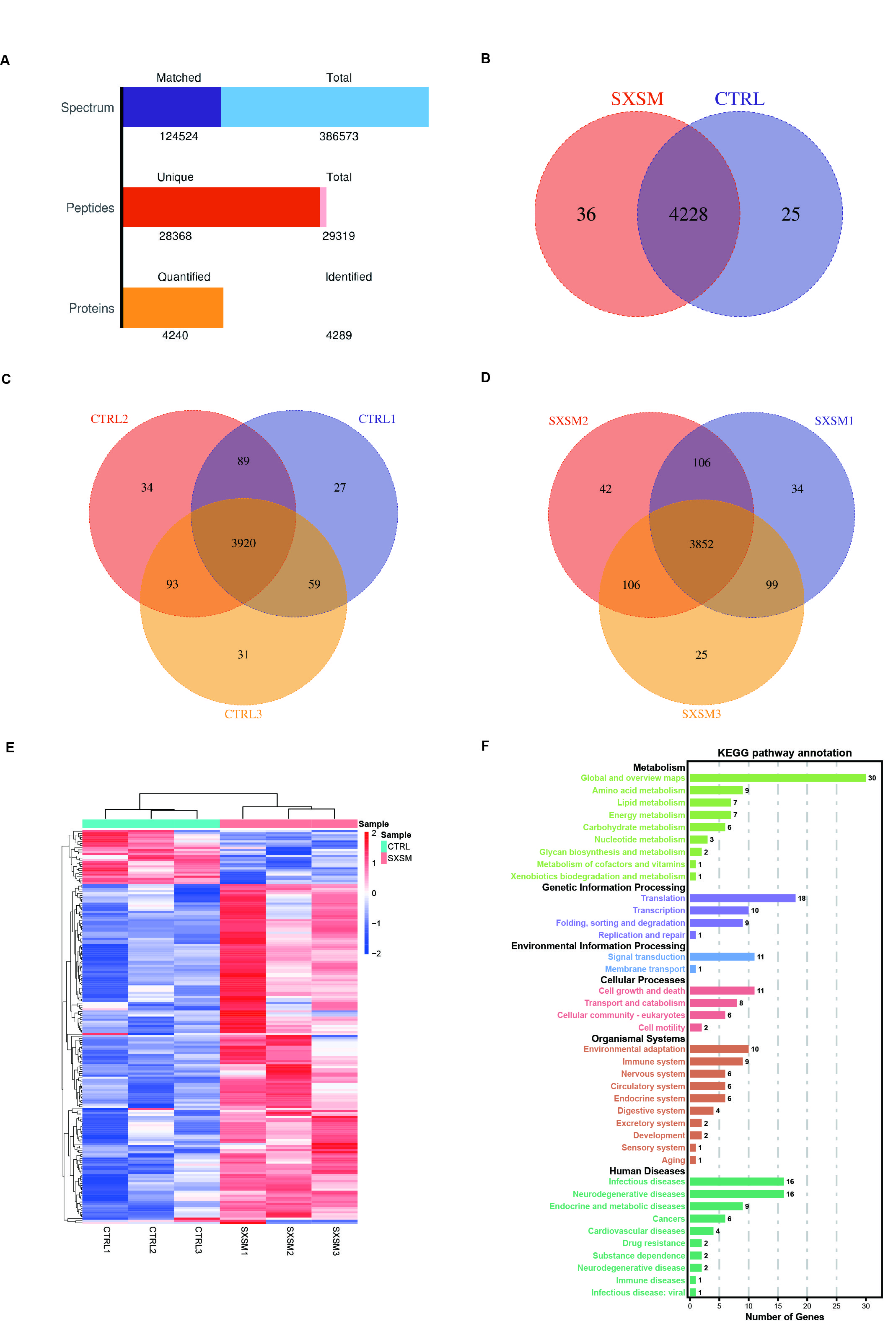

Supplement: Supplementary file 10 — Supporting Information [file CTM2-13-e1302-s005.jpg]

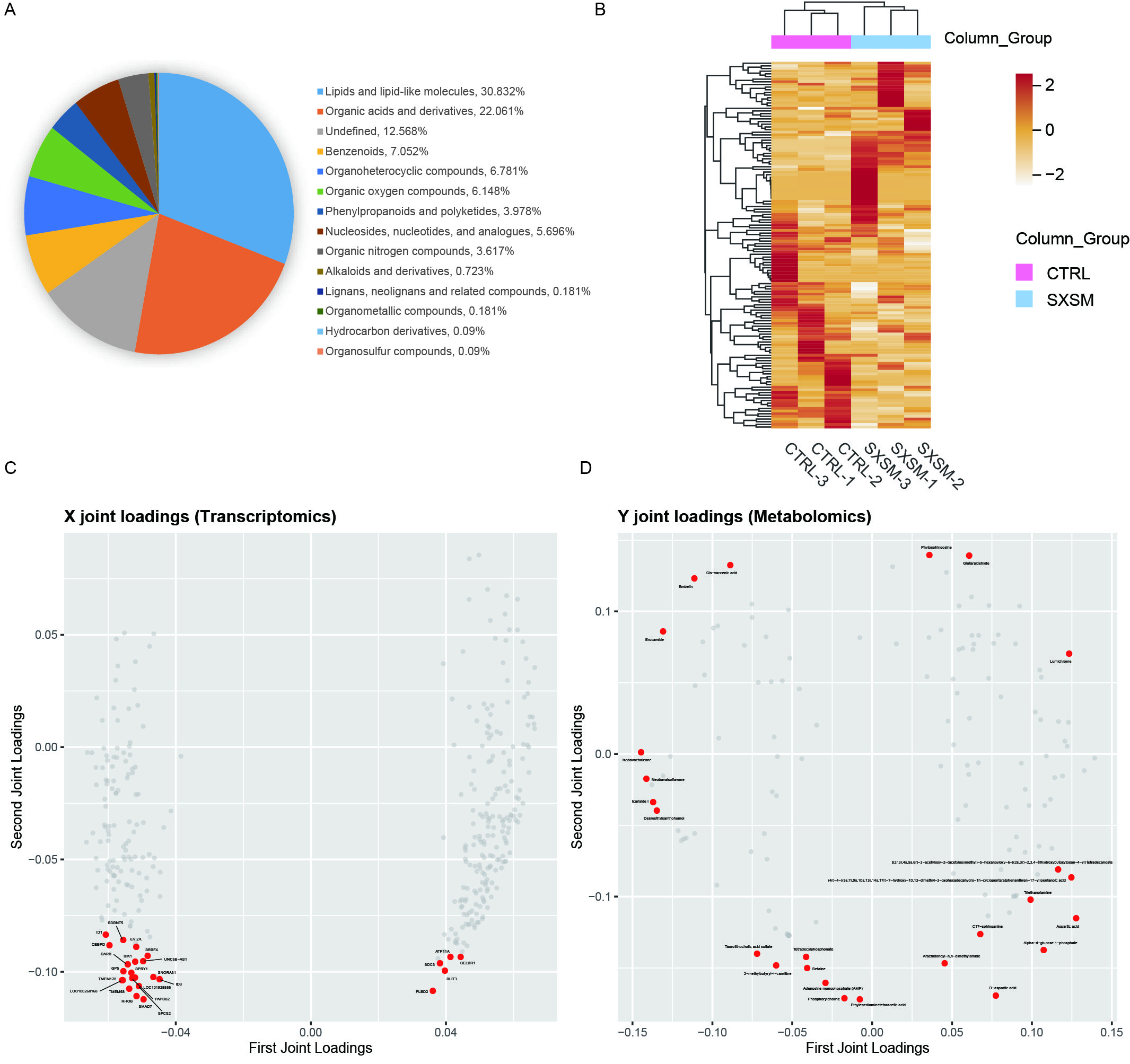

Supplement: Supplementary file 11 — Supporting Information [file CTM2-13-e1302-s003.jpg]

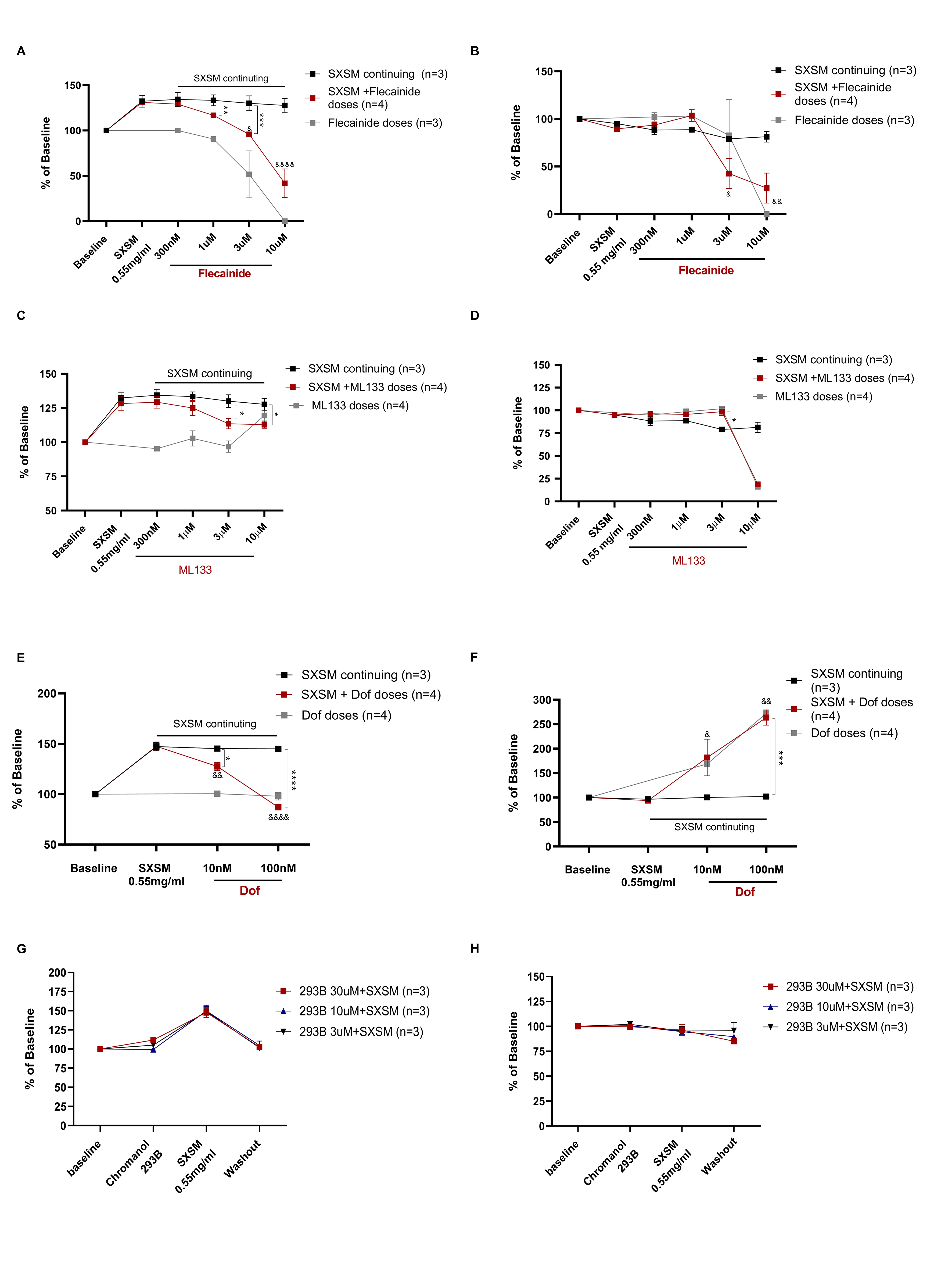

Supplement: Supplementary file 12 — Supporting Information [file CTM2-13-e1302-s012.jpg]

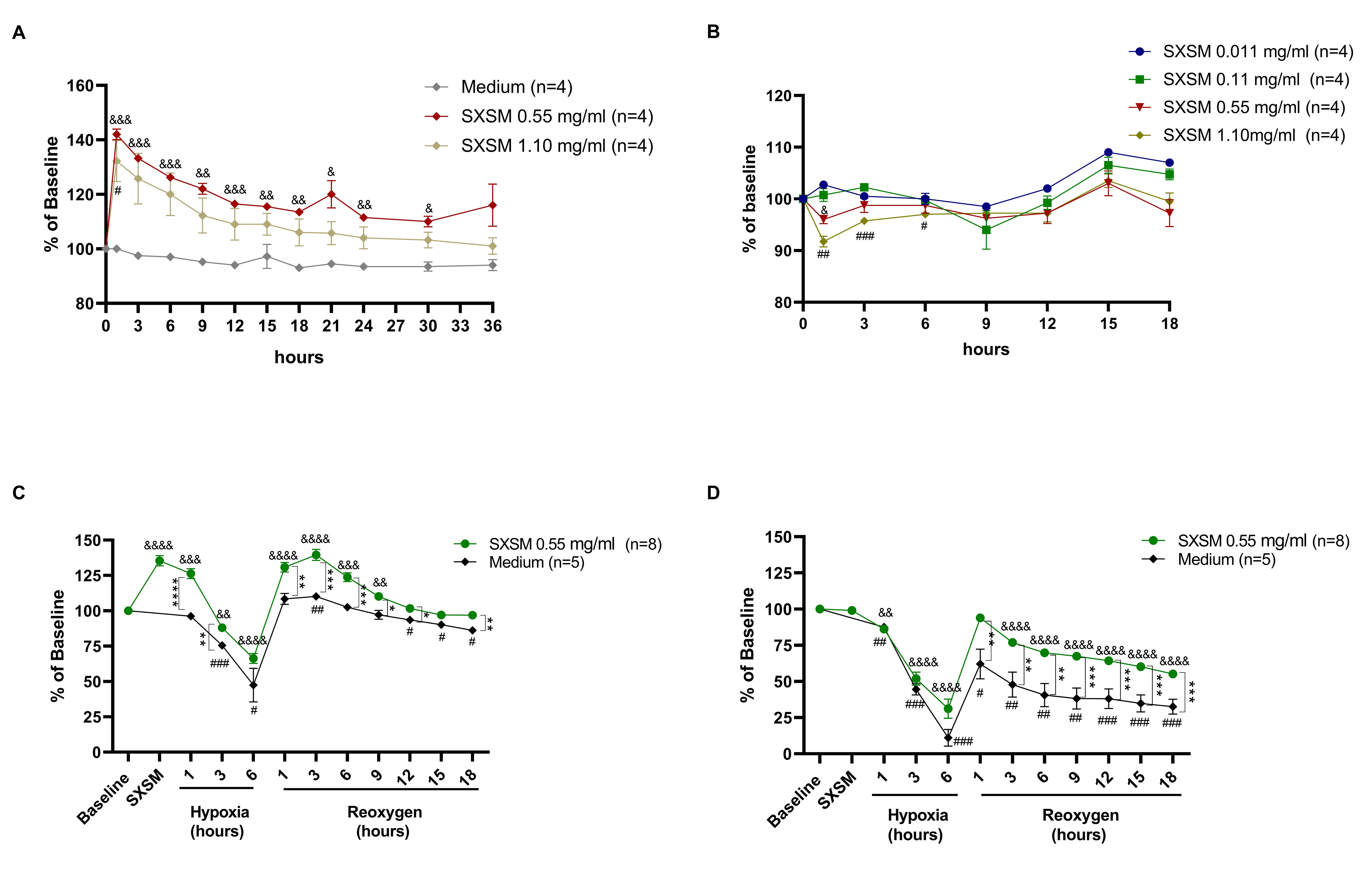

Supplement: Supplementary file 13 — Supporting Information [file CTM2-13-e1302-s011.jpg]
